# Dating the Origin and Spread of Plastids and Chromatophores

**DOI:** 10.3390/ijms26125569

**Published:** 2025-06-11

**Authors:** Filip Pietluch, Paweł Mackiewicz, Katarzyna Sidorczuk, Przemysław Gagat

**Affiliations:** 1 Department of Bioinformatics and Genomics, Faculty of Biotechnology, University of Wroclaw, 50-383 Wroclaw, Poland or filip.pietluch@pwr.edu.pl (F.P.); pamac@smorfland.uni.wroc.pl (P.M.); katarzyna.sidorczuk2@uwr.edu.pl (K.S.); 2 Department of Biomedical Engineering, Faculty of Fundamental Problems of Technology, Wroclaw University of Science and Technology, Wybrzeze Wyspianskiego 27, 50-370 Wroclaw, Poland

**Keywords:** Archaeplastida, endosymbiosis, molecular clock, *Paulinella*, phylogeny, plastid evolution

## Abstract

Photosynthetic eukaryotes have shaped the Earth’s biosphere by producing oxygen and organic compounds using light energy in specialized organelles called plastids. Plastids evolved from free-living cyanobacteria ingested by heterotrophic unicellular eukaryotes. Two such independent engulfment processes, called cyanobacterial endosymbioses, have been reported. The first gave rise to primary plastids and three Archaeplastida lineages: glaucophytes, red algae, and green algae with land plants, whereas the second resulted in chromatophores in the rhizarian amoeba *Paulinella*. Importantly, Archaeplastidans donated their plastids to many protist groups, further spreading photosynthesis across the tree of life. To reveal complex plastid evolution, we performed comprehensive phylogenetic and molecular clock analyses using new fossil calibrations and the largest number yet of plastid-encoded proteins from 108 taxa, representing diverse photosynthetic organisms. Our results indicate that primary plastids evolved prior to 2.1–1.8 Ga, i.e., before glaucophytes diverged from other Archaeplastidans, and *Paulinella* chromatophores were likely before 292–266 Ma. Red and green algae were engulfed by cryptophyte and chlorarachniophyte ancestors between 1.7–1.4 Ga and 1.1–1.0 Ga, respectively; the former subsequently triggered plastid transfers to other eukaryotes. We also examined the impact of molecular clocks and calibration sets on age estimates, showing that clocks are the main source of variation.

## 1. Introduction

Around 2.45 billion years ago (Ga), the Earth’s atmosphere started to change due to the accumulation of molecular oxygen. The so-called Great Oxidation Event fundamentally altered life conditions on our planet and was a byproduct of photosynthesis performed by cyanobacteria. Importantly, cyanobacteria not only evolved oxygenic photosynthesis but also transferred this ability to eukaryotes through endosymbiosis [1,2].

The first cyanobacterial endosymbiosis, i.e., the engulfment and integration of a cyanobacterium into the eukaryotic cell, involved a *Gloeomargarita*-like species and an unknown protist [3]. This endosymbiotic merger gave rise to three photosynthetic lineages of Archaeplastida: Glaucophyta (glaucophytes), Rhodophyta (red algae) and Chloroplastida (green algae and land plants). Their cyanobacteria-derived photosynthetic organelles are called, respectively, muroplasts, rhodoplasts and chloroplasts, and collectively primary plastids [4]. Two primary plastid-containing lineages, red and green algae, subsequently triggered evolutionary radiation via a series of eukaryote-to-eukaryote endosymbioses that resulted in a multitude of secondary and higher-order plastids present in cryptophytes, haptophytes, euglenids, chlorarachniophytes, dinoflagellates, stramenopiles, and even parasitic apicomplexans [5].

The second cyanobacterial endosymbiosis involved the ancestor of all photosynthetic *Paulinella* species: *P*. *chromatophora*, *P*. *micropora* and *P*. *longichromatophora*. The *Paulinella* genus, classified within Rhizaria, comprises both photoautotrophic and heterotrophic species of testate filose amoeba [6,7]. The most important feature of the photosynthetic *Paulinella* are blue-green bodies called chromatophores, which probably evolved from a picocyanobacterium of the *Prochlorococcus*/*Synechococcus*/*Cyanobium* clade. Compared to primary plastids, chromatophores represent cyanobacteria-derived organelles at an earlier stage of endosymbiosis, and they are the reason why *Paulinella* is so important from the evolutionary point of view [8].

While the emergence of photosynthetic *Paulinella* is an evolutionary curiosity, the emergence and radiation of Archaeplastida have substantially shaped the Earth’s biosphere. They have participated in carbon dioxide fixation, oxygen production and other global biogeochemical cycles for hundreds of millions of years, becoming the main primary producers in many ecosystems and the largest component of biomass on Earth [9].

Given their global importance, Archaeplastidans have been extensively studied for many decades. However, there are still some fundamental inconsistencies around their origin, e.g., the diversification time and branching order among the Archaeplastida lineages or even the number of cyanobacterial endosymbioses that triggered their evolution [10,11]. These inconsistencies result from the fact that (i) Archaeplastidans poorly preserve in a fossil state, (ii) the phylogenetic signal contained in their genomes has eroded to a large extent, and (iii) glaucophytes have been notoriously underrepresented in phylogenetic and molecular clock studies.

In order to bring us closer to resolving the contentious issues about Archaeplastida evolution, researchers have constructed phylogenetic and molecular clock trees using various molecular markers, taxa, calibration points and methods (Table S1). As a result, the obtained chronograms are difficult to directly compare because they represent different topologies, i.e., Glaucophyta, Rhodophyta or Chloroplastida as the first branching Archaeplastida lineage. Nevertheless, these chronograms do inform us about the diversification times of extant groups of Archaeplastida, though the dates vary considerably among the authors, and the differences sometimes exceed one billion years (Table S1). Generally, most researchers agree that Archaeplastida evolved prior to ~1.6 Ga, Rhodophyta prior to ~1.2 Ga, and Chloroplastida prior to ~1.0 Ga.

In this study, we investigate the diversification time and branching order among the photosynthetic organelles of Archaeplastida, some of their secondary descendants, and for the first time, perform phylogenetic analyses using multiple molecular clock models across all *Paulinella* photosynthetic species. We constructed 12 phylogenies and 18 chronograms using 30 plastid-encoded proteins. The obtained molecular clocks were used to verify tentatively assigned cyanobacteria and Archaeplastida microfossils. In order to check the influence of calibration constraints on chronograms, we tested three calibration sets, including one with the recently discovered oldest fossils of multicellular red alga *Rafatazmia chitrakootensis* (~1.6 Ga) [12] and green alga *Proterocladus antiquus* (~1.0 Ga) [13]. For each calibration set, we also evaluated the discrepancies in age estimations by different clocks.

## 2. Materials and Methods

### 2.1. Data Set Preparation

We used carefully selected 30 conserved plastid-encoded proteins (Table S2) from five organisms, *Gloeomargarita lithophora*, *Cyanobium* sp., *Cyanophora paradoxa*, *Galdieria sulphuraria*, and *Arabidopsis thaliana*, for homolog searches by PSI-BLAST (e-value: <0.001, number of iterations: 5, word size: 2) against amino acid sequences encoded by plastids, cyanobacteria and chromatophore genomes [14]. They represent genes that are shared across all major plastid-bearing lineages, ensuring broad taxonomic representation while minimizing missing data. The plastid proteins were retrieved from the NCBI RefSeq database [15], whereas cyanobacteria and chromatophore proteins were from GenBank [16]. The final set included 108 representatives of cyanobacteria and plastid-carrying eukaryotes (mainly Archaeplastidans). Each contained the full number of markers, with the exception of chlorarachniophytes, which lacked the CCSA protein; however, the absence of one marker should not significantly affect the overall phylogenetic analysis. Each protein group was aligned in MAFFT v7.429 using a slow and accurate L-INS-i algorithm [17]. The multiple sequence alignments were inspected in AliView [18], and the sites best suited for phylogenetic analyses were selected with the TrimAl gappyout method [19]. Since individual plastid proteins are often too short to produce well-resolved trees and can cause a stochastic error, we concatenated all 30 trimmed amino acid alignments into the supermatrix of 9823 positions with SequenceMatrix 1.8 [20]. This is a commonly accepted approach because the plastid genes represent a single coalescent marker, i.e., they are linked and inherited on one molecule and should demonstrate the same evolutionary history [21].

### 2.2. Phylogenetic and Molecular Clock Analyses

The phylogenetic trees were obtained based on (i) the maximum likelihood (ML) method in IQ-TREE 2.0 [22] and RAxML 8.2.12 [23], as well as (ii) the Bayesian method in PhyloBayes MPI 1.7a [24] and, along with the molecular clock analyses, in MrBayes 3.2.7a [25] and Beast 2.6.0 [26]. The ML and Bayesian approaches represent two different but preferentially used methods for phylogeny estimation and both are able to tackle big data sets with parameter-rich models. Additionally, the inclusion of many methods ensured that our conclusions were model-unbiased and provided consistency with prior studies. We did not use summary coalescent methods such as ASTRAL because a plastome tree built on a concatenated set is generally always better supported than that relying on individual gene trees [21].

In order to deal with potential data heterogeneity (different substitution patterns and rates across sites), we considered all 30 potential partitions, represented by each protein group, to find the best substitution models for the concatenated set. To estimate the best-fitted substitution models, we used ModelFinder [27] in IQ-TREE and PartitionFinder [28] for RAxML, MrBayes and Beast (Tables S3–S6). In PhyloBayes, we estimated phylogenies under the CAT-GTR+Γ model to account for the among-lineage and among-site heterogeneity without the necessity of defining partitions. Branch support was inferred based on 100 replicates of non-parametric bootstraps in IQ-TREE and RAxML and the estimation of posterior probabilities in MrBayes, PhyloBayes and Beast.

To test the assumptions of stationarity (constant amino-acid frequencies over time) and homogeneity (constant substitution rates over time) in our data, we performed tests of symmetry in IQ-TREE [29] using Bonferroni correction for multiple testing. In the case of MrBayes and RAxML data sets, 18 out of 19 partitions passed the test, and for Beast, it was 21 out of 22; in total, 87% of the supermatrix sites passed the test. In IQ-TREE, eight of nine partitions passed the test, which accounted for 63% of the supermatrix sites. Accordingly, in most cases, the lineage-specific heterogeneity and non-stationarity concerned only a small part of our data sets. Moreover, we used only relaxed molecular clock methods (see below) that decouple amino acid substitution rates between descendant lineages, thereby further handling the problem of heterogeneity.

In total, we calculated 18 chronograms for three calibration sets, C1, C2 and C3, that represent the first, second and third calibration sets, respectively (Table 1 and Table S7), and explored six molecular clock approaches. C1 was similar to the calibration strategy by Sánchez-Baracaldo et al. [30]. C2 and C3 differ from C1 in using the microfossil of *P. antiquus* to calibrate the minimum age for node 10. In turn, C2 varies from C3 in calibration for node 6; the former uses widely recognized *Bangiomorpha pubescens* [31], while the latter much older and recently discovered *R. chitrakootensis*. Further small differences between C1 and the other calibration sets result from the fact that for C2 and C3, we always applied the minimum or maximum constraints on nodes by selecting the lower or upper interval for microfossil dating, respectively.

We performed Bayesian molecular dating with autocorrelated log normal model (LN), Cox–Ingersoll–Ross model (CIR) and uncorrelated gamma multiplier model (UGAM) in PhyloBayes 4.1c [50]; autocorrelated Thorne–Kishino 2002 model (TK02) and uncorrelated independent gamma rate model (IGR) in MrBayes; as well as uncorrelated lognormal model (UCLNR) in Beast. Consequently, we implemented two different relaxed molecular clock methods, autocorrelated and uncorrelated, which assume a dependent or independent rate of evolution on adjacent branches of the phylogenetic tree, respectively. For the uncorrelated models, the rates across the tree are drawn from a single underlying distribution: lognormal (UNCLNR) or gamma (UGAM and IGR).

In PhyloBayes, we used the fixed tree topology from IQ-TREE because it was congruent with most inferred phylogenies (from MrBayes, Beast, RAxML) and calculated the molecular dating under LN, CIR and UGAM models coupled with a CAT-GTR+Γ model. We selected *Gloeobacter kilaueensis* as an outgroup. For each molecular clock and calibration set, two replicate chains were run until convergence was reached. Analyses were performed using hard-bounded gamma-distributed prior for the root and uniform hard-bounded priors for the rest of the calibration constraints. We selected burnin to obtain the maximum discrepancy lower than 0.3 and the minimum effective size greater than 50 in tracecomp. Mean dates were assessed by running readdiv with burnin selected for each analysis in tracecomp.

In Beast, we implemented the Yule tree prior and exponential priors on calibration constraints because the Yule model was favored over the birth–death model according to Akaike Information Criterion for all chronograms. The testing was conducted using R software (Version 4.0.2 (June, 2020)) with pureBirth and bd functions from the laser package. Tree samples were saved every 1000 iterations. The first 25% of samples were discarded as burnin to obtain an effective sample size greater than 100 for all parameters calculated in Tracer 1.7. The final chronograms were obtained in Treeannotator using 25% burnin and mean heights.

In MrBayes, offset exponential priors were used for divergence times together with a birth–death tree prior. Samples were saved every 100 iterations. To summarize the results, we used the sump command, discarding 25% of the first samples. We made sure that the potential scale reduction factor was close to 1 and that the average effective sample size was greater than 100 for all parameters. The final chronograms were obtained in Treeannotator using 25% burnin and mean heights.

### 2.3. Comparison of Molecular Clocks and Calibration Sets

In order to assess a potential bias in the estimation of divergence times among molecular clocks and calibration sets, we calculated for each set the percentage difference from the mean age of all sets obtained for each node, and next the average differences across all nodes D according to the equation.

$$D=\frac{\sum_{i=1}^{n}100\%\cdot \left(x_{i}-{\bar{x}}_{i}\right)/{\bar{x}}_{i}}{n}$$

where $x_{i}$ is an age for the node i, ${\bar{x}}_{i}$ is a mean age of sets for the node i and n is the number of nodes. In the analysis, we included 104 nodes and excluded three that were not shared in all tree topologies. The calculations were performed separately for (i) different calibration sets assuming the same molecular clock as well as (ii) for each calibration set with different clocks.

Moreover, we calculated the mean pairwise difference in ages across all nodes between individual sets P according to the equation.

$$P=\frac{\sum_{i=1}^{n}\left(x_{i}-y_{i}\right)}{n}$$

where $x_{i}$ is an age in the set x for the node i, $y_{i}$ is an age in the set y for the node i and n is the number of nodes. Similarly, we calculated the mean percentage pairwise difference:

$$P\%=\frac{\sum_{i=1}^{n}100\%\cdot \left(x_{i}-y_{i}\right)/0.5\cdot \left(x_{i}+y_{i}\right)}{n}$$

These values were used for multidimensional scaling (MDS) in R to compare age estimations under various assumptions in a graphical way. The statistical significance of differences between the compared sets was assessed using the paired Wilcoxon test. The resulting *p*-values were corrected using the Benjamini–Hochberg method.

## 3. Results and Discussion

### 3.1. Phylogenetic Analyses

Our analyses strongly support the monophyly of all plastids, which is common in phylogenies based on plastid markers [10,11], but with the exclusion of much recently and independently acquired chromatophores of *Paulinella* species (Figure 1 and Figures S1–S20). Both photosynthetic organelles of Archaeplastida and *Paulinella* diverged from early-branching freshwater cyanobacteria. The former groups with *Gloeomargarita lithophora* and the latter with *Cyanobium gracile*, consistent with previous studies [3,51].

Across all calculated phylogenies, except the PhyloBayes CAT-GTR tree (Figure S20), Glaucophyta formed the first branching lineage to the sister group of Rhodophyta and Chloroplastida; Bayesian trees strongly supported this topology, but the maximum likelihood methods did not (Figure 1 and Figures S1–S19).

Notably, among chloroplasts, *Chlorokybus* and *Mesostigma* broke up the monophyly of Streptophyta (freshwater green algae and land plants) in all our phylogenies, and they were placed at the base of Chloroplastida with strong or moderate support (Figure 1 and Figures S1–S20), similar to Sánchez-Baracaldo et al. [30]. Consequently, these two green algae possibly branched before the split of Streptophyta and Chlorophyta (mainly marine green algae). This position was, however, questioned by Lemieux et al. [52]. They recovered a similar topology in the maximum likelihood trees on the set of 45 plastid proteins but not on the same set of nucleotide sequences and amino acid sequences enriched in slow-evolving sites that supported the monophyly of Streptophyta with Chlorokybus and Mesostigma.

As expected, Chlorarachnea, filose amoebae classified to the superassemblage Rhizaria, branched confidently within Chlorophyta (mainly marine green algae), though their exact position in the PhyloBayes CAT-GTR tree was weakly supported (Figure S20). Chlorarachniophytes carry photosynthetic structures derived from chlorophytes and still retain within their complex plastids a vestigial nucleus (nucleomorph) preserved from the reduced endosymbiont [53].

**Figure 1:**
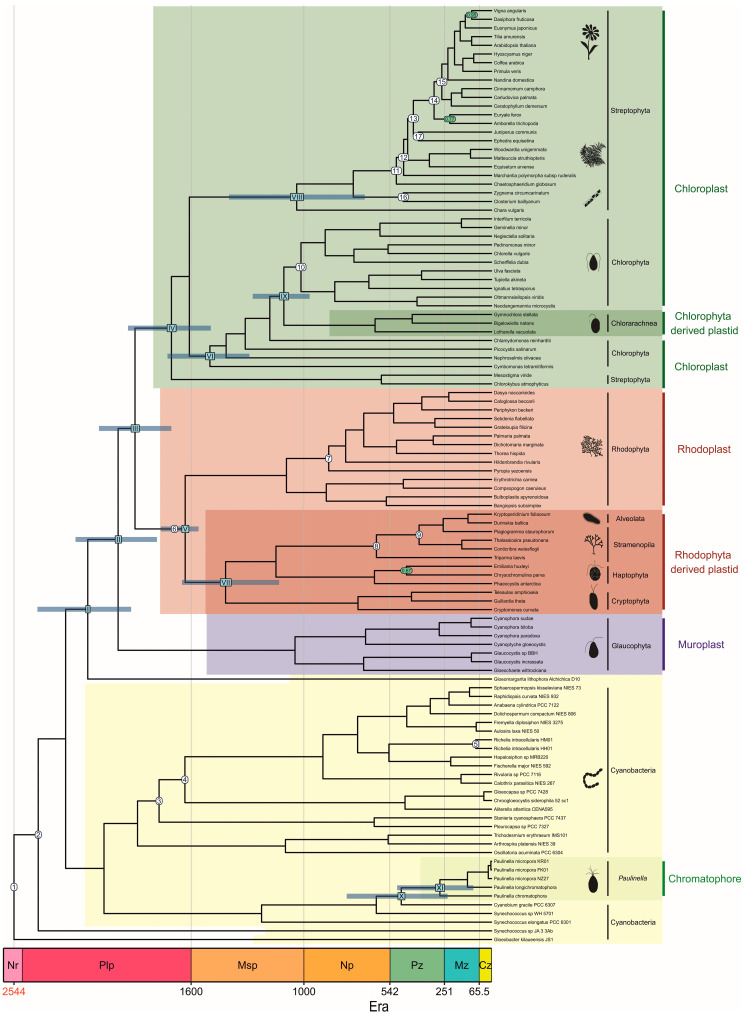
Time-calibrated phylogeny of photosynthetic organelles and cyanobacteria. The tree was inferred with Beast under UCLNR model and calibrated with data set C3 (Table 1). Arabic numerals in white circles indicate the calibration constraints. The Roman numerals in blue rectangles mark key evolutionary events for plastids discussed in the article. At these nodes, there are blue bars representing 95% credibility intervals of the node age. The nodes supported with posterior probability lower than one are indicated with green circles.

Nucleomorph is not only characteristic of Chlorarachnea but also Cryptophyta; however, both organelles were acquired independently from green and red algae, respectively. The up-to-date scenarios for the evolution of red-alga-derived plastids mostly agree that there was a single secondary endosymbiosis beginning with cryptophytes and a few subsequent serial plastid acquisitions (see [54] and citations therein). Accordingly, cryptophytes group with Rhodophyta in all our trees, together with other red-alga-derived plastids of Haptophyta, Stramenopila and Alveolata (dinoflagellates), and with strong statistical support (Figure 1 and Figures S1–S20). Importantly, our recent phylogenies, which focus exclusively on red algae and their derived lineages and, moreover, are based on three times as many plastid markers, challenge the monophyly of cryptophytes, haptophytes, stramenopiles, and alveolates. Instead, they suggest two independent secondary red algal endosymbioses: one within Cryptophyta and another within Stramenopila [55]. According to the model by Pietluch et al. [55], cryptophytes donated their plastids next to haptophytes and stramenopiles to some alveolates.

### 3.2. Molecular Dating Analyses

In total, we calculated 18 chronograms, 6 for each of the three calibration sets: C1, C2 and C3 (Table 1 and Table S7). C3 represents our most up-to-date calibration set since it includes microfossils of *R*. *chitrakootensis* [12] and *P*. *antiquus* [13]; although their exact phylogenetic positions remain debated, they are widely used as the oldest credible macrofossils of red and green algae, respectively. The other sets were used for comparative studies to investigate the impact of calibration constraints on molecular clocks (see below). The estimated ages are presented as ranges of mean dates from all six molecular clocks, UCLNR, IGR, TK02, CIR, LN and UGAM, unless stated otherwise. We discuss only selected results concerning the evolution of major photosynthetic lineages, though more datings are also included in Supplementary Information.

**Figure 2:**
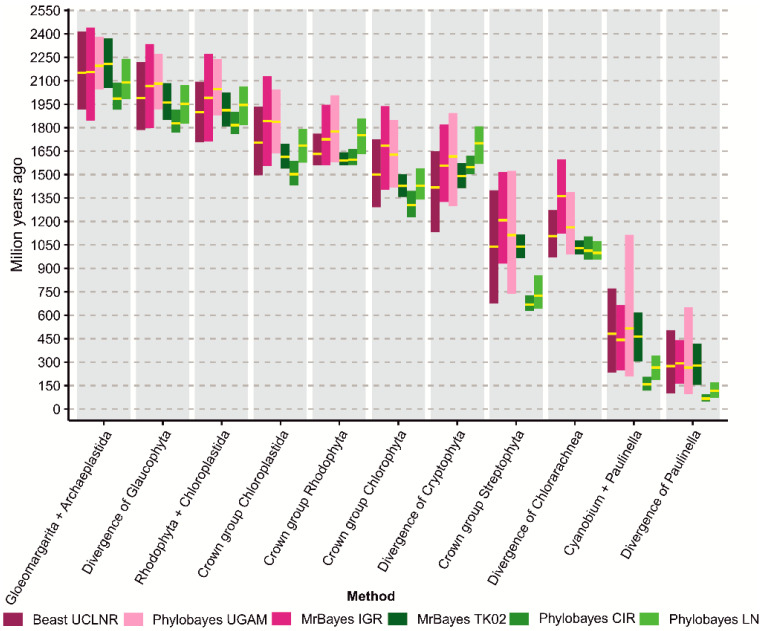
Comparison of molecular dating estimations for key evolutionary events for photosynthetic organisms discussed in the article. Purple and green colors mark uncorrelated and autocorrelated clocks, respectively. The yellow lines indicate the mean age and the bars 95% credibility intervals for the mean.

According to our chronograms based on the data set C3 (Table 1), the cyanobacterial ancestors of muroplasts, rhodoplasts and chloroplasts diverged from *G*. *lithophora* in the Paleoproterozoic Era between 2.2 and 2.0 Ga (Figure 1, Figure 2 and Figures S1–S5; Table S8). Before 2.1 to 1.8 Ga, i.e., prior to the divergence of the first Archaeplastida lineage, the plastid progenitors became involved in the endosymbiotic interaction that shaped the Earth’s biosphere. Our age for the first cyanobacterial endosymbiosis is in line with the chronograms calculated on nuclear markers by Strassert et al. [54], plastid markers by Blank [56] and Sánchez-Baracaldo et al. [30], but much older than the other estimations summarized in Table S1.

Following the emergence of glaucophytes, red and green algae diverged between 2.0 and 1.8 Ga (Figure 1, Figure 2 and Figures S1–S5; Table S8). Their first species were unicellular algae, but the multicellularity in Archaeplastida could have evolved as early as 1.9 Ga and surely before 1.6 Ga. The first date represents the age of the controversial fossil of *Grypania*, possibly an early red or green multicellular alga [57], and the latter *R*. *chitrakootensis* [12]. According to our clocks, the crown groups of Chloroplastida and Rhodophyta were both established in the late Paleoproterozoic Era, between 1.8 and 1.6 Ga; only the CIR model inferred ~1.5 billion years (Gyr) for Chloroplastida. This time range is much older than most previous estimations (Table S1), indicating the Mesoproterozoic and, for extant Chloroplastida, also the Neoproterozoic Era as their periods of origin (Figure 1, Figure 2 and Figures S1–S5; Table S8).

We estimated the age for the crown group of Chlorophyta between 1.7 and 1.3 Ga (Figure 1, Figure 2 and Figures S1–S5; Table S8), and it was much older than the other calculations though similar to Blank’s [56] results (Table S1). Interestingly, the extant streptophytes seem to be younger according to our results compared with the most recent reports (Table S1). We inferred that they evolved in the late Mesoproterozoic, between 1.2 and 1.0 Ga (Figure 1, Figure 2, Figures S1, S2 and S5; Table S8). However, this time range does not take into account the mean dates calculated by CIR and LN clocks (~700 Myr), which strongly differed from our other models (Figures S3 and S4).

Single-celled green algae belonging to Chlorophyta, class Ulvophyceae, became involved in an endosymbiotic interaction with a cercozoan amoeba, resulting in a new photosynthetic lineage of Chlorarachnea [53]. According to our studies, their complex plastid split from their ancestral green algae between 1.1 and 1.0 Ga (Figure 1, Figure 2 and Figures S2–S5; Table S8); only the IGR model (Figure S1) indicated an earlier mean age (1.36 Byr). These results are consistent with the nuclear marker-based clock by Parfrey et al. [58], dating this event to about 1.0 Ga, but not with estimations based on plastid proteins by Jackson et al. [53], assuming the mean date ~600 Ma.

Contrary to chlorarachniophytes, the commonly accepted red alga acquisition by cryptophytes triggered plastid transfers to other protist lineages in tertiary or indirectly in higher order endosymbioses, e.g., to stramenopiles, haptophytes and dinoflagellates [54]. Our results indicate that the process started between 1.7 and 1.4 Ga (Figure 1, Figure 2 and Figures S1–S5; Table S8), which is similar to Strassert et al. [54] estimation. Importantly, Pietluch et al. [55] assume two independent secondary red-alga endosymbioses, one within stramenopiles between ~1.67 and ~1.52 Ga and another within cryptophytes between ~1.53 and ~1.18 Ga.

The minimum age for the second cyanobacterial endosymbiosis amounting to about 60 Ma was first proposed by Nowack et al. [8]. They assumed that the rate of pseudogene disintegration requiring from 40 to 60 Myr in the endosymbiotic bacteria *Buchnera aphidicola* is comparable to that of *Paulinella* in chromatophores. Subsequent molecular dating studies based on 18S rRNA with the UCLNR model by Delaye et al. [59] and also Lhee et al. [60] estimated that photosynthetic *Paulinella* species diverged from their heterotrophic relatives 141-94 Ma and 193-64 Ma, respectively. In turn, Sánchez-Baracaldo et al. [30] based on plastid markers, calculated the split of *P*. *chromatophora* from the *Synechococcus*/*Cyanobium* clade between 634 and 350 Ma.

We are the first to present multiple molecular clock models applied in studies that include all known *Paulinella* photosynthetic species. According to our four clocks, cyanobacterial ancestors of chromatophores diverged from *C*. *gracile* in the early Paleozoic Era between 516 and 443 Ma (Figure 1, Figure 2, Figures S1, S2 and S5; Table S8). These results are in line with Sánchez-Baracaldo et al. [30] estimations, but we managed to narrow the upper and lower limits of the time range to about 100 Myr each. The second cyanobacterial endosymbiosis must have taken place before 292 to 266 Ma, i.e., before *P*. *chromatophora* diverged from the other photosynthetic *Paulinella* species. It would mean that this endosymbiosis is much older than the other authors assumed. However, these time ranges do not include mean dates calculated by the LN and CIR clocks, which strongly differ from those produced by our other models. LN and CIR chronograms indicated that *Paulinella* split from *C*. *gracile* between 266 and 158 Ma, respectively, and therefore acquired plastids between 118 and 67 Ma (Figure 2, Figures S3 and S4; Table S8). In turn, these ages are in better agreement with the results of Nowack et al. [8], Delaye et al. [59] and Lhee et al. [60].

### 3.3. The Impact of Molecular Clocks and Calibration Sets on Age Estimations

In order to evaluate the discrepancies in age estimations by different approaches, we compared (i) different calibration sets assuming the same molecular clock and (ii) a given calibration set with different molecular clocks. The first comparison indicated that the data set C3 (Table 1) always provided older ages than the global mean for all three calibration sets. This was expected, considering that C3 included the 500 Myr-older calibration point for the earliest Rhodophyta and 300 Myr-older for Ulvophyceae compared to the other sets. These fossil constraints have a strong impact on inferred divergence times, pushing estimates further back in time. The average percentage difference from the global mean per node D (see equation in Section 2) for C3 depended on the clock model and ranged from about 1% to 7% (Table 2). The ages estimated based on C1 were mostly younger than the global mean, from about 1% to 9%, and those based on C2 were in the middle in this respect (Table 1 and Table 2).

We also visualized these tendencies in Figure S21 as pairwise comparisons of the calibration sets, and the differences between them were statistically significant (*p* < 2 × 10^−45^). The largest average pairwise differences in ages per node P (see equation in Materials and Methods) equal to 135 and 121 Myr were observed for the comparison of C1 with C3 for clocks TK02 and LN, respectively, and they corresponded to the average percentage pairwise difference per node P% (see equation in Section 2) 17% and 14%. It is also worth mentioning that molecular dating by the UGAM model was least affected by the change in the calibration set (Table 2).

Much greater differences could be noticed for the second comparison, i.e., of different molecular clocks with the same calibration set (Table 2). For each calibration set, the LN model estimated on average from 13% to 20% older ages than the mean calculated for all the six chronograms. Quite old ages were also obtained for the IGR clock, from 6.5% to 13% greater than the global mean. The younger ages were produced by UCLNR, and they were about 18% smaller than the global mean. UGAM and CIR also provided younger ages, but the results of TK02 depended on the calibration set. This suggests that divergence time estimates are highly sensitive to the underlying assumptions of the molecular clock model, with LN and IGR producing consistently older estimates and UCLNR the youngest.

The pairwise comparison between the clocks is presented in Figure S22, and the differences in the estimated ages are statistically significant for the comparison of LN and UCLNR with the other five models (*p* < 6.5 × 10^−7^), for the comparisons of UGAM-CIR (*p* = 0.041) and UGAM-IGR (*p* = 0.019). As expected from the global comparison, we found the biggest average pairwise difference in ages per node P between the LN and UCLNR clocks. The ages were, on average, 208, 273 and 282 Myr older for LN than UCLNR for C1, C2 and C3 sets, respectively, which corresponds to the mean percentage pairwise difference per node P% 25%, 33% and 31%.

**Figure 3:**
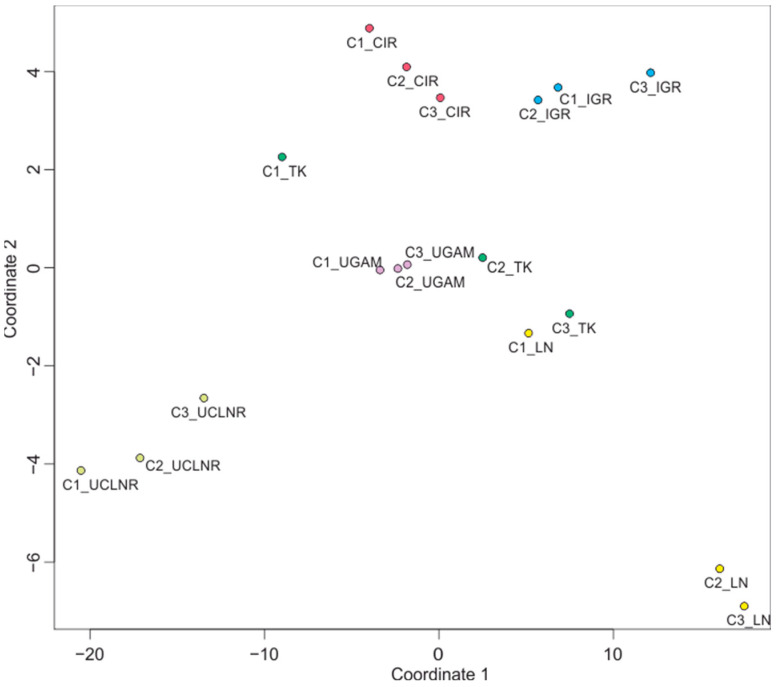
Visualization of similarities and differences in age estimation for all combinations of calibration sets and molecular clocks in multidimensional scaling. The three calibration sets with the UCLNR clock (light green dots) and the set C2 and C3 with the LN clock (yellow dots) represent the most distant points because they calculated the most extreme age estimations in our analyses, the youngest and the oldest, respectively. The other combinations of clocks and sets, depicted as dots of appropriate color, are located between them since they provided more moderate datings.

We also performed multidimensional scaling to visualize the similarities and differences indicated above for all the combinations of calibration sets and molecular clocks. This analysis illustrates that there are clearly greater differences in the age estimations between molecular clocks compared to calibration sets. In most cases, the calibration sets were grouped together independently of the clock model applied, but the clocks were generally scattered across the plot (Figure 3). This further reinforces that the choice of molecular clock model exerts a stronger influence on divergence time estimates than calibration set selection. Thus, comparing multiple models remains essential to accurately assess uncertainty in molecular dating.

## 4. Conclusions

The age estimations for our key evolutionary events concerning photosynthetic organelles were generally older compared to previous reports (Table S1). We indicate that (i) primary plastids evolved prior to 2.1–1.8 Ba, which represents the divergence of glaucophytes from the other Archaeplastidans; (ii) *Paulinella* chromatophores originated most probably before 292–266 Ma; and (iii) red and green algae were engulfed by cryptophyte and chlorarachniophyte ancestors between 1.7–1.4 Ba, and 1.1–1.0 Ba, respectively.

Our results favor the glaucophyte-first hypothesis and agree with the greatest number of cyanobacterial characteristics preserved in muroplasts compared with rhodoplasts and chloroplasts, e.g., the presence of peptidoglycan and carboxysomes [10]. However, morphological conservatism does not necessarily mean that Glaucophyta diverged first because the other Archaeplastida lineages could have simply lost certain ancestral traits independently. In accordance with this, previous phylogenetic analyses based on plastid markers back all the possible evolutionary models. Considering the number of supporting threes in descending order, they are Glaucophyta-, Chloroplastida- or Rhodophyta-first hypotheses [3,10,61,62]. Interestingly, the trees built on nuclear markers that do support the monophyly of Archaeplastida prefer either the Glaucophyta or Rhodophyta as the first branching lineage [10,11,54,63,64,65,66]. There is also generally greater support for the former scenario [10,66], but, on the other hand, the most recent studies seem to favor the latter [11,54,63,64,65]. Taken together, our findings contribute to this ongoing debate but do not resolve it definitively.

The analyses of the impact of molecular clocks and calibration sets on the age estimations indicate that there are clearly greater differences in the ages between the clocks compared to the calibration sets (Table 2). The largest mean difference in the age estimation between an autocorrelated LN and uncorrelated UCLNR model amounting to 208, 273 and 282 Myr for C1, C2 and C3 sets might suggest that there is a dependence between autocorrelated/uncorrelated models and the older/younger age estimation, respectively. Although some models seem to follow this rule (e.g., UGAM), others do not (e.g., IGR). This indicates that the relationship is not true. The age estimation is more complex and depends on many factors, including estimated model parameters, the specificity of data sets and calibration constraints.

## Figures and Tables

**Table 1:** Calibration constraints for dating plastid evolution. A detailed description of each calibration point is provided in Table S7. C1, C2 and C3 represent the first, second and third calibration set, respectively.

Node No.	Node Name	Min.			Max.			Ref.	
		C1	C2	C3	C1	C2	C3	Min.	Max.
Cyanobacteria									
1	Great Oxidation Event	2320	2320	2320	3000	3000	3000	[32]	[33]
2	Earliest cyanobacteria	1900	1900	1900	3000	3000	3000	[34]	[33]
3	Pleurocapsales	1700	1640	1640	1900	3000	3000	[35]	[33,34]
4	Nostocales	1600	1580	1580	1900	3000	3000	[36,37]	[33,34]
5	*Richelia*	110	110	110	3000	3000	3000	[38]	[33]
Rhodoplast									
6	Earliest Rhodophyta	1050	1030	1560	3000	2300	2300	[12,31]	[33,39]
7	Floridiophyceae	600	595	595	3000	2300	2300	[40]	[33,39]
Rhodophyta-derived plastid									
8	Earliest diatom	190	185	185	3000	2300	2300	[41]	[33,39]
9	Bacillariophytina	110	110	110	3000	2300	2300	[38]	[33,39]
Chloroplast									
10	Ulvophyceae	635	948	948	3000	2300	2300	[13]	[33,39]
11	Earliest land plants	475	471	471	501	515	515	[42]	[30,43]
12	Tracheophyta	446	446	446	501	515	515	[44]	[30,43]
13	Angiosperms/Gymnosperms	385	385	385	501	515	515	[45]	[30,43]
14	Angiosperms	130	130	130	501	515	515	[46,47]	[30,43]
15	Eudicots	125	125	125	501	515	515	[48]	[30,43]
16	Zygnemataceae	345	345	345	3000	2300	2300	[49]	[33,39]
17	Gymnosperms	385	385	385	501	515	515	[45]	[30,43]

**Table 2:** The average percentage difference from the global mean per node D calculated for the three calibration sets assuming the same molecular clock model (A) and for six molecular clocks assuming the same calibration set (B). The values should be compared in rows for A and in columns for B. UC—states for uncorrelated clock; AC—for autocorrelated clock.

	A: Impact of Calibration Sets on Dating for a Given Clock			B: Impact of Molecular Clocks on Dating for a Given Calibration Set		
	Calibration Set			Calibration Set		
Clock	C1	C2	C3	C1	C2	C3
Beast UCLNR (UC)	−3.57	−0.33	3.9	−17.93	−18.58	−18.05
MrBayes IGR (UC)	−1.3	−2.47	3.77	13.16	6.51	9.79
MrBayes TK02 (AC)	−9.3	1.9	7.4	−5.61	0.49	2.21
PhyloBayes CIR (AC)	−2.06	0.05	2.01	−0.15	−3.19	−4.29
PhyloBayes LN (AC)	−8.32	3.24	5.08	13.03	20.96	19.14
PhyloBayes UGAM (UC)	−0.84	0.14	0.69	−2.5	−6.19	−8.8

## Data Availability

Data available from the Dryad Digital Repository: https://doi.org/10.5061/dryad.70rxwdc1g.

## Supplementary Materials

The following supporting information can be downloaded at https://www.mdpi.com/article/10.3390/ijms26125569/s1.
