# Supplementary material for: Dating the Origin and Spread of Plastids and Chromatophores
Source: Int J Mol Sci. 2025 Jun 11;26(12):5569. doi: 10.3390/ijms26125569 (PMC12193072; doi:10.3390/ijms26125569)
Supplement: Supplementary file 1 [file ijms-26-05569-s001.zip › ijms-3660457-supplementary.pdf]

This file includes:

Supplementary text

Figures S1 to S22

Tables S1 to S8

SI References

SUPPLEMENTARY TEXT

Verification of tentatively assigned microfossils

In the light of the obtained chronograms based on the calibration set C3, we were able to verify taxonomic affiliation of tentatively and controversially assigned fossils. Our estimations indicate that the main extant cyanobacterial lineages diverged between 2.5 and 1.6 Ga (Fig. 1, S1, S3-S5); only TK02 model indicated an older mean dating (2.74 Ga) (Fig. S2). Consequently, the assignment of individual Archaean microfossils to contemporary classes or orders of cyanobacteria is not substantiated based on our results. Actually, many of these specimens are the subject of hot debate because they may represent other bacteria groups or are considered not to be organismal remains at all (Wacey et al. 2016). Our chronograms support the assignment of *Oscillatoriopsis* (2.2 - 1.8 Ga, Australia (Knoll et al. 1988)) but not *Siphonophycus* (2.5 - 2.3 Ga, South Africa (Klein et al. 1987)) to *Oscillatoriales* because *Oscillatoriales* diverged between 2.1 and 1.8 Ga (Fig. 1, S1-S5). Moreover, *Eoentophysalis belcherensis*, the oldest microfossil interpreted with certainty as a cyanobacterium (~1.9 Ga, Canada), does not represent the contemporary lineage of *Chroococcales*; according to our results this order together with other coccoidal cyanobacteria (*Chroococcidiopsidales*) evolved between 1.8 and 1.6 Ga (Hofmann 1976a) (Fig. 1, S1-S5).

An interesting but controversial fossil represents *Grypania*, with the oldest occurrence described from 1.9 Byr-old formations in Michigan, USA (Han and Runnegar 1992; Pietrzak-Renaud and Davis 2014). *Grypania* has been commonly interpreted as a macroalga but alternatively named: a pseudofossil, trace fossil, cyanobacterium or even a metazoan (Wang et al. 2016). Its age falls well within the estimated time range for the split of the Archaeplastida lineages, i.e. between 2.1 and 1.8 Ga (Fig. 1, 2, S1-S5). Accordingly, *Grypania* might represent an early

green or red alga though it would mean that the multicellularity evolved rather early in the archaeplastidan evolution.

Bangiomorpha is another example of an interesting microfossil and until recently it has been considered to be the oldest red alga specimen (~1.0 Ga, Canada (Gibson et al. 2018)). Our chronograms indicate that it could be directly related to extant representatives of the order *Bangiales,* class *Bangiophyceae,* as morphological data suggest (Gibson et al. 2018). We estimated the age for this group between 1.4 and 1.0 Ga (Fig. S1-S5); only the Beast model indicated a younger mean dating (868 Ma, Fig. 1).

According to our analyses, some acritarchs, i.e. unicellular organic-walled microfossils of Proterozoic era, described as *Dictyosphaera-Shuiyousphaeridium*, *Gigantosphaeridium* and *Leiosphaeridia*, could belong to the Chloroplastida as interpreted by some authors e.g. Agić et al. (2017). Their oldest representatives were found in 1.7 - 1.4 Byr-old deposits in China (Agić et al. 2017), which is more or less at the time of the diversification of extant green algae in our chronograms (1.84 - 1.55 Ga) (Fig. 1, S2-S5). Moreover, our results also support the classification of some acritarchs to prasinophytes (Teyssedre 2007; Agić et al. 2017). Some of the oldest fossils of such type are *Simia* (1.7 - 1.4 Ma, China), *Valeria* (1.7 - 1.4 Ga, China), *Tasmanites* (~1.6 Ga, India) and *Pterospermella* (1.3 - 1.2 Ga, Greenland); their age corresponds well with the divergence of prasinophytes in our study between 1.7 and 1.2 Ga (Fig. 1, S2-S5).

We estimated the split of the clade consisting of *Trebouxiophyceae*, *Ulvophyceae* and *Chlorophyceae* from prasinophytes between 1.5 and 1.2 Ga (Fig. 1, S2-S5). Consequently, the assignment to this group of *Osculosphaera*, *Trachyhystrichosphaera* and *Vandalosphaeridium* dated to 1.25 Ga could be correct (Moczydłowska et al. 2011). However, *Tappania plana* (1.7 - 1.4 Ga, China) probably represents a fungus, especially taking into account that only two models:

IGR (Fig. S1) and UGAM (Fig. S5) indicated datings older than 1.4 Ga (Agić et al. 2017). *Leiosphaeridia gorda*, *Cerebrosphaera*, *Culcitulisphaera* and *Lanulatisphaera* presumably belong to *Chlorophyceae* (Moczydłowska et al. 2011; Loron and Moczydłowska 2018), and their oldest remains dated ~1.0 Ga from Canada and 800 - 750 Mya from Kazakhstan, Sweden and Spitsbergen do agree with the divergence of *Chlorophyceae* in our analyses estimated between 1.4 and 1.0 Ga (Fig. 1, S2-S5). The chronograms we obtained also support that *Archaeoclada*, *Variaclada* and *Eoprotoderma* described from ~1.0 Byr-old formations in Siberia (Hermann and Podkovyrov 2010; Moczydłowska et al. 2011) belong to the *Ulvophyceae* class that evolved between 1.27 and 1.0 Ga (Fig. 1, S2-S5). However, the classification of *Spiromorpha* (1.7 - 1.4 Ga, China) to *Zygnematophyceae* seems inconsistent with our estimations because our results indicate that *Zygnematophyceae* separated only between 941 and 551 Ma (Fig. 1, S2-S5).

We also calculated the divergence of land plants (embryophytes) and the bryophyte-tracheophyte clade to 508 - 514 Ma and 479 - 462 Ma, respectively; only IGR (831 Ma, 618 Ma) and TK02 (660 Ma, 569 Ma) models indicated older mean datings (Fig. 1, S2-S5). Our estimations allow us to assume that the ~510 Myr-old spores from Arizona and Tennessee in USA (Strother 2016a) and ~480-Myr-old spores from Australia (Strother and Foster 2021) could indeed represent the early land plants, and the ~460 Myr-old trilete spores from Sweden the vascular plants (Rubinstein and Vajda 2019).

Table S1. Comparison of molecular clock estimates from various studies for the divergence of the crown groups of Archaeplastida.

	Strassert et al. 2021		Nie et al. 2020		Sánchez-Baracaldo et al. 2017		Yang et al. 2016	Blank 2013		Parfrey et al. 2011	Berney at al. 2006	Yoon at al. 2004		Douzery et al. 2004	Hedges et al. 2004
**Crown Group**	**MIN**	**MAX**	**MIN**	**MAX**	**MIN**	**MAX**	**MEAN**	**MIN**	**MAX**	**MEAN**	**MEAN**	**MIN**	**MAX**	**MEAN**	**MEAN**
Archaeplastida	1807,29	2137,51	1644[bc]	1644[bc]	1900	1939	1693[c]	2006	2274	1556[b]	930	1535	1719	1017[bc]	1428[c]
Glaucophyta	681,61	1440,58	-	-	1695[a]	1781[a]	-	1729[a]	1952[a]	598[b]	-	1535[a]	1719[a]	-	-
Rhodophyta	1606,29	1657,63	1264[b]	1264[b]	1060	1161	1511[b]	1412	1504	1236[b]	740[b]	1349	1452	928[a]	1428[ac]
Chloroplastida	1064,73	1523,87	1162,9	1738,2	1254[b]	1254[b]	-	1597	1710	941[b]	695[b]	-	-	729	968
Chlorophyta	940,89	1376,12	1065,8	1428,3	1037	1092	-	1394	1599	851[b]	616[b]	-	-	729[a]	968[a]
Streptophyta	942,75	1394,45	1115,1	1575	880	999	482[b]	1438	1607	739[b]	604[b]	646	792	450[b]	707
**Markers**	320 nuclear-encoded proteins		81 chloroplast-encoded protein genes		26 plastid-encoded proteins		nuclear-encoded EF2 protein, LSU and SSU rRNAs, mitochondrial-encoded Cox1 protein, and plastid-encoded RbcL, PsaA and PsbA proteins	plastid-encoded LSU/SSU rRNA genes		15 nuclear-encoded protein genes	plastid-encoded SSU rRNA gene	plastid-encoded SSU rRNA, *psaA*, *psaB*, *psbA*, *rbcL*, and *tufA* genes and PsaA, PsaB, PsbA, RbcL, and TufA proteins		129 nuclear-encoded proteins	50-74 nuclear-encoded proteins

a – one species represents the crown group
b – age read from the tree
c – tree without any *Glaucophyta* representatives

Table S2. List of 30 conserved plastid-encoded proteins and query species used for homologous search by PSI-BLAST.

Gene	Protein	NCBI accession number				
		Arabidopsis thaliana	***Cyanobium sp.* NIES-981**	***Cyanophora paradoxa***	***Galdieria sulphuraria***	***Gloeomargarita lithophora* Alchichica-D10**
atpA	ATP synthase subunit alpha	ANW47776.1	SBO44543.1	NP_043222.1	AIG92565.1	APB33236.1
atpB	ATP synthase subunit beta	NP_051066.1	WP_087068740.1	NP_043241.1	AIG92549.1	APB34992.1
atpH	ATP synthase subunit c	ANW47777.1	SBO44542.1	NP_043226.1	AIG92561.1	WP_071453852.1
ccsA	Cytochrome c biogenesis protein	NP_051108.1	SBO42325.1	NP_043267.1	P31564.1	APB32776.1
petA	Component of the cytochrome b6-f complex	ANW47803.1	SBO42987.1	NP_043244.1	AIG92543.1	APB32505.1
petB	Component of the cytochrome b6-f complex	NP_051088.1	SBO43464.1	NP_043175.1	AIG92473.1	APB34594.1
petD	Component of the cytochrome b6-f complex	NP_051089.1	SBO43463.1	NP_043174.1	AIG92474.1	APB34593.1
psaA	Photosystem I P700 chlorophyll a apoprotein A1	NP_051059.1	SBO43593.1	AAA81181.1	AGZ04878.1	APB33122.1
psaC	Photosystem I iron-sulfur center	ANW47840.1	SBO43969.1	AAA81301.1	AIG92625.1	WP_071454602.1
psbA	Photosystem II protein D1	CAA56270.1	SBO44640.1	NP_043238.1	AIG92634.1	APB33505.1
psbB	Photosystem II CP47 reaction center protein	ANW47816.1	SBO43481.1	AAA81198.1	AIG92640.1	APB33253.1
psbC	Photosystem II CP43 reaction center protein	ANW47786.1	SBO44375.1	NP_043248.3	AIG92464.1	APB33217.1
psbD	Photosystem II D2 protein	ANW47785.1	SBO44818.1	NP_043247.1	AIG92463.1	APB33216.1
psbE	Cytochrome b559 subunit alpha	NP_051076.1	SBO43722.1	NP_043178.1	AIG92575.1	APB32556.1
psbK	Photosystem II reaction center protein K	ANW47774.1	SBO43700.1	AAA81280.1	AIG92530.1	WP_071454683.1
rbcL	Ribulose bisphosphate carboxylase large chain	AAB68400.1	SBO42577.1	NP_043240.1	AGZ04769.1	APB32635.1
rpl14	50S ribosomal protein L14, chloroplastic	ANW47826.1	WP_087068059.1	AAA63624.1	AIG92509.1	APB34322.1
rpL20	50S ribosomal protein L20, chloroplastic	NP_051082.1	SBO44063.1	NP_043162.1	AIG92539.1	APB32669.1
rpoA	DNA-directed RNA polymerase subunit alpha	NP_051090.1	SBO43574.1	NP_043256.1	AIG92499.1	APB34409.1
rpoB	DNA-directed RNA polymerase subunit beta	CAA74024.1	SBO44625.1	NP_043230.1	AIG92555.1	APB34630.1
rpoC1	DNA-directed RNA polymerase subunit beta'	NP_051050.1	WP_087068805.1	NP_043229.1	AIG92556.1	WP_071455042.1
rpoC2	DNA-directed RNA polymerase subunit beta"	NP_051049.1	WP_087068804.1	NP_043228.1	AIG92557.1	APB34628.1
S11	30S ribosomal protein S11, chloroplastic	NP_051091.1	SBO43573.1	NP_043257.1	AIG92500.1	APB34410.1
S12	30S ribosomal protein S12, chloroplastic	ALE59953.2	SBO43601.1	NP_043209.1	AIG92495.1	APB33533.1
S19	30S ribosomal protein S19, chloroplastic	NP_051098.1	SBO43555.1	NP_043198.1	AIG92514.1	APB34316.1
S2	30S ribosomal protein S2, chloroplastic	NP_051048.1	SBO42340.1	NP_043227.1	AIG92558.1	APB35091.1
S3	30S ribosomal protein S3, chloroplastic	NP_051096.1	WP_087069299.1	NP_043196.1	AIG92512.1	APB34318.1
S4	30S ribosomal protein S4, chloroplastic	ANW47792.1	WP_087067709.1	NP_043212.1	AIG92603.1	APB34495.1
S7	30S ribosomal protein S7, chloroplastic	NP_051118.1	SBO43602.1	NP_043208.1	AIG92494.1	APB33532.1
ycf3	Photosystem I assembly protein	BAA84386.1	SBO44825.1	AAA81185.1	Q08814.1	WP_071453871.1

Table S3. Partitions and substitution models proposed by ModelFinder (Kalyaanamoorthy et al. 2017) for IQ-TREE (Minh et al. 2020).

Model	Partition no.	Partition range
LG+F+R6	Subset1	5116-5236, 4484-4564 1-500 8530-8658
cpREV+R6	Subset2	501-960 8659-8779
mtZOA+F+R6	Subset3	961-1039 2824-3175 2727-2823 4133-4483 1829-1987 1988-2726 3684-4132
cpREV+F+R6	Subset4	4565-4626 1040-1294 1295-1606
LG+R5	Subset5	1607-1828 4627-5115 3176-3683
cpREV+R6	Subset6	5237-5350 9095-9302
WAG+F+R7	Subset7_Subset12	5351-5649 7228-8529
cpREV+R7	Subset8	5650-6623 6624-7227 8871-9094 9303-9501
cpREV+R5	Subset9	8780-8870 9502-9656, 9657-9823

Table S4. Partitions and substitution models proposed by Partition finder (Lanfear et al. 2017) for RAxML (Stamatakis 2014).

Model	Partition no.	Partition range
LG+F	Subset1	5116-5236 4484-4564 1-500
cpREV+F	Subset2	501-960
LG+F	Subset3	961-1039 2824-3175
cpREV+F	Subset4	4565-4626 1040-1294
LG+F	Subset5	1295-1606
LG	Subset6	1607-1828 4627-5115 3176-3683
LG+F	Subset7	2727-2823 4133-4483 1829-1987 1988-2726 3684-4132
cpREV	Subset8	5237-5350 9095-9302
cpREV	Subset9	5351-5649
cpREV	Subset10	5650-6623
cpREV	Subset11	6624-7227
WAG+F	Subset12	7228-8529
JTT	Subset13	8530-8658
cpREV	Subset14	8659-8779
LG	Subset15	8780-8870
cpREV	Subset16	8871-9094
LG	Subset17	9303-9501
cpREV	Subset18	9502-9656
cpREV	Subset19	9657-9823

Table S5. Partitions and substitution models proposed by Partition finder (Lanfear et al. 2017) for MrBayes (Ronquist et al. 2012).

Model	Partition no.	Partition range
WAG+I+G	Subset1	5116-5236 4484-4564 1-500
cpREV+I+G	Subset2	501-960
WAG+I+G	Subset3	961-1039 2824-3175
cpREV+I+G	Subset4	4565-4626 1040-1294
WAG+I+G	Subset5	1295-1606
WAG+I+G	Subset6	1607-1828 4627-5115 3176-3683
WAG+I+G	Subset7	2727-2823 4133-4483 1829-1987 1988-2726 3684-4132
cpREV+I+G	Subset8	5237-5350 9095-9302
cpREV+I+G	Subset9	5351-5649
cpREV+I+G	Subset10	5650-6623
cpREV+I+G	Subset11	6624-7227
WAG+I+G	Subset12	7228-8529
JTT+I+G	Subset13	8530-8658
cpREV+G	Subset14	8659-8779
WAG+I+G	Subset15	8780-8870
cpREV+I+G	Subset16	8871-9094
WAG+I+G	Subset17	9303-9501
cpREV+I+G	Subset18	9502-9656
cpREV+G	Subset19	9657-9823

Table S6. Partitions and substitution models proposed by Partition finder (Lanfear et al. 2017) for Beast (Bouckaert et al. 2014).

Model	Partition no.	Partition range
LG+I+G	Subset1	1-500
LG+I+G	Subset2	501-960
LG+I+G	Subset3	4133-4483 961-1039 2824-3175
cpREV+G	Subset4	1040-1294
LG+I+G	Subset5	1295-1606
LG+I+G	Subset6	1607-1828 1988-2726
LG+I+G	Subset7	3684-4132 1829-1987
LG+I+G	Subset8	2727-2823
LG+I+G	Subset9	4627-5115 3176-3683 4484-4564
cpREV+I+G	Subset10	5237-5350 4565-4626
LG+I+G	Subset11	5116-5236
cpREV+I+G	Subset12	5351-5649
cpREV+I+G	Subset13	5650-6623 9095-9302
cpREV+I+G	Subset14	6624-7227
cpREV+I+G	Subset15	7228-8529
JTT+I+G	Subset16	8530-8658
cpREV+G	Subset17	8659-8779
LG+I+G	Subset18	8780-8870
cpREV+I+G	Subset19	8871-9094
LG+I+G	Subset20	9303-9501
cpREV+I+G	Subset21	9502-9656
cpREV+G	Subset22	9657-9823

Table S7. Description of calibration constraints used for dating plastid evolution.

Age (Mya)	Type	Description	Ref(s).
3,000	Maximum	The oldest evidence for the presence of atmospheric oxygen capable of weathering rocks, based on oxidative chromium isotopes and other redox sensitive metals found in 3,000 Myr-old South African formations from the Pongola Supergroup (Nsuze palaeosol and Ijzermyn iron formation). This dating suggests that significant levels of atmospheric oxygen were present 600 million years before the Great Oxidation Event.	(Crowe et al. 2013)
2,320	Minimum	The analysis of sulfur isotopes in the marine sediments from the Transvaal Supergroup (Rooihoogte and Timeball Hill formations) indicates that the oxygen level remained very low until about 2,320 Mya. This date represents the first major stable increase in atmospheric oxygen (the Great Oxidation Event) and is linked to the cyanobacterial oxygenic photosynthesis.	(Bekker et al. 2004a)
2,300	Maximum	Molecular clock analyses indicate this date as the origin of mitochondria (2300 - 1800 Mya). It exactly coincides with the Great Oxidation Event; after the rise of oxygen level, mitochondria and organisms with more than two or three cell types are presumed to have evolved.	(Hedges et al. 2004)
1,900	Minimum	The microfossil of *Eoentophysalis belcherensis* is the oldest microfossil interpreted with certainty as a cyanobacterium. It was described from 1,900 Myr-old stromatolitic dolostones of the Belcher Supergroup (Kasegalik and McLeary formations) in Hudson Bay, Canada. *E. belcherensis* closely resembles extant cyanobacteria from the genus *Entophysalis*, order *Chroococcales*.	(Hofmann 1976b; Demoulin et al. 2019)
1,640/ 1,700	Minimum	The microfossil of *Eohyella* represents another indisputable microfossil of cyanobacteria. It was described from 1,640/1,700 Myr-old stromatolites of the Changcheng Group (Dahongyu formation) in the Pangjiapu iron mine, China. *Eohyella* closely resembles extant cyanobacteria from the genus *Eohyella*, order *Pleurocapsales*.	(Zhang 1988)
1,580/ 1,600	Minimum	The microfossil of *Archaeoellipsoides* was described in samples from McArthur Basin, Australia. The date 1,589±3 represents the minimum age for McArthur group (Upper Balbirini Dolomite formation). The date 1,600 Mya refers to calibration point from Sánchez-Baracaldo et al. (Sánchez-Baracaldo et al. 2017).	(Page et al. 2000; Tomitani et al. 2006)
1,560	Minimum	The microfossil of *Rafatazmia chitrakootensis* represents the first multicellular eukaryote of red alga origin. It was described from 1,650±89 Myr-old phosphatized stromatolitic microbialites of the Tirohan Dolomite, part of the Chitrakoot Formation (Semri Group), India.	(Bengtson et al. 2017)
1,030/ 1,050	Minimum	The microfossil of *Bangiomorpha pubescens* is indisputably recognized as a multicellular red alga. It was described from 1,047 +0.013/-0.017 Myr-old chert rocks of the Bylot	(Gibson et al. 2018)

Age (Mya)	Type	Description	Ref(s).
		Supergroup (Angmaat Formation) in Baffin Island, Canada. The date 1,050 Mya refers to the calibration point from Sánchez-Baracaldo et al. (Sánchez-Baracaldo et al. 2017). *B. pubescens* resembles red algae in the order *Bangiales*, class *Bangiophyceae*.	
948	Minimum	The microfossil of *Proterocladus antiquus* represents the first multicellular green alga. It was described from 1,056 - 947.8 Myr-old mudstones of the Xihe Group (Nanfen Formation), China. *P. antiquus* resembles extant algae in the order *Siphonocladales*, class *Ulvophyceae*.	(Tang et al. 2020)
635	Minimum	The date was used in molecular clock analyses by Verbruggen's et al. (Verbruggen et al. 2009) as a maximum age constraint for siphonous algae (class *Ulvophyceae*), and by Sánchez-Baracaldo et al. (Sánchez-Baracaldo et al. 2017) as a minimum age constraint for *Ulvophyceae*.	(Verbruggen et al. 2009)
595/600	Minimum	Fossils of multicellular red algae that resemble species in the order *Corallinales*, class *Florideophyceae*. They were described from 599±4 Myr-old material of the Doushantuo Formation, China. The date 600 Mya refers to the calibration point from Sánchez-Baracaldo et al. (Sánchez-Baracaldo et al. 2017).	(Xiao et al. 2004)
515	Maximum	Molecular clock analyses indicate the date from 514.8 to 473.5 Mya as the origin of land plants. Moreover, at that time cryptospores were described from Cambrian Stage 4 mudstones of the Conasauga Group (Rome Formation), USA.	(Strother 2016b; Morris et al. 2018)
501	Maximum	The date 501 Mya refers to the calibration point from Sánchez-Baracaldo et al. (Sánchez-Baracaldo et al. 2017).	(Sánchez-Baracaldo et al. 2017)
471/475	Minimum	Earliest known cryptospores described from 473 - 471 Myr-old samples of the Zanjon Formation, Argentina. The date 475 Mya refers to the calibration point from Sánchez-Baracaldo et al. (Sánchez-Baracaldo et al. 2017).	(Rubinstein et al. 2010)
446	Minimum	The microfossils of spores of vascular plants described from 446 Myr-old (Late Katian) material of the Qusaiba-1 corehole, Saudi Arabia.	(Steemans et al. 2009)
385	Minimum	The microfossils of megaspores described from upper Eifelian and lower to middle Givetian samples of the Miastko 1 borehole, Poland. The megaspores resemble archaeopteridalean megaspores and megaspores from Carboniferous gymnospers.	(Turnau et al. 2009)
345	Minimum	The microfossils of *Zygnemataceae* (zygospores) described from Tournaisian samples collected e.g. from Algeria, Russia and UK.	(Mullins and Servais 2008)
185/190	Minimum	The first widely recognized record of centric diatoms described from Toarcian samples of the Liassic Boll shales of Wurtenburg, Germany. The date 190 Mya refers to the calibration point from Sánchez-Baracaldo et al. (Sánchez-Baracaldo et al. 2017).	(Kooistra and Medlin 1996)
130	Minimum	The microfossils of angiosperm pollen grains described from Hauterivian material of the Warlingham borehole, UK.	(Hughes et al. 1991; Friis et al. 2006)
125	Minimum	The fossil record of angiosperms, e.g. *Nymphaeaceae* or *Ceratophyllum*, goes back to the early Cretaceous about 125 Mya.	(Soltis and Soltis 2004)

Age (Mya)	Type	Description	Ref(s).
110	Minimum	The first well-preserved deposit of diatoms described from Aptian–Albian core material (115 - 110 Myr-old) recovered from the Weddell Sea, Antarctica.	(Sims et al. 2006)

Table S8. Molecular clock estimates for key nodes in our analyses.

Node	Clade	Age in million years according to the calibration set			Software
		C1	C2	C3	
I	*Gloeomargarita* + Archaeplastida	2039.29 (2342.93-1755.29)	2040.2 (2383.37-1762.4)	2156.46 (2440.91-1843.44)	MrBayes IGR
		1926.77 (2045.7-1817.55)	2132.25 (2311.24-1954.47)	2207.3 (2370.7-2053.59)	MrBayes TK02
		1929.82 (2172.05-1589.29)	2044.85 (2362.47-1712.71)	2150.93 (2416.38-1916.71)	Beast UCLNR
		2172 (2343.28-2016.44)	2189 (2374.48-2029.18)	2196 (2382.01-2044.88)	PhyloBayes UGAM
		1937 (2016.44-1853.27)	1947 (2047.2-1858.44)	1986 (2088.69-1914.05)	PhyloBayes CIR
		1951 (2040.66-1848.74)	2066 (2206.72-1947.44)	2090 (2240.55-1981.71)	PhyloBayes LN
II	Divergence of *Glaucophyta*	1943.82 (2232.79-1674.82)	1938.39 (2271.99-1644.06)	2066.61 (2335.76-1797.55)	MrBayes IGR
		1673.76 (1783.11-1569.51)	1884.4 (2027.79-1746.16)	1961.93 (2084.77-1849.59)	MrBayes TK02
		1765.99 (2015.53-1528.39)	1879.78 (2172.08-1568.27)	1989.09 (2218.78-1785.02)	Beast UCLNR
		2057 (2237.87-1883.46)	2075 (2263.76-1901.99)	2082 (2271.52-1917.57)	PhyloBayes UGAM
		1760 (1847.86-1662.12)	1780 (1880.16-1692.45)	1827 (1915.73-1768.82)	PhyloBayes CIR
		1772 (1869.1-1641.83)	1929 (2063.08-1814.06)	1953 (2074.21-1827.03)	PhyloBayes LN
III	*Rhodophyta* + Chloroplastida	1865.54 (2159.6-1615.89)	1860.63 (2199.38-1582.97)	1991.06 (2272.93-1710.75)	MrBayes IGR
		1617.86 (1727.17-1512.74)	1830.81 (1966.04-1703.3)	1911.82 (2024.92-1808.12)	MrBayes TK02
		1671.79 (1930.5-1429.17)	1786.14 (2043.62-1445.63)	1899.1 (2093.84-1708.03)	Beast UCLNR
		2023 (2206.7-1845.08)	2041 (2232.54-1863.43)	2047 (2238.14-1879.27)	PhyloBayes UGAM
		1748 (1838.96-1648.34)	1768 (1869.15-1679.89)	1816 (1902.12-1759.08)	PhyloBayes CIR
		1761 (1860.91-1625.52)	1921 (2053.56-1806.78)	1945 (2064.79-1816.96)	PhyloBayes LN
IV	Crown group Chloroplastida	1734.31 (1980.26-1494.98)	1738.38 (2045.77-1465.15)	1842.62 (2128.78-1555.16)	MrBayes IGR
		1333.18 (1434.37-1235.94)	1542.71 (1636.32-1455.86)	1613.12 (1696.34-1538.08)	MrBayes TK02
		1467.81 (1746.76-1237.7)	1619.35 (1860.73-1354.01)	1704.88 (1935.74-1495.19)	Beast UCLNR
		1812 (2019.18-1599.16)	1832 (2039.2-1628.45)	1837 (2044.86-1637.09)	PhyloBayes UGAM
		1397 (1528.58-1255.47)	1454 (1545.17-1374.24)	1501 (1586.33-1427.86)	PhyloBayes CIR
		1403 (1582.37-1206.65)	1672 (1802.9-1565.96)	1685 (1791.27-1576.43)	PhyloBayes LN
V	Crown group *Rhodophyta*	1561.2 (1833.6-1249.82)	1556.62 (1877.42-1264.31)	1725.66 (1946.86-1560)	MrBayes IGR
		1243.13 (1381.37-1083.67)	1438.21 (1590.67-1257.29)	1589.55 (1642.73-1560)	MrBayes TK02
		1366.18 (1669.52-1040.01)	1345.37 (1633.12-1081.83)	1631.66 (1761.57-1560)	Beast UCLNR
		1734 (1983.99-1436.78)	1748 (2002.26-1454.58)	1775 (2008.19-1579.91)	PhyloBayes UGAM
		1495 (1608.57-1365.58)	1528 (1641.91-1419.17)	1594 (1663.33-1561.13)	PhyloBayes CIR
		1525 (1660.16-1358.63)	1726 (1868.43-1581.22)	1753 (1859.33-1631.2)	PhyloBayes LN
VI	Crown group *Chlorophyta*	1573.77 (1798.44-1338.25)	1563.82 (1854.79-1323.15)	1684.43 (1937.8-1403.17)	MrBayes IGR
		1182.84 (1270.39-1096.79)	1378.07 (1454.4-1306.17)	1427.42 (1503.76-1356.86)	MrBayes TK02
		1251.99 (1499.09-1023.75)	1428.38 (1656.37-1201.08)	1500.98 (1726.29-1290.89)	Beast UCLNR
		1601 (1828.34-1368.07)	1624 (1846.25-1414.53)	1627 (1850.09-1417.64)	PhyloBayes UGAM
		1204 (1343.19-1063.81)	1274 (1353.18-1211.39)	1304 (1395.83-1227.31)	PhyloBayes CIR
		1129 (1336.33-986.17)	1425 (1528.42-1342.52)	1429 (1539.14-1338.81)	PhyloBayes LN
VII	Divergence of *Cryptophyta*	1407.77 (1713.89-1129.5)	1396.14 (1710.32-1085.27)	1557.89 (1820.99-1324.36)	MrBayes IGR
		1140.74 (1304.45-963.74)	1315.8 (1511.17-1084.34)	1490.19 (1572.41-1413.96)	MrBayes TK02
		1223.85 (1585.08-886.72)	1183.98 (1535.52-914.64)	1417.57 (1649.53-1132.57)	Beast UCLNR
		1578 (1870.16-1232.57)	1593 (1888.23-1247.86)	1616 (1893.52-1296.78)	PhyloBayes UGAM

Node	Clade	Age in million years according to the calibration set			Software
		**C1**	**C2**	**C3**	
		1446 (1564.29-1306.6)	1477 (1595.15-1356.91)	1547 (1619.29-1500.88)	PhyloBayes CIR
		1475 (1618.11-1299.68)	1672 (1824.28-1508.65)	1701 (1809.17-1567.2)	PhyloBayes LN
VIII	Crown group *Streptophyta*	1066.49 (1386.76-762.54)	1111.18 (1407.31-806.47)	1207.99 (1517.29-929.74)	MrBayes IGR
		893.29 (954.49-832.3)	1017.32 (1088.87-946.38)	1039.51 (1115.72-964.75)	MrBayes TK02
		934.62 (1252.09-628.56)	977.08 (1285.09-653.5)	1038.51 (1399.84-676.33)	Beast UCLNR
		1100 (1513.05-721.91)	1109 (1521.87-734.58)	1114 (1525.37-738.7)	PhyloBayes UGAM
		647 (703.68-607.41)	670 (722.44-629.42)	669 (725.81-628)	PhyloBayes CIR
		651 (731-597.1)	727 (862.59-640)	725 (856.23-641.43)	PhyloBayes LN
IX	Divergence of *Chlorarachnea*	1278.52 (1496.93-1065.85)	1261.96 (1500.83-1011.07)	1361.38 (1596.18-1123.31)	MrBayes IGR
		786.3 (872.12-694.81)	1013.77 (1049.55-983.86)	1029.02 (1078.44-989.03)	MrBayes TK02
		865.33 (1043.84-672.93)	1076.52 (1219.55-960.21)	1106.88 (1272.62-969.12)	Beast UCLNR
		1132 (1377.85-900.72)	1164 (1389.28-990.69)	1164 (1389.13-989.93)	PhyloBayes UGAM
		914 (1048.77-780.98)	991 (1058.92-954.61)	1013 (1104.08-957.52)	PhyloBayes CIR
		741 (928-649)	995 (1059.57-954.78)	999 (1073.15-955.83)	PhyloBayes LN
X	Cyanobium + *Paulinella*	450.67 (658.98-283.24)	416.01 (594.64-218.22)	442.6 (667.69-248.5)	MrBayes IGR
		425.31 (592.21-291.33)	442.37 (611.57-286.99)	463.08 (617.13-306.18)	MrBayes TK02
		424.81 (692.39-190.55)	434.21 (715.68-189.82)	481.24 (772.09-234.4)	Beast UCLNR
		511 (1109.22-203.27)	512 (1102.67-207.69)	516 (1115.18-207.52)	PhyloBayes UGAM
		156 (203.16-116.82)	159 (208.12-119.13)	158 (208.87-117.32)	PhyloBayes CIR
		240 (308.37-166.53)	259 (335.02-177.78)	266 (344.18-183.87)	PhyloBayes LN
XI	Divergence of *Paulinella*	298.39 (456.31-157.48)	278.82 (436-140.53)	291.97 (440.69-162.8)	MrBayes IGR
		259.74 (417.13-145.19)	263.91 (433.97-136.33)	279.23 (419.01-154.71)	MrBayes TK02
		231.31 (386.95-107.74)	239.99 (419.71-85.72)	275.32 (505.49-99.24)	Beast UCLNR
		264 (656.37-95.44)	264 (645.15-96.98)	266 (652.95-96.6)	PhyloBayes UGAM
		66 (92.45-46.33)	68 (94.82-47.36)	67 (94.31-46.18)	PhyloBayes CIR
		106 (149.5-63.99)	114 (163.42-70.23)	118 (170.22-71.86)	PhyloBayes LN

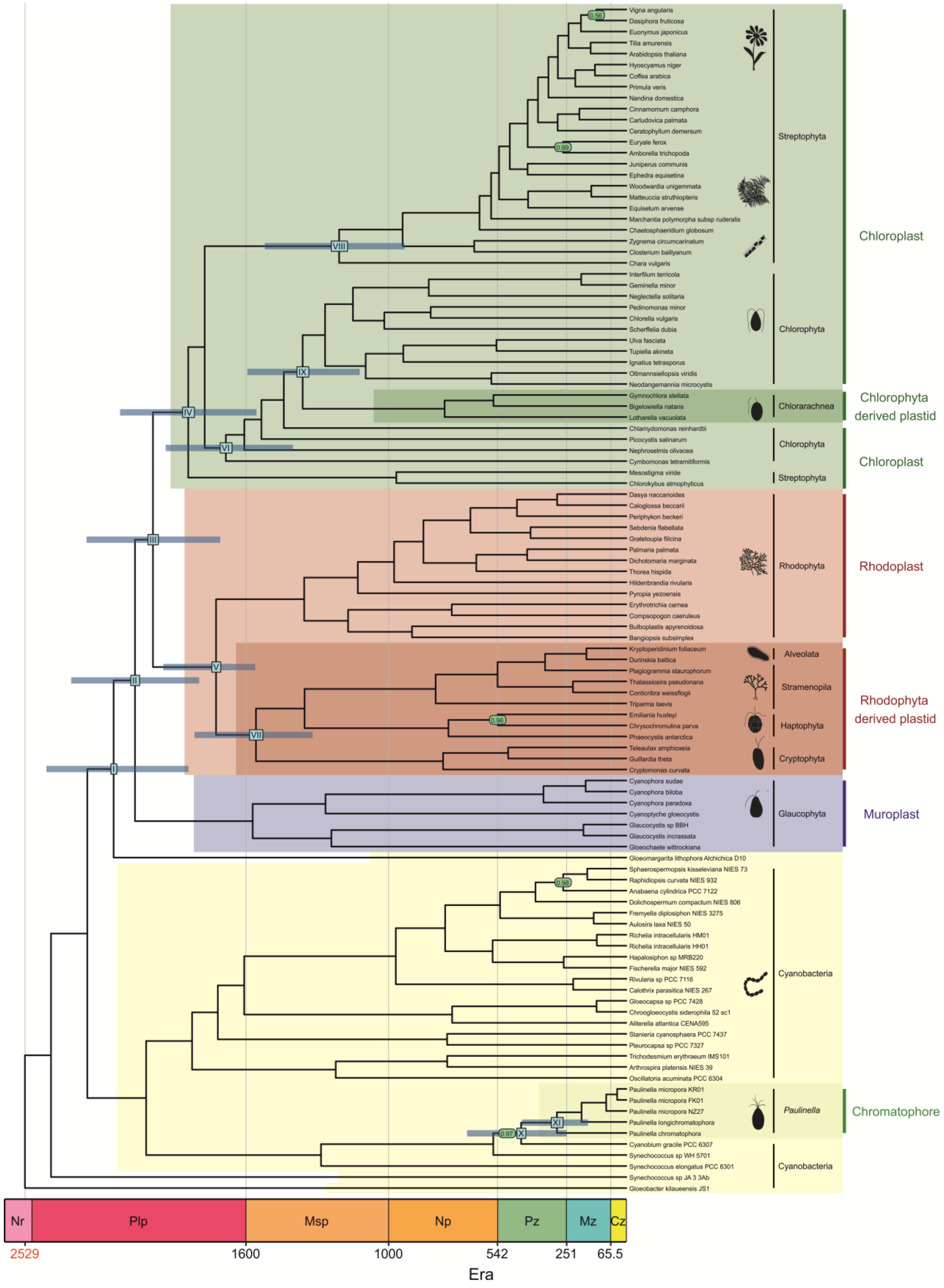

0.56
Vigna angularis
Dasiphora fruticosa
Euonymus japonicus
Tilia amurensis
Arabidopsis thaliana
Hyoscyamus niger
Coffea arabica
Primula veris
Nandina domestica
Cinnamomum camphora
Carludovica palmata
Ceratophyllum demersum
Euryale ferox
0.99
Amborella trichopoda
Juniperus communis
Ephedra equisetina
Woodwardia unigemmata
Matteuccia struthiopteris
Equisetum arvense
Marchantia polymorpha subsp ruderalis
Chaetosphaeridium globosum
VIII
Zygnema circumcarinatum
Closterium baillyanum
Chara vulgaris
Interfilum terricola
Geminella minor
Neglectella solitaria
Pedinomonas minor
Chlorella vulgaris
Scherffelia dubia
Ulva fasciata
Tupiella akineta
Ignatius tetrasporus
IX
Oltmannsiellopsis viridis
Neodangemannia microcystis
Gymnochlora stellata
IV
Bigelowiella natans
Lotharella vacuolata
Chlamydomonas reinhardtii
Picocystis salinarum
VI
Nephroselmis olivacea
Cymbomonas tetramitiformis
Mesostigma viride
Chlorokybus atmophyticus
Dasya naccarioides
Caloglossa beccarii
Periphykon beckeri
Sebdenia flabellata
III
Grateloupia filicina
Palmaria palmata
Dichotomaria marginata
Thorea hispida
Hildenbrandia rivularis
Pyropia yezoensis
Erythrotrichia carnea
Compsopogon caeruleus
Bulboplastis apyrenoidosa
Bangiopsis subsimplex
Kryptoperidinium foliaceum
Durinskia baltica
V
Plagiogramma staurophorum
II
Thalassiosira pseudonana
Conticribra weissflogii
Triparma laevis
Emiliania huxleyi
0.96
Chrysochromulina parva
VII
Phaeocystis antarctica
Teleaulax amphioxeia
Guillardia theta
I
Cryptomonas curvata
Cyanophora sudae
Cyanophora biloba
Cyanophora paradoxa
Cyanoptyche gloeocystis
Glaucocystis sp BBH
Glaucocystis incrassata
Gloeochaete wittrockiana
Gloeomargarita lithophora Alchichica D10
Sphaerospermopsis kisseleviana NIES 73
0.98
Raphidiopsis curvata NIES 932
Anabaena cylindrica PCC 7122
Dolichospermum compactum NIES 806
Fremyella diplosiphon NIES 3275
Aulosira laxa NIES 50
Richelia intracellularis HM01
Richelia intracellularis HH01
Hapalosiphon sp MRB220
Fischerella major NIES 592
Rivularia sp PCC 7116
Calothrix parasitica NIES 267
Gloeocapsa sp PCC 7428
Chroogloeocystis siderophila 52 sc1
Aliterella atlantica CENA595
Stanieria cyanosphaera PCC 7437
Pleurocapsa sp PCC 7327
Trichodesmium erythraeum IMS101
Arthrospira platensis NIES 39
Oscillatoria acuminata PCC 6304
Paulinella micropora KR01
Paulinella micropora FK01
Paulinella micropora NZ27
XI
Paulinella longichromatophora
0.97
X
Paulinella chromatophora
Cyanobium gracile PCC 6307
Synechococcus sp WH 5701
Synechococcus elongatus PCC 6301
Synechococcus sp JA 3 3Ab
Gloeobacter kilaueensis JS1
Streptophyta
Chloroplast
Chlorophyta
Chlorarachnea
Chlorophyta derived plastid
Chlorophyta
Chloroplast
Streptophyta
Rhodophyta
Rhodoplast
Alveolata
Stramenopila
Rhodophyta derived plastid
Haptophyta
Cryptophyta
Glaucophyta
Muroplast
Cyanobacteria
Paulinella
Chromatophore
Cyanobacteria
Nr
Plp
Msp
Np
Pz
Mz
Cz
2529
1600
1000
542
251
65.5
Era

(See figure on the previous page) **Figure S1.** Time-calibrated phylogeny of photosynthetic organelles and cyanobacteria. The tree was inferred with MrBayes under IGR model and calibrated with C3 (Tab. 1, S7). The roman numerals in blue rectangles mark key evolutionary events for plastids discussed in the article. At these nodes, there are blue bars representing 95% credibility intervals of the node age. The nodes supported with posterior probability lower than one are indicated in green circles.

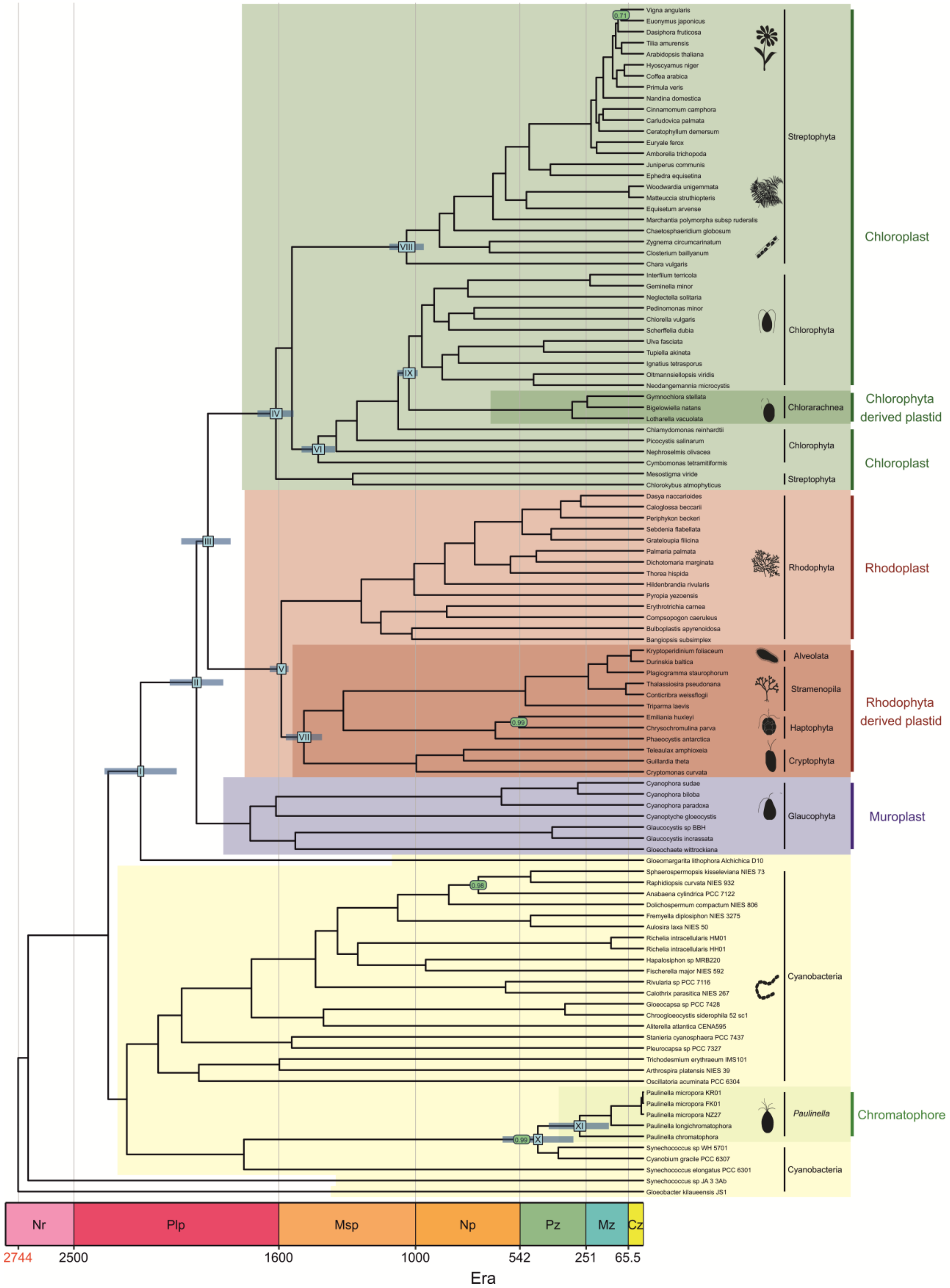

Vigna angularis
Euonymus japonicus
Dasiphora fruticosa
Tilia amurensis
Arabidopsis thaliana
Hyoscyamus niger
Coffea arabica
Primula veris
Nandina domestica
Cinnamomum camphora
Carludovica palmata
Ceratophyllum demersum
Euryale ferox
Amborella trichopoda
Juniperus communis
Ephedra equisetina
Woodwardia unigemmata
Matteuccia struthiopteris
Equisetum arvense
Marchantia polymorpha subsp ruderalis
Chaetosphaeridium globosum
Zygnema circumcarinatum
Closterium baillyanum
Chara vulgaris
Interfilum terricola
Geminella minor
Neglectella solitaria
Pedinomonas minor
Chlorella vulgaris
Scherffelia dubia
Ulva fasciata
Tupiella akineta
Ignatius tetrasporus
Oltmannsiellopsis viridis
Neodangemannia microcystis
Gymnochlora stellata
Bigelowiella natans
Lotharella vacuolata
Chlamydomonas reinhardtii
Picocystis salinarum
Nephroselmis olivacea
Cymbomonas tetramitiformis
Mesostigma viride
Chlorokybus atmophyticus
Dasya naccarioides
Caloglossa beccarii
Periphykon beckeri
Sebdenia flabellata
Grateloupia filicina
Palmaria palmata
Dichotomaria marginata
Thorea hispida
Hildenbrandia rivularis
Pyropia yezoensis
Erythrotrichia carnea
Compsopogon caeruleus
Bulboplastis apyrenoidosa
Bangiopsis subsimplex
Kryptoperidinium foliaceum
Durinskia baltica
Plagiogramma staurophorum
Thalassiosira pseudonana
Conticribra weissflogii
Triparma laevis
Emiliania huxleyi
Chrysochromulina parva
Phaeocystis antarctica
Teleaulax amphioxeia
Guillardia theta
Cryptomonas curvata
Cyanophora sudae
Cyanophora biloba
Cyanophora paradoxa
Cyanoptyche gloeocystis
Glaucocystis sp BBH
Glaucocystis incrassata
Gloeochaete wittrockiana
Gloeomargarita lithophora Alchichica D10
Sphaerospermopsis kisseleviana NIES 73
Raphidiopsis curvata NIES 932
Anabaena cylindrica PCC 7122
Dolichospermum compactum NIES 806
Fremyella diplosiphon NIES 3275
Aulosira laxa NIES 50
Richelia intracellularis HM01
Richelia intracellularis HH01
Hapalosiphon sp MRB220
Fischerella major NIES 592
Rivularia sp PCC 7116
Calothrix parasitica NIES 267
Gloeocapsa sp PCC 7428
Chroogloeocystis siderophila 52 sc1
Aliterella atlantica CENA595
Stanieria cyanosphaera PCC 7437
Pleurocapsa sp PCC 7327
Trichodesmium erythraeum IMS101
Arthrospira platensis NIES 39
Oscillatoria acuminata PCC 6304
Paulinella micropora KR01
Paulinella micropora FK01
Paulinella micropora NZ27
Paulinella longichromatophora
Paulinella chromatophora
Synechococcus sp WH 5701
Cyanobium gracile PCC 6307
Synechococcus elongatus PCC 6301
Synechococcus sp JA 3 3Ab
Gloeobacter kilaueensis JS1
0.71
0.99
0.98
0.99
I
II
III
IV
V
VI
VII
VIII
IX
X
XI
Streptophyta
Chlorophyta
Chlorarachnea
Chlorophyta
Streptophyta
Rhodophyta
Alveolata
Stramenopila
Haptophyta
Cryptophyta
Glaucophyta
Cyanobacteria
Paulinella
Cyanobacteria
Chloroplast
Chlorophyta derived plastid
Chloroplast
Rhodoplast
Rhodophyta derived plastid
Muroplast
Chromatophore
Nr
Plp
Msp
Np
Pz
Mz
Cz
2744
2500
1600
1000
542
251
65.5
Era

(See figure on the previous page) **Figure S2.** Time-calibrated phylogeny of photosynthetic organelles and cyanobacteria. The tree was inferred with MrBayes under TK02 model and calibrated with C3 (Tab. 1, S7). Other description as in Figure S1.

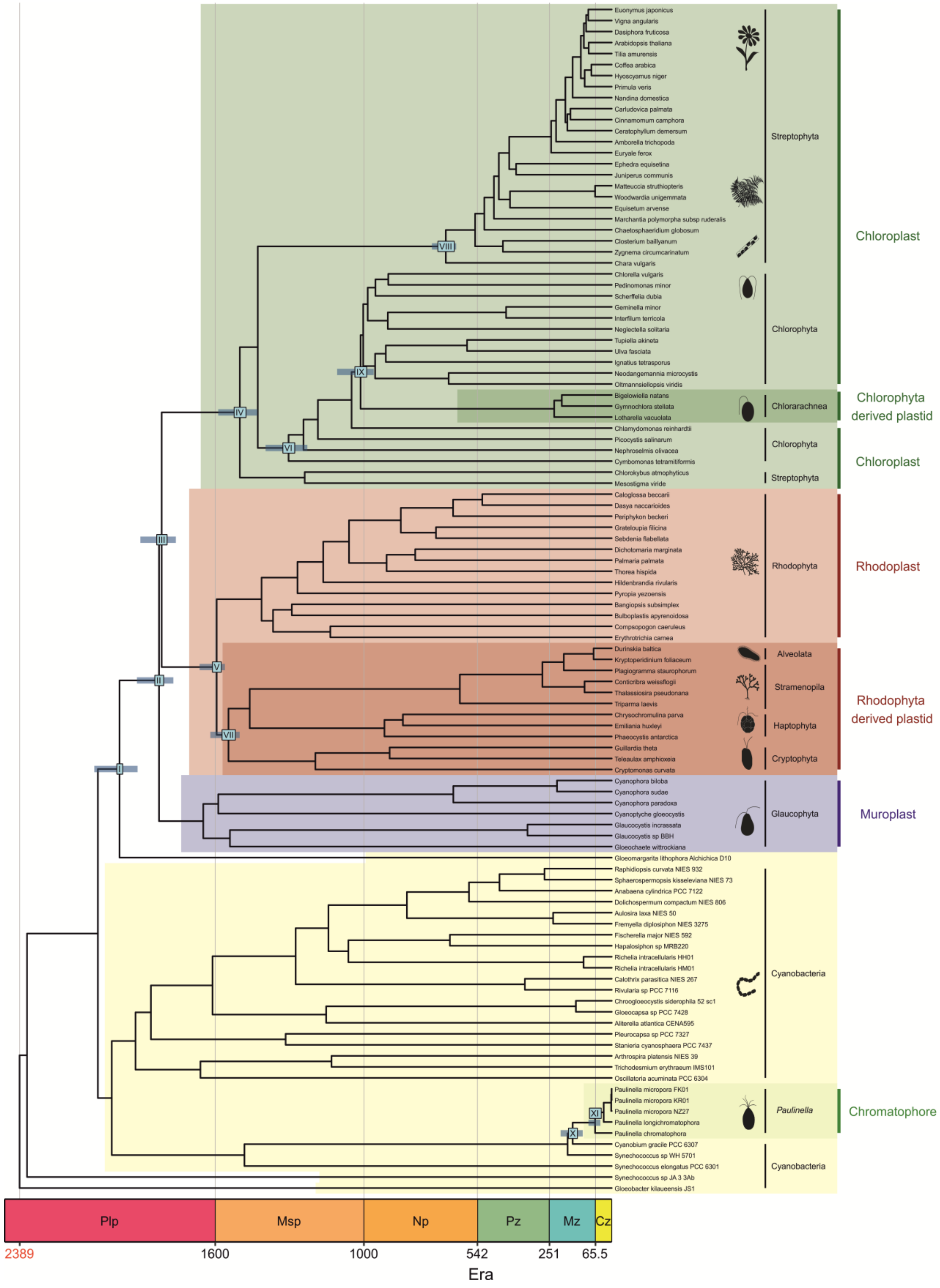
Euonymus japonicus
Vigna angularis
Dasiphora fruticosa
Arabidopsis thaliana
Tilia amurensis
Coffea arabica
Hyoscyamus niger
Primula veris
Nandina domestica
Carludovica palmata
Cinnamomum camphora
Ceratophyllum demersum
Amborella trichopoda
Euryale ferox
Ephedra equisetina
Juniperus communis
Matteuccia struthiopteris
Woodwardia unigemmata
Equisetum arvense
Marchantia polymorpha subsp ruderalis
Chaetosphaeridium globosum
Closterium baillyanum
Zygnema circumcarinatum
Chara vulgaris
Chlorella vulgaris
Pedinomonas minor
Scherffelia dubia
Geminella minor
Interfilum terricola
Neglectella solitaria
Tupiella akineta
Ulva fasciata
Ignatius tetrasporus
Neodangemannia microcystis
Oltmannsiellopsis viridis
Bigelowiella natans
Gymnochlora stellata
Lotharella vacuolata
Chlamydomonas reinhardtii
Picocystis salinarum
Nephroselmis olivacea
Cymbomonas tetramitiformis
Chlorokybus atmophyticus
Mesostigma viride
Caloglossa beccarii
Dasya naccarioides
Periphykon beckeri
Grateloupia filicina
Sebdenia flabellata
Dichotomaria marginata
Palmaria palmata
Thorea hispida
Hildenbrandia rivularis
Pyropia yezoensis
Bangiopsis subsimplex
Bulboplastis apyrenoidosa
Compsopogon caeruleus
Erythrotrichia carnea
Durinskia baltica
Kryptoperidinium foliaceum
Plagiogramma staurophorum
Conticribra weissflogii
Thalassiosira pseudonana
Triparma laevis
Chrysochromulina parva
Emiliania huxleyi
Phaeocystis antarctica
Guillardia theta
Teleaulax amphioxeia
Cryptomonas curvata
Cyanophora biloba
Cyanophora sudae
Cyanophora paradoxa
Cyanoptyche gloeocystis
Glaucocystis incrassata
Glaucocystis sp BBH
Gloeochaete wittrockiana
Gloeomargarita lithophora Alchichica D10
Raphidiopsis curvata NIES 932
Sphaerospermopsis kisseleviana NIES 73
Anabaena cylindrica PCC 7122
Dolichospermum compactum NIES 806
Aulosira laxa NIES 50
Fremyella diplosiphon NIES 3275
Fischerella major NIES 592
Hapalosiphon sp MRB220
Richelia intracellularis HH01
Richelia intracellularis HM01
Calothrix parasitica NIES 267
Rivularia sp PCC 7116
Chroogloeocystis siderophila 52 sc1
Gloeocapsa sp PCC 7428
Aliterella atlantica CENA595
Pleurocapsa sp PCC 7327
Stanieria cyanosphaera PCC 7437
Arthrospira platensis NIES 39
Trichodesmium erythraeum IMS101
Oscillatoria acuminata PCC 6304
Paulinella micropora FK01
Paulinella micropora KR01
Paulinella micropora NZ27
Paulinella longichromatophora
Paulinella chromatophora
Cyanobium gracile PCC 6307
Synechococcus sp WH 5701
Synechococcus elongatus PCC 6301
Synechococcus sp JA 3 3Ab
Gloeobacter kilaueensis JS1
Streptophyta
Chlorophyta
Chlorarachnea
Chlorophyta
Streptophyta
Rhodophyta
Alveolata
Stramenopila
Haptophyta
Cryptophyta
Glaucophyta
Cyanobacteria
Paulinella
Cyanobacteria
Chloroplast
Chlorophyta derived plastid
Chloroplast
Rhodoplast
Rhodophyta derived plastid
Muroplast
Chromatophore
I
II
III
IV
V
VI
VII
VIII
IX
X
XI
Plp
Msp
Np
Pz
Mz
Cz
2389
1600
1000
542
251
65.5
Era

(See figure on the previous page) **Figure S3.** Time-calibrated phylogeny of photosynthetic organelles and cyanobacteria. The divergence times were inferred with PhyloBayes under CIR model and calibrated with C3 (Tab. 1, S7). The tree topology was reconstructed in IQ-TREE. Other description as in Figure S1.

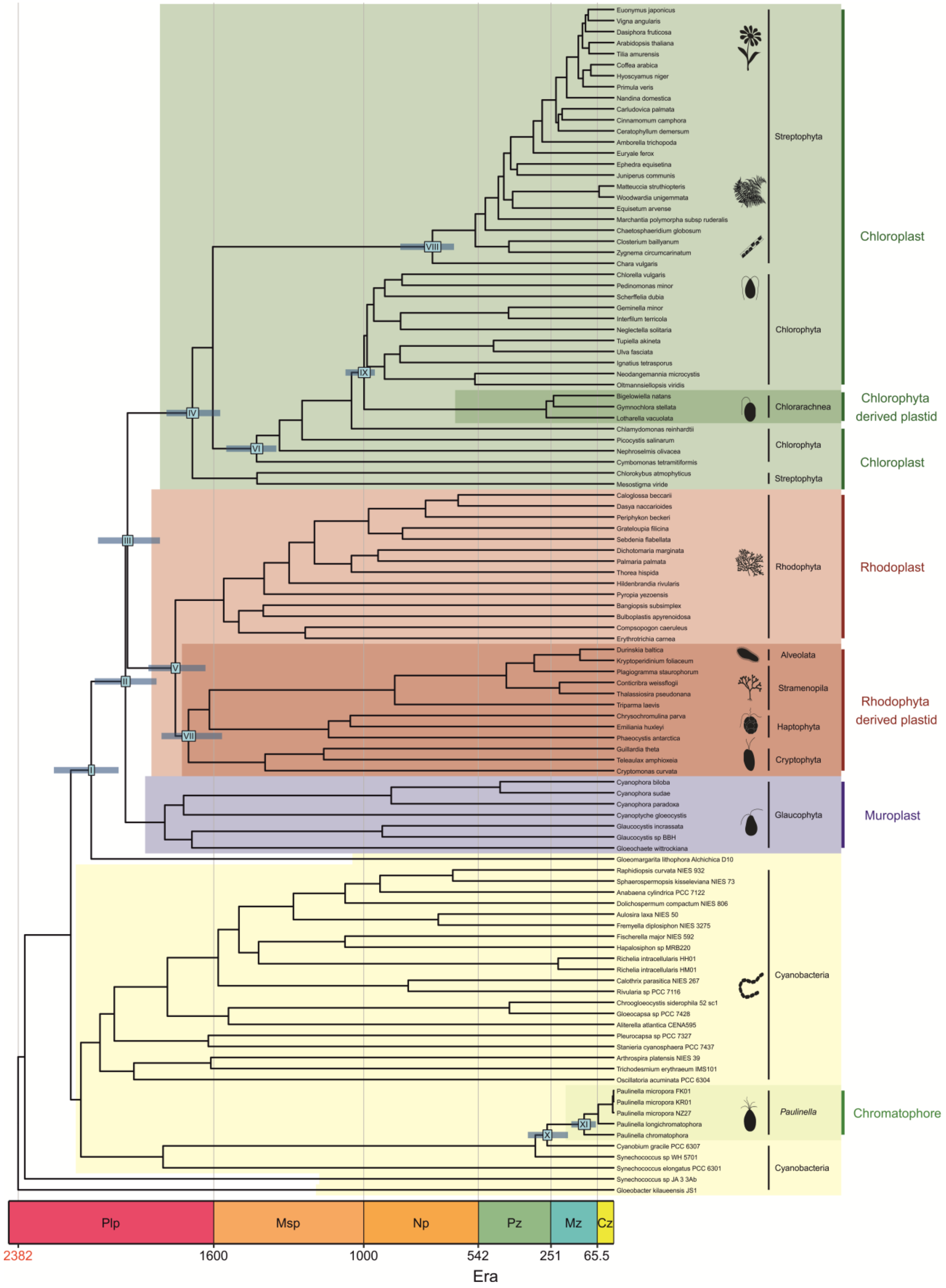

Euonymus japonicus
Vigna angularis
Dasiphora fruticosa
Arabidopsis thaliana
Tilia amurensis
Coffea arabica
Hyoscyamus niger
Primula veris
Nandina domestica
Carludovica palmata
Cinnamomum camphora
Ceratophyllum demersum
Amborella trichopoda
Euryale ferox
Ephedra equisetina
Juniperus communis
Matteuccia struthiopteris
Woodwardia unigemmata
Equisetum arvense
Marchantia polymorpha subsp ruderalis
Chaetosphaeridium globosum
Closterium baillyanum
Zygnema circumcarinatum
Chara vulgaris
Chlorella vulgaris
Pedinomonas minor
Scherffelia dubia
Geminella minor
Interfilum terricola
Neglectella solitaria
Tupiella akineta
Ulva fasciata
Ignatius tetrasporus
Neodangemannia microcystis
Oltmannsiellopsis viridis
Bigelowiella natans
Gymnochlora stellata
Lotharella vacuolata
Chlamydomonas reinhardtii
Picocystis salinarum
Nephroselmis olivacea
Cymbomonas tetramitiformis
Chlorokybus atmophyticus
Mesostigma viride
Caloglossa beccarii
Dasya naccarioides
Periphykon beckeri
Grateloupia filicina
Sebdenia flabellata
Dichotomaria marginata
Palmaria palmata
Thorea hispida
Hildenbrandia rivularis
Pyropia yezoensis
Bangiopsis subsimplex
Bulboplastis apyrenoidosa
Compsopogon caeruleus
Erythrotrichia carnea
Durinskia baltica
Kryptoperidinium foliaceum
Plagiogramma staurophorum
Conticribra weissflogii
Thalassiosira pseudonana
Triparma laevis
Chrysochromulina parva
Emiliania huxleyi
Phaeocystis antarctica
Guillardia theta
Teleaulax amphioxeia
Cryptomonas curvata
Cyanophora biloba
Cyanophora sudae
Cyanophora paradoxa
Cyanoptyche gloeocystis
Glaucocystis incrassata
Glaucocystis sp BBH
Gloeochaete wittrockiana
Gloeomargarita lithophora Alchichica D10
Raphidiopsis curvata NIES 932
Sphaerospermopsis kisseleviana NIES 73
Anabaena cylindrica PCC 7122
Dolichospermum compactum NIES 806
Aulosira laxa NIES 50
Fremyella diplosiphon NIES 3275
Fischerella major NIES 592
Hapalosiphon sp MRB220
Richelia intracellularis HH01
Richelia intracellularis HM01
Calothrix parasitica NIES 267
Rivularia sp PCC 7116
Chroogloeocystis siderophila 52 sc1
Gloeocapsa sp PCC 7428
Aliterella atlantica CENA595
Pleurocapsa sp PCC 7327
Stanieria cyanosphaera PCC 7437
Arthrospira platensis NIES 39
Trichodesmium erythraeum IMS101
Oscillatoria acuminata PCC 6304
Paulinella micropora FK01
Paulinella micropora KR01
Paulinella micropora NZ27
Paulinella longichromatophora
Paulinella chromatophora
Cyanobium gracile PCC 6307
Synechococcus sp WH 5701
Synechococcus elongatus PCC 6301
Synechococcus sp JA 3 3Ab
Gloeobacter kilaueensis JS1
Streptophyta
Chlorophyta
Chlorarachnea
Chlorophyta
Streptophyta
Rhodophyta
Alveolata
Stramenopila
Haptophyta
Cryptophyta
Glaucophyta
Cyanobacteria
Paulinella
Cyanobacteria
Chloroplast
Chlorophyta derived plastid
Chloroplast
Rhodoplast
Rhodophyta derived plastid
Muroplast
Chromatophore
I
II
III
IV
V
VI
VII
VIII
IX
X
XI
Plp
Msp
Np
Pz
Mz
Cz
2382
1600
1000
542
251
65.5
Era

(See figure on the previous page) **Figure S4.** Time-calibrated phylogeny of photosynthetic organelles and cyanobacteria. The divergence times were inferred with PhyloBayes under LN model and calibrated with C3 (Tab. 1, S7). The tree topology was reconstructed in IQ-TREE. Other description as in Figure S1.

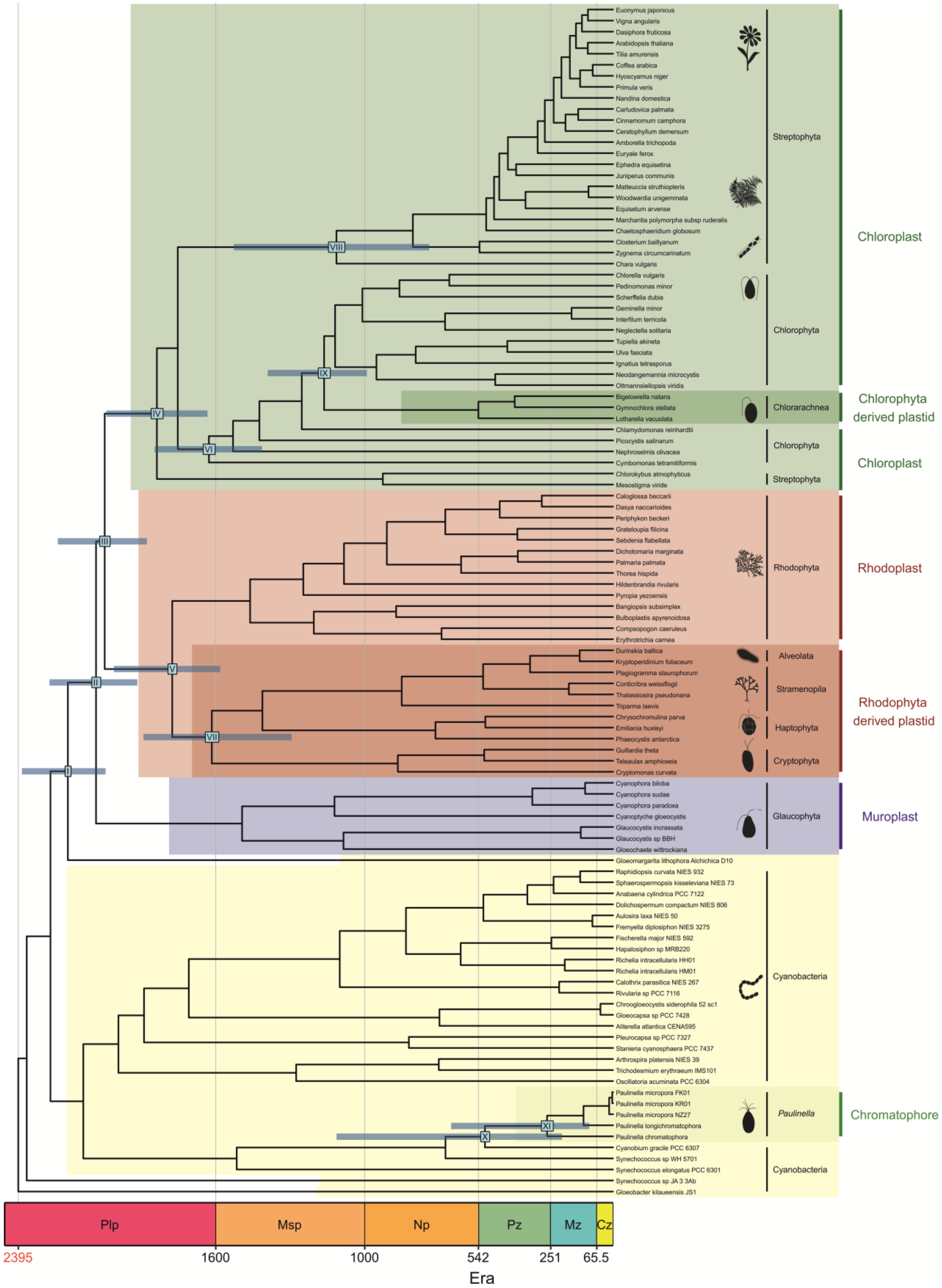
Euonymus japonicus
Vigna angularis
Dasiphora fruticosa
Arabidopsis thaliana
Tilia amurensis
Coffea arabica
Hyoscyamus niger
Primula veris
Nandina domestica
Carludovica palmata
Cinnamomum camphora
Ceratophyllum demersum
Amborella trichopoda
Euryale ferox
Ephedra equisetina
Juniperus communis
Matteuccia struthiopteris
Woodwardia unigemmata
Equisetum arvense
Marchantia polymorpha subsp ruderalis
Chaetosphaeridium globosum
Closterium baillyanum
Zygnema circumcarinatum
Chara vulgaris
Chlorella vulgaris
Pedinomonas minor
Scherffelia dubia
Geminella minor
Interfilum terricola
Neglectella solitaria
Tupiella akineta
Ulva fasciata
Ignatius tetrasporus
Neodangemannia microcystis
Oltmannsiellopsis viridis
Bigelowiella natans
Gymnochlora stellata
Lotharella vacuolata
Chlamydomonas reinhardtii
Picocystis salinarum
Nephroselmis olivacea
Cymbomonas tetramitiformis
Chlorokybus atmophyticus
Mesostigma viride
Caloglossa beccarii
Dasya naccarioides
Periphykon beckeri
Grateloupia filicina
Sebdenia flabellata
Dichotomaria marginata
Palmaria palmata
Thorea hispida
Hildenbrandia rivularis
Pyropia yezoensis
Bangiopsis subsimplex
Bulboplastis apyrenoidosa
Compsopogon caeruleus
Erythrotrichia carnea
Durinskia baltica
Kryptoperidinium foliaceum
Plagiogramma staurophorum
Conticribra weissflogii
Thalassiosira pseudonana
Triparma laevis
Chrysochromulina parva
Emiliania huxleyi
Phaeocystis antarctica
Guillardia theta
Teleaulax amphioxeia
Cryptomonas curvata
Cyanophora biloba
Cyanophora sudae
Cyanophora paradoxa
Cyanoptyche gloeocystis
Glaucocystis incrassata
Glaucocystis sp BBH
Gloeochaete wittrockiana
Gloeomargarita lithophora Alchichica D10
Raphidiopsis curvata NIES 932
Sphaerospermopsis kisseleviana NIES 73
Anabaena cylindrica PCC 7122
Dolichospermum compactum NIES 806
Aulosira laxa NIES 50
Fremyella diplosiphon NIES 3275
Fischerella major NIES 592
Hapalosiphon sp MRB220
Richelia intracellularis HH01
Richelia intracellularis HM01
Calothrix parasitica NIES 267
Rivularia sp PCC 7116
Chroogloeocystis siderophila 52 sc1
Gloeocapsa sp PCC 7428
Aliterella atlantica CENA595
Pleurocapsa sp PCC 7327
Stanieria cyanosphaera PCC 7437
Arthrospira platensis NIES 39
Trichodesmium erythraeum IMS101
Oscillatoria acuminata PCC 6304
Paulinella micropora FK01
Paulinella micropora KR01
Paulinella micropora NZ27
Paulinella longichromatophora
Paulinella chromatophora
Cyanobium gracile PCC 6307
Synechococcus sp WH 5701
Synechococcus elongatus PCC 6301
Synechococcus sp JA 3 3Ab
Gloeobacter kilaueensis JS1
I
II
III
IV
V
VI
VII
VIII
IX
X
XI
Streptophyta
Chlorophyta
Chlorarachnea
Chlorophyta
Streptophyta
Rhodophyta
Alveolata
Stramenopila
Haptophyta
Cryptophyta
Glaucophyta
Cyanobacteria
Paulinella
Cyanobacteria
Chloroplast
Chlorophyta derived plastid
Chloroplast
Rhodoplast
Rhodophyta derived plastid
Muroplast
Chromatophore
Plp
Msp
Np
Pz
Mz
Cz
2395
1600
1000
542
251
65.5
Era

(See figure on the previous page) **Figure S5.** Time-calibrated phylogeny of photosynthetic organelles and cyanobacteria. The divergence times were inferred with PhyloBayes under UGAM model and calibrated with C3 (Tab. 1, S7). The tree topology was reconstructed in IQ-TREE. Other description as in Figure S1.

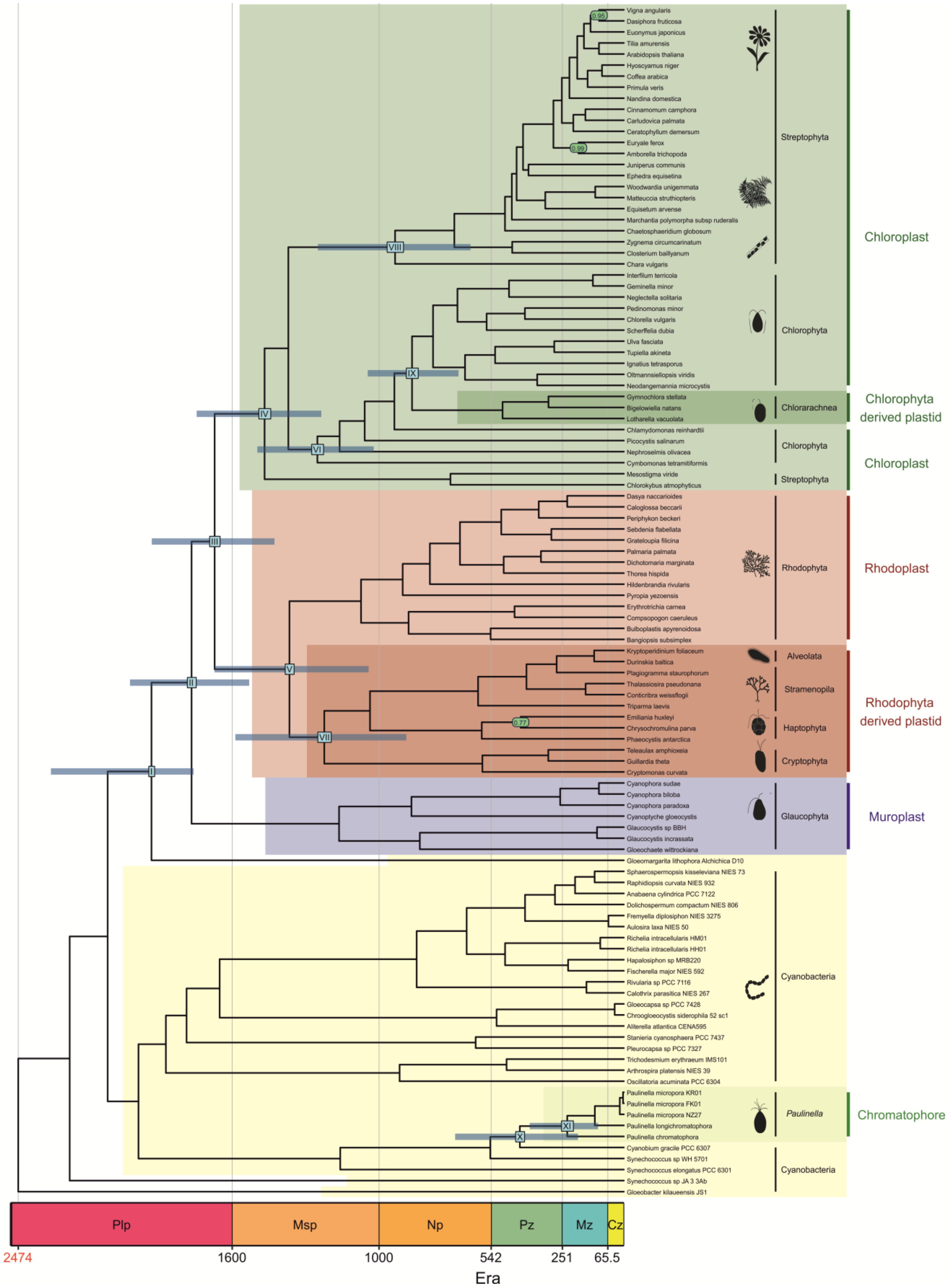
Vigna angularis
Dasiphora fruticosa
Euonymus japonicus
Tilia amurensis
Arabidopsis thaliana
Hyoscyamus niger
Coffea arabica
Primula veris
Nandina domestica
Cinnamomum camphora
Carludovica palmata
Ceratophyllum demersum
Euryale ferox
Amborella trichopoda
Juniperus communis
Ephedra equisetina
Woodwardia unigemmata
Matteuccia struthiopteris
Equisetum arvense
Marchantia polymorpha subsp ruderalis
Chaetosphaeridium globosum
Zygnema circumcarinatum
Closterium baillyanum
Chara vulgaris
Interfilum terricola
Geminella minor
Neglectella solitaria
Pedinomonas minor
Chlorella vulgaris
Scherffelia dubia
Ulva fasciata
Tupiella akineta
Ignatius tetrasporus
Oltmannsiellopsis viridis
Neodangemannia microcystis
Gymnochlora stellata
Bigelowiella natans
Lotharella vacuolata
Chlamydomonas reinhardtii
Picocystis salinarum
Nephroselmis olivacea
Cymbomonas tetramitiformis
Mesostigma viride
Chlorokybus atmophyticus
Dasya naccarioides
Caloglossa beccarii
Periphykon beckeri
Sebdenia flabellata
Grateloupia filicina
Palmaria palmata
Dichotomaria marginata
Thorea hispida
Hildenbrandia rivularis
Pyropia yezoensis
Erythrotrichia carnea
Compsopogon caeruleus
Bulboplastis apyrenoidosa
Bangiopsis subsimplex
Kryptoperidinium foliaceum
Durinskia baltica
Plagiogramma staurophorum
Thalassiosira pseudonana
Conticribra weissflogii
Triparma laevis
Emiliania huxleyi
Chrysochromulina parva
Phaeocystis antarctica
Teleaulax amphioxeia
Guillardia theta
Cryptomonas curvata
Cyanophora sudae
Cyanophora biloba
Cyanophora paradoxa
Cyanoptyche gloeocystis
Glaucocystis sp BBH
Glaucocystis incrassata
Gloeochaete wittrockiana
Gloeomargarita lithophora Alchichica D10
Sphaerospermopsis kisseleviana NIES 73
Raphidiopsis curvata NIES 932
Anabaena cylindrica PCC 7122
Dolichospermum compactum NIES 806
Fremyella diplosiphon NIES 3275
Aulosira laxa NIES 50
Richelia intracellularis HM01
Richelia intracellularis HH01
Hapalosiphon sp MRB220
Fischerella major NIES 592
Rivularia sp PCC 7116
Calothrix parasitica NIES 267
Gloeocapsa sp PCC 7428
Chroogloeocystis siderophila 52 sc1
Aliterella atlantica CENA595
Stanieria cyanosphaera PCC 7437
Pleurocapsa sp PCC 7327
Trichodesmium erythraeum IMS101
Arthrospira platensis NIES 39
Oscillatoria acuminata PCC 6304
Paulinella micropora KR01
Paulinella micropora FK01
Paulinella micropora NZ27
Paulinella longichromatophora
Paulinella chromatophora
Cyanobium gracile PCC 6307
Synechococcus sp WH 5701
Synechococcus elongatus PCC 6301
Synechococcus sp JA 3 3Ab
Gloeobacter kilaueensis JS1
0.95
0.99
0.77
I
II
III
IV
V
VI
VII
VIII
IX
X
XI
Streptophyta
Chlorophyta
Chlorarachnea
Chlorophyta
Streptophyta
Rhodophyta
Alveolata
Stramenopila
Haptophyta
Cryptophyta
Glaucophyta
Cyanobacteria
Paulinella
Cyanobacteria
Chloroplast
Chlorophyta derived plastid
Chloroplast
Rhodoplast
Rhodophyta derived plastid
Muroplast
Chromatophore
Plp
Msp
Np
Pz
Mz
Cz
2474
1600
1000
542
251
65.5
Era

(See figure on the previous page) **Figure S6.** Time-calibrated phylogeny of photosynthetic organelles and cyanobacteria. The tree was inferred with Beast under UCLNR model and calibrated with C1 (Tab. 1, S7). Other description as in Figure S1.

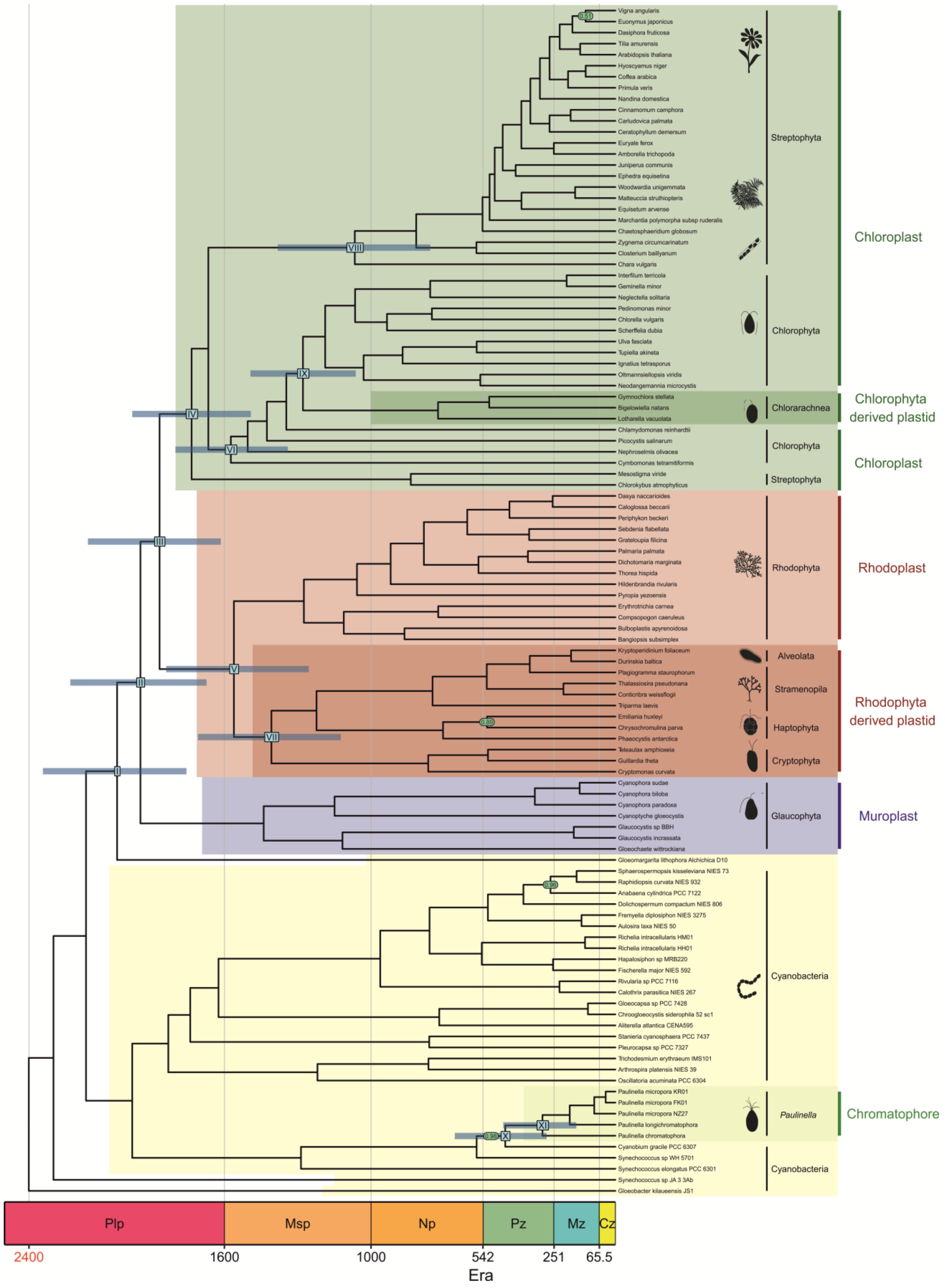

Vigna angularis
Euonymus japonicus
Dasiphora fruticosa
Tilia amurensis
Arabidopsis thaliana
Hyoscyamus niger
Coffea arabica
Primula veris
Nandina domestica
Cinnamomum camphora
Carludovica palmata
Ceratophyllum demersum
Euryale ferox
Amborella trichopoda
Juniperus communis
Ephedra equisetina
Woodwardia unigemmata
Matteuccia struthiopteris
Equisetum arvense
Marchantia polymorpha subsp ruderalis
Chaetosphaeridium globosum
Zygnema circumcarinatum
Closterium baillyanum
Chara vulgaris
Interfilum terricola
Geminella minor
Neglectella solitaria
Pedinomonas minor
Chlorella vulgaris
Scherffelia dubia
Ulva fasciata
Tupiella akineta
Ignatius tetrasporus
Oltmannsiellopsis viridis
Neodangemannia microcystis
Gymnochlora stellata
Bigelowiella natans
Lotharella vacuolata
Chlamydomonas reinhardtii
Picocystis salinarum
Nephroselmis olivacea
Cymbomonas tetramitiformis
Mesostigma viride
Chlorokybus atmophyticus
Dasya naccarioides
Caloglossa beccarii
Periphykon beckeri
Sebdenia flabellata
Grateloupia filicina
Palmaria palmata
Dichotomaria marginata
Thorea hispida
Hildenbrandia rivularis
Pyropia yezoensis
Erythrotrichia carnea
Compsopogon caeruleus
Bulboplastis apyrenoidosa
Bangiopsis subsimplex
Kryptoperidinium foliaceum
Durinskia baltica
Plagiogramma staurophorum
Thalassiosira pseudonana
Conticribra weissflogii
Triparma laevis
Emiliania huxleyi
Chrysochromulina parva
Phaeocystis antarctica
Teleaulax amphioxeia
Guillardia theta
Cryptomonas curvata
Cyanophora sudae
Cyanophora biloba
Cyanophora paradoxa
Cyanoptyche gloeocystis
Glaucocystis sp BBH
Glaucocystis incrassata
Gloeochaete wittrockiana
Gloeomargarita lithophora Alchichica D10
Sphaerospermopsis kisseleviana NIES 73
Raphidiopsis curvata NIES 932
Anabaena cylindrica PCC 7122
Dolichospermum compactum NIES 806
Fremyella diplosiphon NIES 3275
Aulosira laxa NIES 50
Richelia intracellularis HM01
Richelia intracellularis HH01
Hapalosiphon sp MRB220
Fischerella major NIES 592
Rivularia sp PCC 7116
Calothrix parasitica NIES 267
Gloeocapsa sp PCC 7428
Chroogloeocystis siderophila 52 sc1
Aliterella atlantica CENA595
Stanieria cyanosphaera PCC 7437
Pleurocapsa sp PCC 7327
Trichodesmium erythraeum IMS101
Arthrospira platensis NIES 39
Oscillatoria acuminata PCC 6304
Paulinella micropora KR01
Paulinella micropora FK01
Paulinella micropora NZ27
Paulinella longichromatophora
Paulinella chromatophora
Cyanobium gracile PCC 6307
Synechococcus sp WH 5701
Synechococcus elongatus PCC 6301
Synechococcus sp JA 3 3Ab
Gloeobacter kilaueensis JS1
Streptophyta
Chlorophyta
Chlorarachnea
Chlorophyta
Streptophyta
Rhodophyta
Alveolata
Stramenopila
Haptophyta
Cryptophyta
Glaucophyta
Cyanobacteria
Paulinella
Cyanobacteria
Chloroplast
Chlorophyta derived plastid
Chloroplast
Rhodoplast
Rhodophyta derived plastid
Muroplast
Chromatophore
0.51
0.89
0.96
0.98
VIII
IX
IV
VI
III
V
II
VII
I
XI
X
Plp
Msp
Np
Pz
Mz
Cz
2400
1600
1000
542
251
65.5
Era

(See figure on the previous page) **Figure S7.** Time-calibrated phylogeny of photosynthetic organelles and cyanobacteria. The tree was inferred with MrBayes under IGR model and calibrated with C1 (Tab. 1, S7). Other description as in Figure S1.

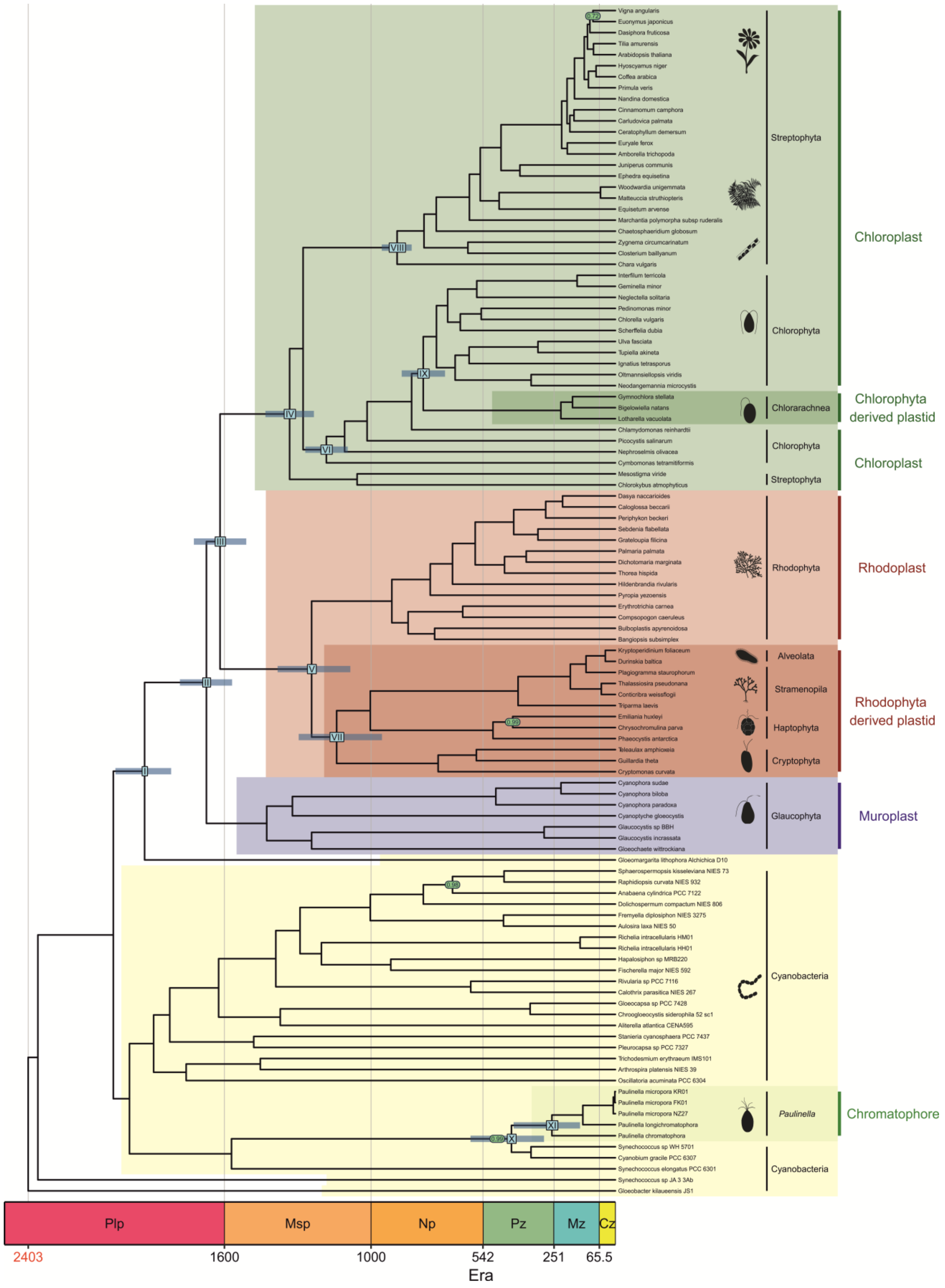

Vigna angularis
0.72
Euonymus japonicus
Dasiphora fruticosa
Tilia amurensis
Arabidopsis thaliana
Hyoscyamus niger
Coffea arabica
Primula veris
Nandina domestica
Cinnamomum camphora
Carludovica palmata
Ceratophyllum demersum
Euryale ferox
Amborella trichopoda
Juniperus communis
Ephedra equisetina
Woodwardia unigemmata
Matteuccia struthiopteris
Equisetum arvense
Marchantia polymorpha subsp ruderalis
Chaetosphaeridium globosum
Zygnema circumcarinatum
Closterium baillyanum
Chara vulgaris
Interfilum terricola
Geminella minor
Neglectella solitaria
Pedinomonas minor
Chlorella vulgaris
Scherffelia dubia
Ulva fasciata
Tupiella akineta
Ignatius tetrasporus
Oltmannsiellopsis viridis
Neodangemannia microcystis
Gymnochlora stellata
Bigelowiella natans
Lotharella vacuolata
Chlamydomonas reinhardtii
Picocystis salinarum
Nephroselmis olivacea
Cymbomonas tetramitiformis
Mesostigma viride
Chlorokybus atmophyticus
Dasya naccarioides
Caloglossa beccarii
Periphykon beckeri
Sebdenia flabellata
Grateloupia filicina
Palmaria palmata
Dichotomaria marginata
Thorea hispida
Hildenbrandia rivularis
Pyropia yezoensis
Erythrotrichia carnea
Compsopogon caeruleus
Bulboplastis apyrenoidosa
Bangiopsis subsimplex
Kryptoperidinium foliaceum
Durinskia baltica
Plagiogramma staurophorum
Thalassiosira pseudonana
Conticribra weissflogii
Triparma laevis
Emiliania huxleyi
0.99
Chrysochromulina parva
Phaeocystis antarctica
Teleaulax amphioxeia
Guillardia theta
Cryptomonas curvata
Cyanophora sudae
Cyanophora biloba
Cyanophora paradoxa
Cyanoptyche gloeocystis
Glaucocystis sp BBH
Glaucocystis incrassata
Gloeochaete wittrockiana
Gloeomargarita lithophora Alchichica D10
Sphaerospermopsis kisseleviana NIES 73
Raphidiopsis curvata NIES 932
0.98
Anabaena cylindrica PCC 7122
Dolichospermum compactum NIES 806
Fremyella diplosiphon NIES 3275
Aulosira laxa NIES 50
Richelia intracellularis HM01
Richelia intracellularis HH01
Hapalosiphon sp MRB220
Fischerella major NIES 592
Rivularia sp PCC 7116
Calothrix parasitica NIES 267
Gloeocapsa sp PCC 7428
Chroogloeocystis siderophila 52 sc1
Aliterella atlantica CENA595
Stanieria cyanosphaera PCC 7437
Pleurocapsa sp PCC 7327
Trichodesmium erythraeum IMS101
Arthrospira platensis NIES 39
Oscillatoria acuminata PCC 6304
Paulinella micropora KR01
Paulinella micropora FK01
Paulinella micropora NZ27
Paulinella longichromatophora
Paulinella chromatophora
0.99
Synechococcus sp WH 5701
Cyanobium gracile PCC 6307
Synechococcus elongatus PCC 6301
Synechococcus sp JA 3 3Ab
Gloeobacter kilaueensis JS1
I
II
III
IV
V
VI
VII
VIII
IX
X
XI
Streptophyta
Chlorophyta
Chlorarachnea
Chlorophyta
Streptophyta
Rhodophyta
Alveolata
Stramenopila
Haptophyta
Cryptophyta
Glaucophyta
Cyanobacteria
Paulinella
Cyanobacteria
Chloroplast
Chlorophyta derived plastid
Chloroplast
Rhodoplast
Rhodophyta derived plastid
Muroplast
Chromatophore
Plp
Msp
Np
Pz
Mz
Cz
2403
1600
1000
542
251
65.5
Era

(See figure on the previous page) **Figure S8.** Time-calibrated phylogeny of photosynthetic organelles and cyanobacteria. The tree was inferred with MrBayes under TK02 model and calibrated with C1 (Tab. 1, S7). Other description as in Figure S1.

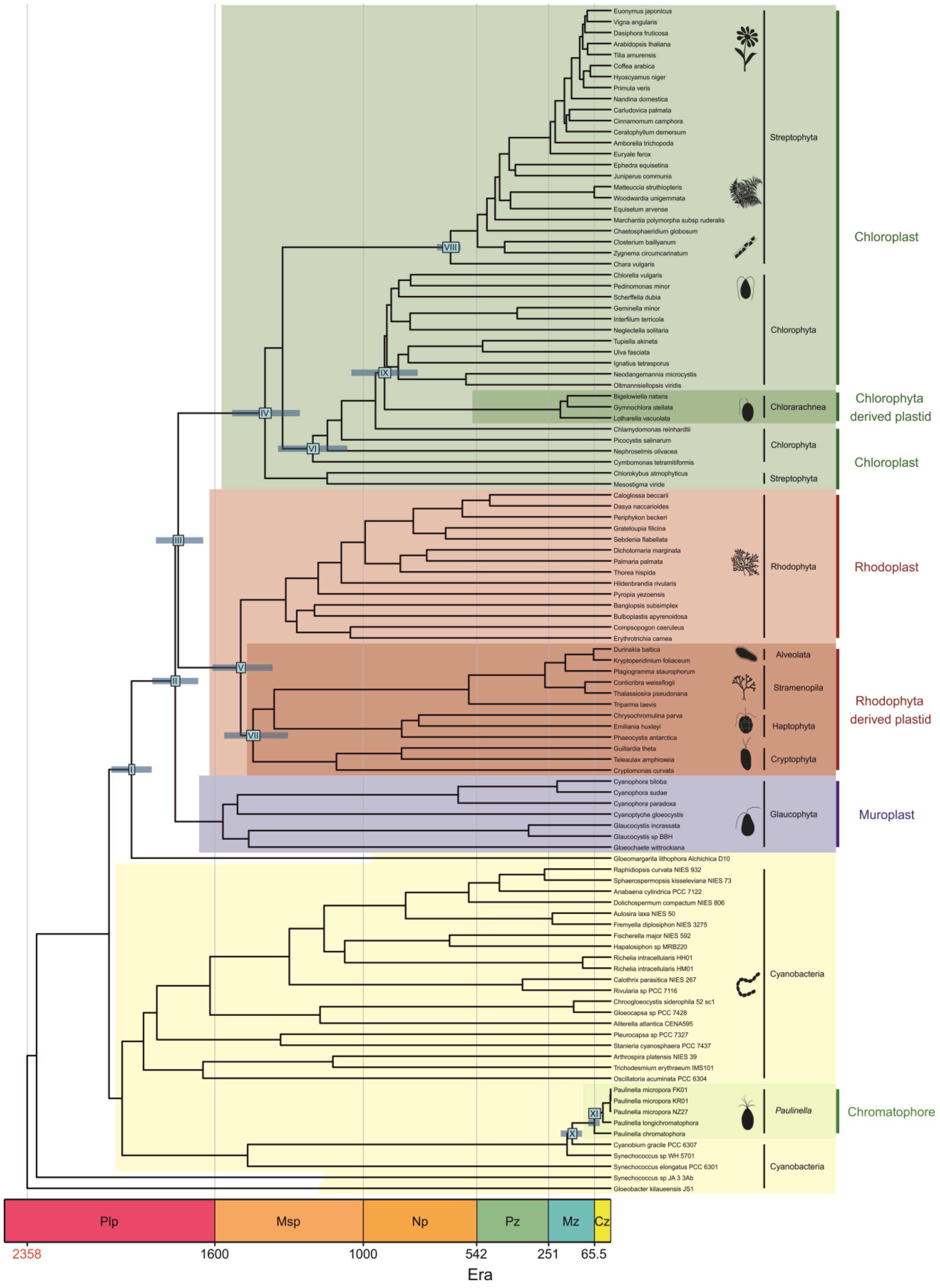

Euonymus japonicus
Vigna angularis
Dasiphora fruticosa
Arabidopsis thaliana
Tilia amurensis
Coffea arabica
Hyoscyamus niger
Primula veris
Nandina domestica
Carludovica palmata
Cinnamomum camphora
Ceratophyllum demersum
Amborella trichopoda
Euryale ferox
Ephedra equisetina
Juniperus communis
Matteuccia struthiopteris
Woodwardia unigemmata
Equisetum arvense
Marchantia polymorpha subsp ruderalis
Chaetosphaeridium globosum
Closterium baillyanum
Zygnema circumcarinatum
Chara vulgaris
Chlorella vulgaris
Pedinomonas minor
Scherffelia dubia
Geminella minor
Interfilum terricola
Neglectella solitaria
Tupiella akineta
Ulva fasciata
Ignatius tetrasporus
Neodangemannia microcystis
Oltmannsiellopsis viridis
Bigelowiella natans
Gymnochlora stellata
Lotharella vacuolata
Chlamydomonas reinhardtii
Picocystis salinarum
Nephroselmis olivacea
Cymbomonas tetramitiformis
Chlorokybus atmophyticus
Mesostigma viride
Caloglossa beccarii
Dasya naccarioides
Periphykon beckeri
Grateloupia filicina
Sebdenia flabellata
Dichotomaria marginata
Palmaria palmata
Thorea hispida
Hildenbrandia rivularis
Pyropia yezoensis
Bangiopsis subsimplex
Bulboplastis apyrenoidosa
Compsopogon caeruleus
Erythrotrichia carnea
Durinskia baltica
Kryptoperidinium foliaceum
Plagiogramma staurophorum
Conticribra weissflogii
Thalassiosira pseudonana
Triparma laevis
Chrysochromulina parva
Emiliania huxleyi
Phaeocystis antarctica
Guillardia theta
Teleaulax amphioxeia
Cryptomonas curvata
Cyanophora biloba
Cyanophora sudae
Cyanophora paradoxa
Cyanoptyche gloeocystis
Glaucocystis incrassata
Glaucocystis sp BBH
Gloeochaete wittrockiana
Gloeomargarita lithophora Alchichica D10
Raphidiopsis curvata NIES 932
Sphaerospermopsis kisseleviana NIES 73
Anabaena cylindrica PCC 7122
Dolichospermum compactum NIES 806
Aulosira laxa NIES 50
Fremyella diplosiphon NIES 3275
Fischerella major NIES 592
Hapalosiphon sp MRB220
Richelia intracellularis HH01
Richelia intracellularis HM01
Calothrix parasitica NIES 267
Rivularia sp PCC 7116
Chroogloeocystis siderophila 52 sc1
Gloeocapsa sp PCC 7428
Aliterella atlantica CENA595
Pleurocapsa sp PCC 7327
Stanieria cyanosphaera PCC 7437
Arthrospira platensis NIES 39
Trichodesmium erythraeum IMS101
Oscillatoria acuminata PCC 6304
Paulinella micropora FK01
Paulinella micropora KR01
Paulinella micropora NZ27
Paulinella longichromatophora
Paulinella chromatophora
Cyanobium gracile PCC 6307
Synechococcus sp WH 5701
Synechococcus elongatus PCC 6301
Synechococcus sp JA 3 3Ab
Gloeobacter kilaueensis JS1
Streptophyta
Chlorophyta
Chlorarachnea
Chlorophyta
Streptophyta
Rhodophyta
Alveolata
Stramenopila
Haptophyta
Cryptophyta
Glaucophyta
Cyanobacteria
Paulinella
Cyanobacteria
Chloroplast
Chlorophyta derived plastid
Chloroplast
Rhodoplast
Rhodophyta derived plastid
Muroplast
Chromatophore
I
II
III
IV
V
VI
VII
VIII
IX
X
XI
Plp
Msp
Np
Pz
Mz
Cz
2358
1600
1000
542
251
65.5
Era

(See figure on the previous page) **Figure S9.** Time-calibrated phylogeny of photosynthetic organelles and cyanobacteria. The divergence times were inferred with PhyloBayes under CIR model and calibrated with C1 (Tab. 1, S7). The tree topology was reconstructed in IQ-TREE. Other description as in Figure S1.

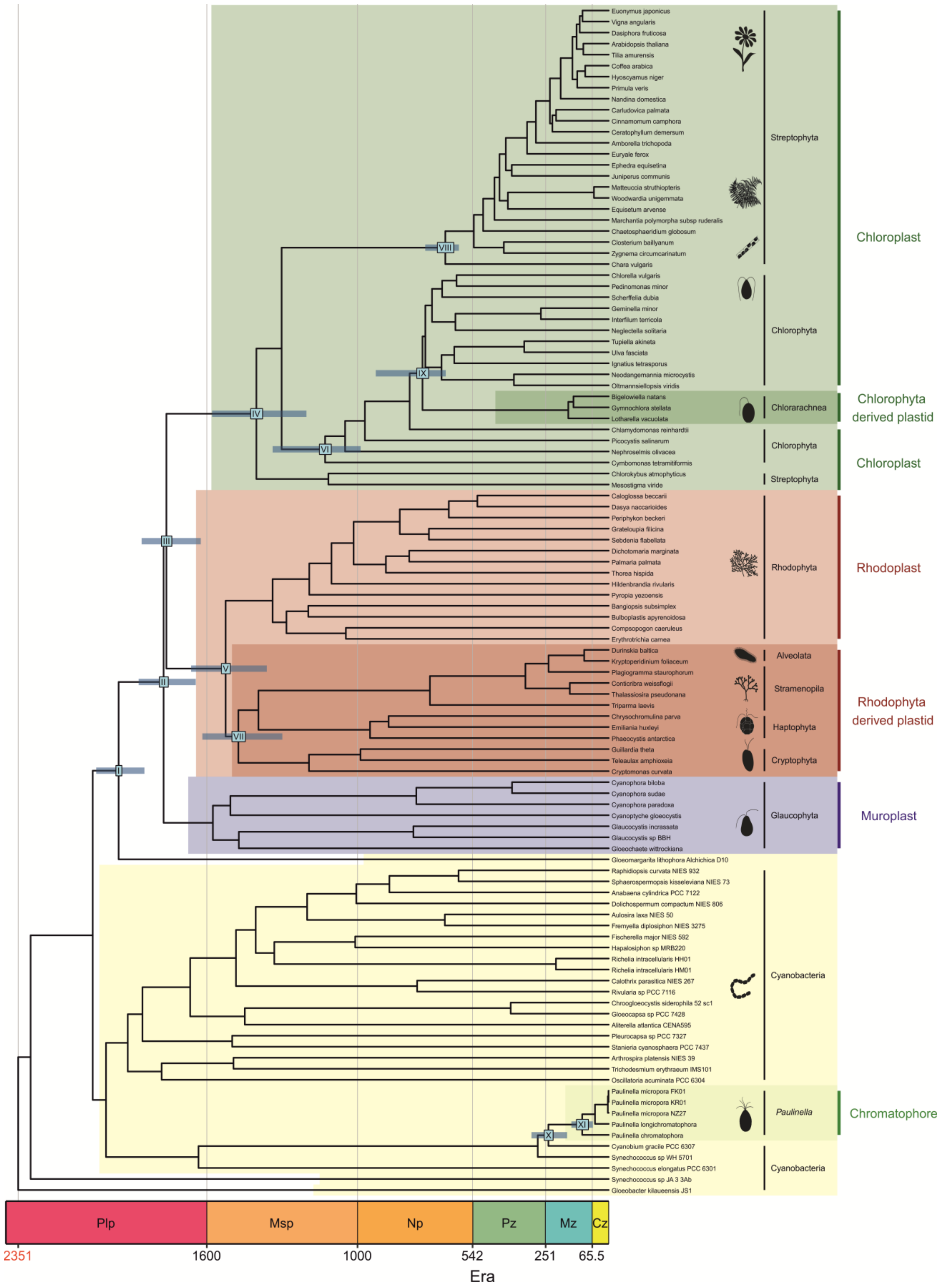

Euonymus japonicus
Vigna angularis
Dasiphora fruticosa
Arabidopsis thaliana
Tilia amurensis
Coffea arabica
Hyoscyamus niger
Primula veris
Nandina domestica
Carludovica palmata
Cinnamomum camphora
Ceratophyllum demersum
Amborella trichopoda
Euryale ferox
Ephedra equisetina
Juniperus communis
Matteuccia struthiopteris
Woodwardia unigemmata
Equisetum arvense
Marchantia polymorpha subsp ruderalis
Chaetosphaeridium globosum
Closterium baillyanum
Zygnema circumcarinatum
Chara vulgaris
Chlorella vulgaris
Pedinomonas minor
Scherffelia dubia
Geminella minor
Interfilum terricola
Neglectella solitaria
Tupiella akineta
Ulva fasciata
Ignatius tetrasporus
Neodangemannia microcystis
Oltmannsiellopsis viridis
Bigelowiella natans
Gymnochlora stellata
Lotharella vacuolata
Chlamydomonas reinhardtii
Picocystis salinarum
Nephroselmis olivacea
Cymbomonas tetramitiformis
Chlorokybus atmophyticus
Mesostigma viride
Caloglossa beccarii
Dasya naccarioides
Periphykon beckeri
Grateloupia filicina
Sebdenia flabellata
Dichotomaria marginata
Palmaria palmata
Thorea hispida
Hildenbrandia rivularis
Pyropia yezoensis
Bangiopsis subsimplex
Bulboplastis apyrenoidosa
Compsopogon caeruleus
Erythrotrichia carnea
Durinskia baltica
Kryptoperidinium foliaceum
Plagiogramma staurophorum
Conticribra weissflogii
Thalassiosira pseudonana
Triparma laevis
Chrysochromulina parva
Emiliania huxleyi
Phaeocystis antarctica
Guillardia theta
Teleaulax amphioxeia
Cryptomonas curvata
Cyanophora biloba
Cyanophora sudae
Cyanophora paradoxa
Cyanoptyche gloeocystis
Glaucocystis incrassata
Glaucocystis sp BBH
Gloeochaete wittrockiana
Gloeomargarita lithophora Alchichica D10
Raphidiopsis curvata NIES 932
Sphaerospermopsis kisseleviana NIES 73
Anabaena cylindrica PCC 7122
Dolichospermum compactum NIES 806
Aulosira laxa NIES 50
Fremyella diplosiphon NIES 3275
Fischerella major NIES 592
Hapalosiphon sp MRB220
Richelia intracellularis HH01
Richelia intracellularis HM01
Calothrix parasitica NIES 267
Rivularia sp PCC 7116
Chroogloeocystis siderophila 52 sc1
Gloeocapsa sp PCC 7428
Aliterella atlantica CENA595
Pleurocapsa sp PCC 7327
Stanieria cyanosphaera PCC 7437
Arthrospira platensis NIES 39
Trichodesmium erythraeum IMS101
Oscillatoria acuminata PCC 6304
Paulinella micropora FK01
Paulinella micropora KR01
Paulinella micropora NZ27
Paulinella longichromatophora
Paulinella chromatophora
Cyanobium gracile PCC 6307
Synechococcus sp WH 5701
Synechococcus elongatus PCC 6301
Synechococcus sp JA 3 3Ab
Gloeobacter kilaueensis JS1
Streptophyta
Chlorophyta
Chlorarachnea
Chlorophyta
Streptophyta
Rhodophyta
Alveolata
Stramenopila
Haptophyta
Cryptophyta
Glaucophyta
Cyanobacteria
Paulinella
Cyanobacteria
Chloroplast
Chlorophyta derived plastid
Chloroplast
Rhodoplast
Rhodophyta derived plastid
Muroplast
Chromatophore
I
II
III
IV
V
VI
VII
VIII
IX
X
XI
Plp
Msp
Np
Pz
Mz
Cz
2351
1600
1000
542
251
65.5
Era

(See figure on the previous page) **Figure S10.** Time-calibrated phylogeny of photosynthetic organelles and cyanobacteria. The divergence times were inferred with PhyloBayes under LN model and calibrated with C1 (Tab. 1, S7). The tree topology was reconstructed in IQ-TREE. Other description as in Figure S1.

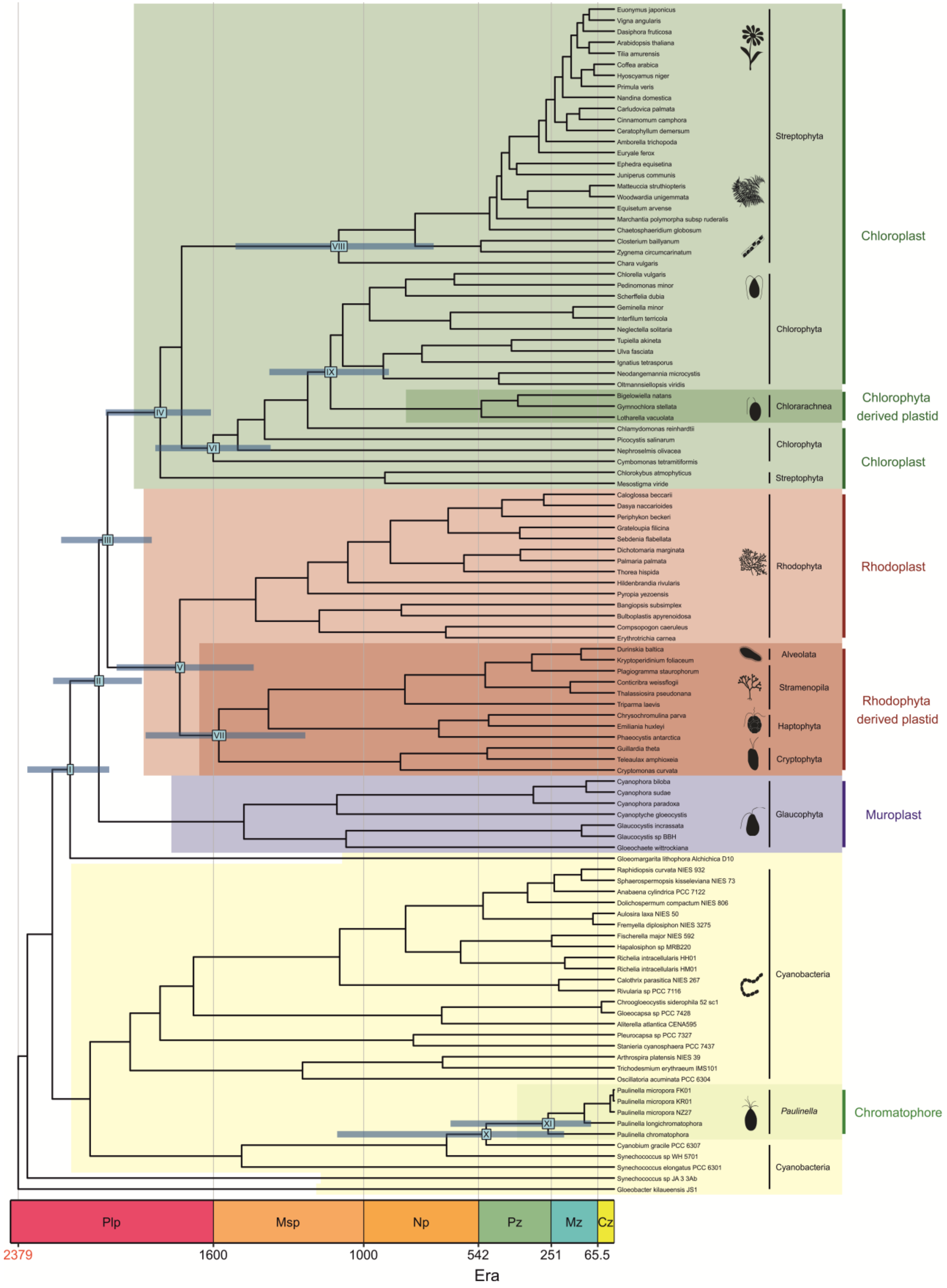

Euonymus japonicus
Vigna angularis
Dasiphora fruticosa
Arabidopsis thaliana
Tilia amurensis
Coffea arabica
Hyoscyamus niger
Primula veris
Nandina domestica
Carludovica palmata
Cinnamomum camphora
Ceratophyllum demersum
Amborella trichopoda
Euryale ferox
Ephedra equisetina
Juniperus communis
Matteuccia struthiopteris
Woodwardia unigemmata
Equisetum arvense
Marchantia polymorpha subsp ruderalis
Chaetosphaeridium globosum
Closterium baillyanum
Zygnema circumcarinatum
Chara vulgaris
Chlorella vulgaris
Pedinomonas minor
Scherffelia dubia
Geminella minor
Interfilum terricola
Neglectella solitaria
Tupiella akineta
Ulva fasciata
Ignatius tetrasporus
Neodangemannia microcystis
Oltmannsiellopsis viridis
Bigelowiella natans
Gymnochlora stellata
Lotharella vacuolata
Chlamydomonas reinhardtii
Picocystis salinarum
Nephroselmis olivacea
Cymbomonas tetramitiformis
Chlorokybus atmophyticus
Mesostigma viride
Caloglossa beccarii
Dasya naccarioides
Periphykon beckeri
Grateloupia filicina
Sebdenia flabellata
Dichotomaria marginata
Palmaria palmata
Thorea hispida
Hildenbrandia rivularis
Pyropia yezoensis
Bangiopsis subsimplex
Bulboplastis apyrenoidosa
Compsopogon caeruleus
Erythrotrichia carnea
Durinskia baltica
Kryptoperidinium foliaceum
Plagiogramma staurophorum
Conticribra weissflogii
Thalassiosira pseudonana
Triparma laevis
Chrysochromulina parva
Emiliania huxleyi
Phaeocystis antarctica
Guillardia theta
Teleaulax amphioxeia
Cryptomonas curvata
Cyanophora biloba
Cyanophora sudae
Cyanophora paradoxa
Cyanoptyche gloeocystis
Glaucocystis incrassata
Glaucocystis sp BBH
Gloeochaete wittrockiana
Gloeomargarita lithophora Alchichica D10
Raphidiopsis curvata NIES 932
Sphaerospermopsis kisseleviana NIES 73
Anabaena cylindrica PCC 7122
Dolichospermum compactum NIES 806
Aulosira laxa NIES 50
Fremyella diplosiphon NIES 3275
Fischerella major NIES 592
Hapalosiphon sp MRB220
Richelia intracellularis HH01
Richelia intracellularis HM01
Calothrix parasitica NIES 267
Rivularia sp PCC 7116
Chroogloeocystis siderophila 52 sc1
Gloeocapsa sp PCC 7428
Aliterella atlantica CENA595
Pleurocapsa sp PCC 7327
Stanieria cyanosphaera PCC 7437
Arthrospira platensis NIES 39
Trichodesmium erythraeum IMS101
Oscillatoria acuminata PCC 6304
Paulinella micropora FK01
Paulinella micropora KR01
Paulinella micropora NZ27
Paulinella longichromatophora
Paulinella chromatophora
Cyanobium gracile PCC 6307
Synechococcus sp WH 5701
Synechococcus elongatus PCC 6301
Synechococcus sp JA 3 3Ab
Gloeobacter kilaueensis JS1
Streptophyta
Chlorophyta
Chlorarachnea
Chlorophyta
Streptophyta
Rhodophyta
Alveolata
Stramenopila
Haptophyta
Cryptophyta
Glaucophyta
Cyanobacteria
Paulinella
Cyanobacteria
Chloroplast
Chlorophyta derived plastid
Chloroplast
Rhodoplast
Rhodophyta derived plastid
Muroplast
Chromatophore
I
II
III
IV
V
VI
VII
VIII
IX
X
XI
Plp
Msp
Np
Pz
Mz
Cz
2379
1600
1000
542
251
65.5
Era

(See figure on the previous page) **Figure S11.** Time-calibrated phylogeny of photosynthetic organelles and cyanobacteria. The divergence times were inferred with PhyloBayes under UGAM model and calibrated with C1 (Tab. 1, S7). The tree topology was reconstructed in IQ-TREE. Other description as in Figure S1.

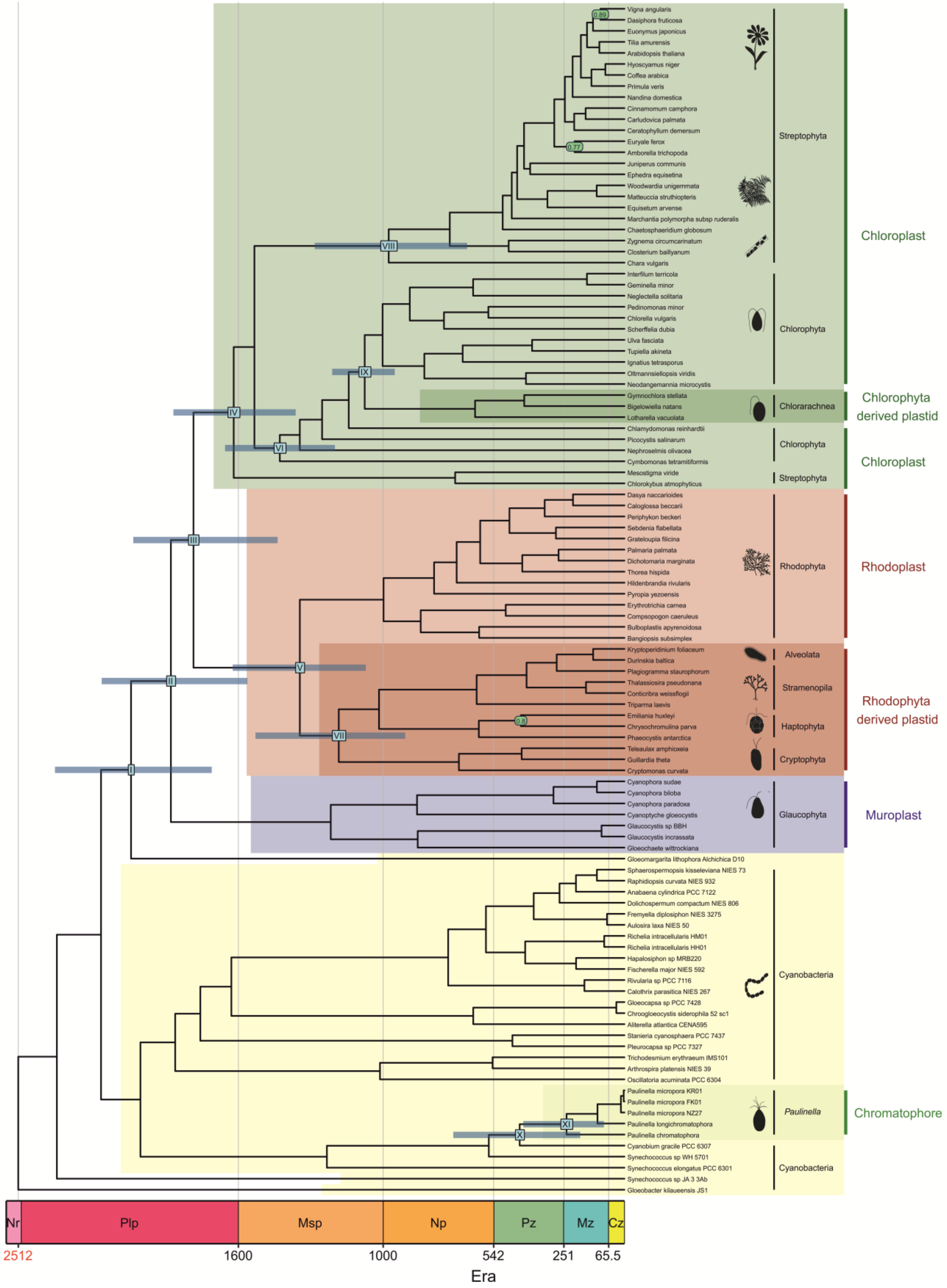
Vigna angularis
Dasiphora fruticosa
Euonymus japonicus
Tilia amurensis
Arabidopsis thaliana
Hyoscyamus niger
Coffea arabica
Primula veris
Nandina domestica
Cinnamomum camphora
Carludovica palmata
Ceratophyllum demersum
Euryale ferox
Amborella trichopoda
Juniperus communis
Ephedra equisetina
Woodwardia unigemmata
Matteuccia struthiopteris
Equisetum arvense
Marchantia polymorpha subsp ruderalis
Chaetosphaeridium globosum
Zygnema circumcarinatum
Closterium baillyanum
Chara vulgaris
Interfilum terricola
Geminella minor
Neglectella solitaria
Pedinomonas minor
Chlorella vulgaris
Scherffelia dubia
Ulva fasciata
Tupiella akineta
Ignatius tetrasporus
Oltmannsiellopsis viridis
Neodangemannia microcystis
Gymnochlora stellata
Bigelowiella natans
Lotharella vacuolata
Chlamydomonas reinhardtii
Picocystis salinarum
Nephroselmis olivacea
Cymbomonas tetramitiformis
Mesostigma viride
Chlorokybus atmophyticus
Dasya naccarioides
Caloglossa beccarii
Periphykon beckeri
Sebdenia flabellata
Grateloupia filicina
Palmaria palmata
Dichotomaria marginata
Thorea hispida
Hildenbrandia rivularis
Pyropia yezoensis
Erythrotrichia carnea
Compsopogon caeruleus
Bulboplastis apyrenoidosa
Bangiopsis subsimplex
Kryptoperidinium foliaceum
Durinskia baltica
Plagiogramma staurophorum
Thalassiosira pseudonana
Conticribra weissflogii
Triparma laevis
Emiliania huxleyi
Chrysochromulina parva
Phaeocystis antarctica
Teleaulax amphioxeia
Guillardia theta
Cryptomonas curvata
Cyanophora sudae
Cyanophora biloba
Cyanophora paradoxa
Cyanoptyche gloeocystis
Glaucocystis sp BBH
Glaucocystis incrassata
Gloeochaete wittrockiana
Gloeomargarita lithophora Alchichica D10
Sphaerospermopsis kisseleviana NIES 73
Raphidiopsis curvata NIES 932
Anabaena cylindrica PCC 7122
Dolichospermum compactum NIES 806
Fremyella diplosiphon NIES 3275
Aulosira laxa NIES 50
Richelia intracellularis HM01
Richelia intracellularis HH01
Hapalosiphon sp MRB220
Fischerella major NIES 592
Rivularia sp PCC 7116
Calothrix parasitica NIES 267
Gloeocapsa sp PCC 7428
Chroogloeocystis siderophila 52 sc1
Aliterella atlantica CENA595
Stanieria cyanosphaera PCC 7437
Pleurocapsa sp PCC 7327
Trichodesmium erythraeum IMS101
Arthrospira platensis NIES 39
Oscillatoria acuminata PCC 6304
Paulinella micropora KR01
Paulinella micropora FK01
Paulinella micropora NZ27
Paulinella longichromatophora
Paulinella chromatophora
Cyanobium gracile PCC 6307
Synechococcus sp WH 5701
Synechococcus elongatus PCC 6301
Synechococcus sp JA 3 3Ab
Gloeobacter kilaueensis JS1
0.89
0.77
0.8
I
II
III
IV
V
VI
VII
VIII
IX
X
XI
Streptophyta
Chlorophyta
Chlorarachnea
Chlorophyta
Streptophyta
Rhodophyta
Alveolata
Stramenopila
Haptophyta
Cryptophyta
Glaucophyta
Cyanobacteria
Paulinella
Cyanobacteria
Chloroplast
Chlorophyta derived plastid
Chloroplast
Rhodoplast
Rhodophyta derived plastid
Muroplast
Chromatophore
Nr
Plp
Msp
Np
Pz
Mz
Cz
2512
1600
1000
542
251
65.5
Era

(See figure on the previous page) **Figure S12.** Time-calibrated phylogeny of photosynthetic organelles and cyanobacteria. The tree was inferred with Beast under UCLNR model and calibrated with C2 (Tab. 1, S7). Other description as in Figure S1.

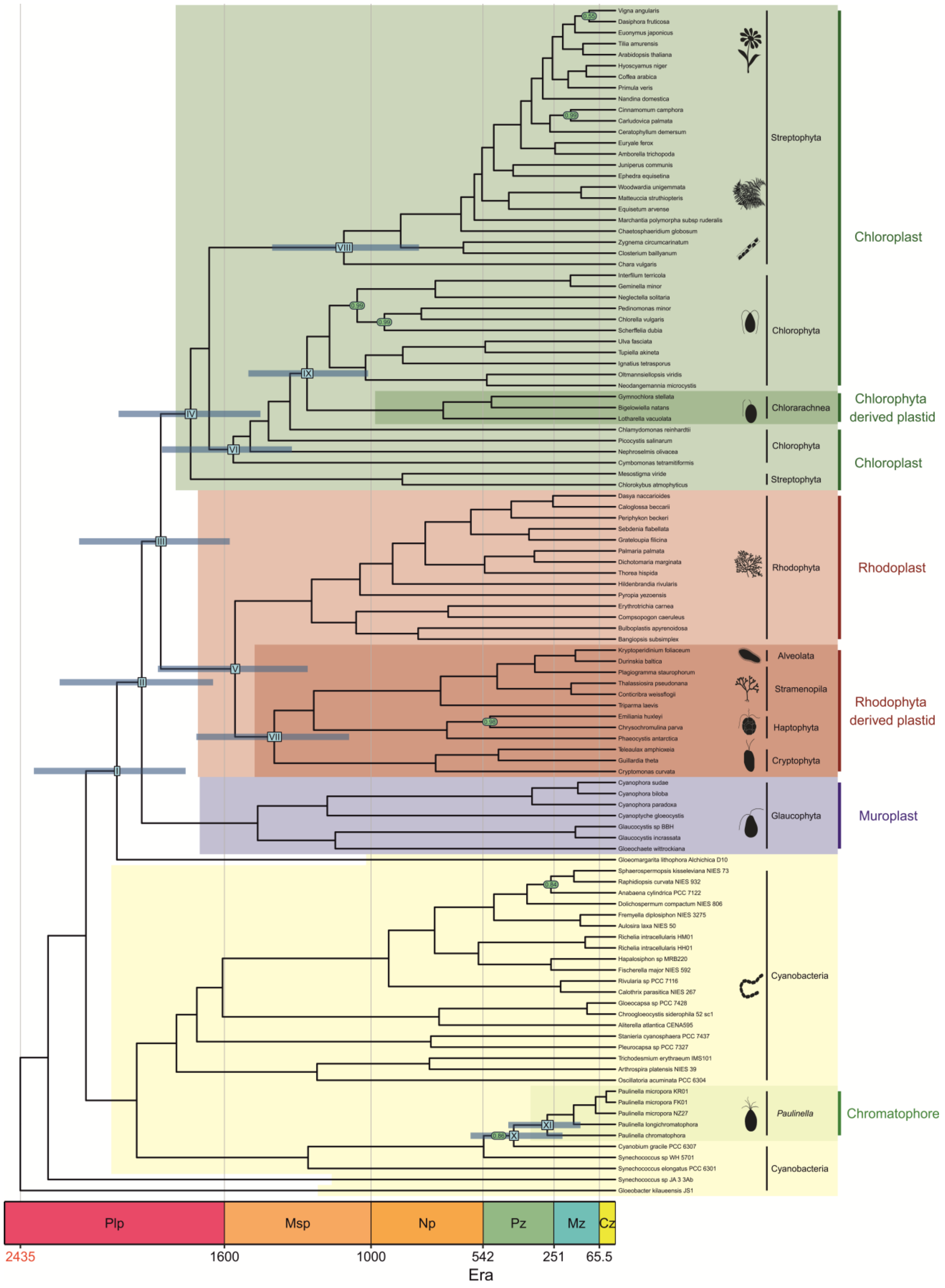

Vigna angularis
Dasiphora fruticosa
Euonymus japonicus
Tilia amurensis
Arabidopsis thaliana
Hyoscyamus niger
Coffea arabica
Primula veris
Nandina domestica
Cinnamomum camphora
Carludovica palmata
Ceratophyllum demersum
Euryale ferox
Amborella trichopoda
Juniperus communis
Ephedra equisetina
Woodwardia unigemmata
Matteuccia struthiopteris
Equisetum arvense
Marchantia polymorpha subsp ruderalis
Chaetosphaeridium globosum
Zygnema circumcarinatum
Closterium baillyanum
Chara vulgaris
Interfilum terricola
Geminella minor
Neglectella solitaria
Pedinomonas minor
Chlorella vulgaris
Scherffelia dubia
Ulva fasciata
Tupiella akineta
Ignatius tetrasporus
Oltmannsiellopsis viridis
Neodangemannia microcystis
Gymnochlora stellata
Bigelowiella natans
Lotharella vacuolata
Chlamydomonas reinhardtii
Picocystis salinarum
Nephroselmis olivacea
Cymbomonas tetramitiformis
Mesostigma viride
Chlorokybus atmophyticus
Dasya naccarioides
Caloglossa beccarii
Periphykon beckeri
Sebdenia flabellata
Grateloupia filicina
Palmaria palmata
Dichotomaria marginata
Thorea hispida
Hildenbrandia rivularis
Pyropia yezoensis
Erythrotrichia carnea
Compsopogon caeruleus
Bulboplastis apyrenoidosa
Bangiopsis subsimplex
Kryptoperidinium foliaceum
Durinskia baltica
Plagiogramma staurophorum
Thalassiosira pseudonana
Conticribra weissflogii
Triparma laevis
Emiliania huxleyi
Chrysochromulina parva
Phaeocystis antarctica
Teleaulax amphioxeia
Guillardia theta
Cryptomonas curvata
Cyanophora sudae
Cyanophora biloba
Cyanophora paradoxa
Cyanoptyche gloeocystis
Glaucocystis sp BBH
Glaucocystis incrassata
Gloeochaete wittrockiana
Gloeomargarita lithophora Alchichica D10
Sphaerospermopsis kisseleviana NIES 73
Raphidiopsis curvata NIES 932
Anabaena cylindrica PCC 7122
Dolichospermum compactum NIES 806
Fremyella diplosiphon NIES 3275
Aulosira laxa NIES 50
Richelia intracellularis HM01
Richelia intracellularis HH01
Hapalosiphon sp MRB220
Fischerella major NIES 592
Rivularia sp PCC 7116
Calothrix parasitica NIES 267
Gloeocapsa sp PCC 7428
Chroogloeocystis siderophila 52 sc1
Aliterella atlantica CENA595
Stanieria cyanosphaera PCC 7437
Pleurocapsa sp PCC 7327
Trichodesmium erythraeum IMS101
Arthrospira platensis NIES 39
Oscillatoria acuminata PCC 6304
Paulinella micropora KR01
Paulinella micropora FK01
Paulinella micropora NZ27
Paulinella longichromatophora
Paulinella chromatophora
Cyanobium gracile PCC 6307
Synechococcus sp WH 5701
Synechococcus elongatus PCC 6301
Synechococcus sp JA 3 3Ab
Gloeobacter kilaueensis JS1
Streptophyta
Chlorophyta
Chlorarachnea
Chlorophyta
Streptophyta
Rhodophyta
Alveolata
Stramenopila
Haptophyta
Cryptophyta
Glaucophyta
Cyanobacteria
Paulinella
Cyanobacteria
Chloroplast
Chlorophyta derived plastid
Chloroplast
Rhodoplast
Rhodophyta derived plastid
Muroplast
Chromatophore
I
II
III
IV
V
VI
VII
VIII
IX
X
XI
Plp
Msp
Np
Pz
Mz
Cz
2435
1600
1000
542
251
65.5
Era

(See figure on the previous page) **Figure S13.** Time-calibrated phylogeny of photosynthetic organelles and cyanobacteria. The tree was inferred with MrBayes under IGR model and calibrated with C2 (Tab. 1, S7). Other description as in Figure S1.

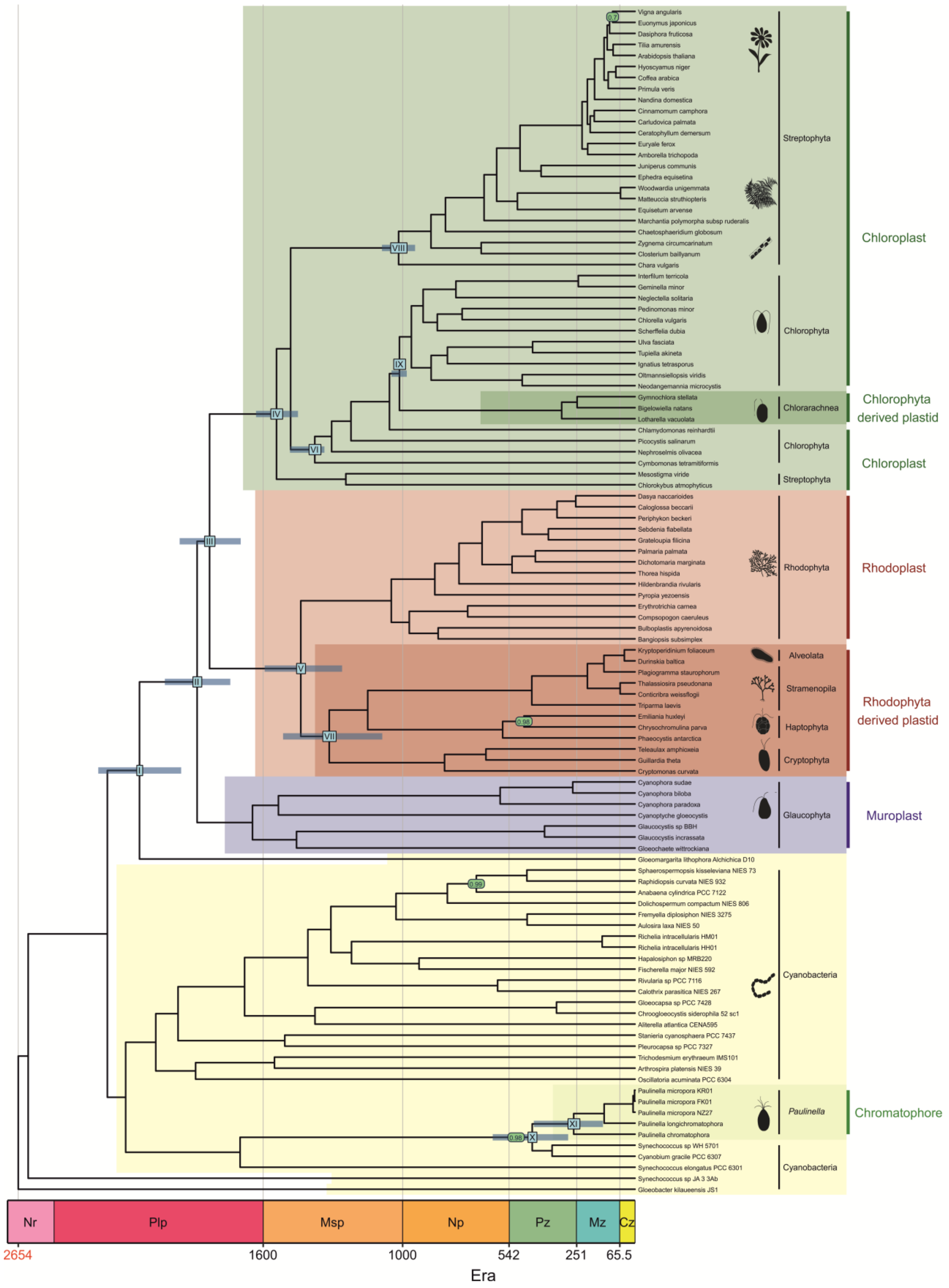
Vigna angularis
Euonymus japonicus
Dasiphora fruticosa
Tilia amurensis
Arabidopsis thaliana
Hyoscyamus niger
Coffea arabica
Primula veris
Nandina domestica
Cinnamomum camphora
Carludovica palmata
Ceratophyllum demersum
Euryale ferox
Amborella trichopoda
Juniperus communis
Ephedra equisetina
Woodwardia unigemmata
Matteuccia struthiopteris
Equisetum arvense
Marchantia polymorpha subsp ruderalis
Chaetosphaeridium globosum
Zygnema circumcarinatum
Closterium baillyanum
Chara vulgaris
Interfilum terricola
Geminella minor
Neglectella solitaria
Pedinomonas minor
Chlorella vulgaris
Scherffelia dubia
Ulva fasciata
Tupiella akineta
Ignatius tetrasporus
Oltmannsiellopsis viridis
Neodangemannia microcystis
Gymnochlora stellata
Bigelowiella natans
Lotharella vacuolata
Chlamydomonas reinhardtii
Picocystis salinarum
Nephroselmis olivacea
Cymbomonas tetramitiformis
Mesostigma viride
Chlorokybus atmophyticus
Dasya naccarioides
Caloglossa beccarii
Periphykon beckeri
Sebdenia flabellata
Grateloupia filicina
Palmaria palmata
Dichotomaria marginata
Thorea hispida
Hildenbrandia rivularis
Pyropia yezoensis
Erythrotrichia carnea
Compsopogon caeruleus
Bulboplastis apyrenoidosa
Bangiopsis subsimplex
Kryptoperidinium foliaceum
Durinskia baltica
Plagiogramma staurophorum
Thalassiosira pseudonana
Conticribra weissflogii
Triparma laevis
Emiliania huxleyi
Chrysochromulina parva
Phaeocystis antarctica
Teleaulax amphioxeia
Guillardia theta
Cryptomonas curvata
Cyanophora sudae
Cyanophora biloba
Cyanophora paradoxa
Cyanoptyche gloeocystis
Glaucocystis sp BBH
Glaucocystis incrassata
Gloeochaete wittrockiana
Gloeomargarita lithophora Alchichica D10
Sphaerospermopsis kisseleviana NIES 73
Raphidiopsis curvata NIES 932
Anabaena cylindrica PCC 7122
Dolichospermum compactum NIES 806
Fremyella diplosiphon NIES 3275
Aulosira laxa NIES 50
Richelia intracellularis HM01
Richelia intracellularis HH01
Hapalosiphon sp MRB220
Fischerella major NIES 592
Rivularia sp PCC 7116
Calothrix parasitica NIES 267
Gloeocapsa sp PCC 7428
Chroogloeocystis siderophila 52 sc1
Aliterella atlantica CENA595
Stanieria cyanosphaera PCC 7437
Pleurocapsa sp PCC 7327
Trichodesmium erythraeum IMS101
Arthrospira platensis NIES 39
Oscillatoria acuminata PCC 6304
Paulinella micropora KR01
Paulinella micropora FK01
Paulinella micropora NZ27
Paulinella longichromatophora
Paulinella chromatophora
Synechococcus sp WH 5701
Cyanobium gracile PCC 6307
Synechococcus elongatus PCC 6301
Synechococcus sp JA 3 3Ab
Gloeobacter kilaueensis JS1
0.7
0.98
0.99
0.98
I
II
III
IV
V
VI
VII
VIII
IX
X
XI
Streptophyta
Chlorophyta
Chlorarachnea
Chlorophyta
Streptophyta
Rhodophyta
Alveolata
Stramenopila
Haptophyta
Cryptophyta
Glaucophyta
Cyanobacteria
Paulinella
Cyanobacteria
Chloroplast
Chlorophyta derived plastid
Chloroplast
Rhodoplast
Rhodophyta derived plastid
Muroplast
Chromatophore
Nr
Plp
Msp
Np
Pz
Mz
Cz
2654
1600
1000
542
251
65.5
Era

(See figure on the previous page) **Figure S14.** Time-calibrated phylogeny of photosynthetic organelles and cyanobacteria. The tree was inferred with MrBayes under TK02 model and calibrated with C2 (Tab. 1, S7). Other description as in Figure S1.

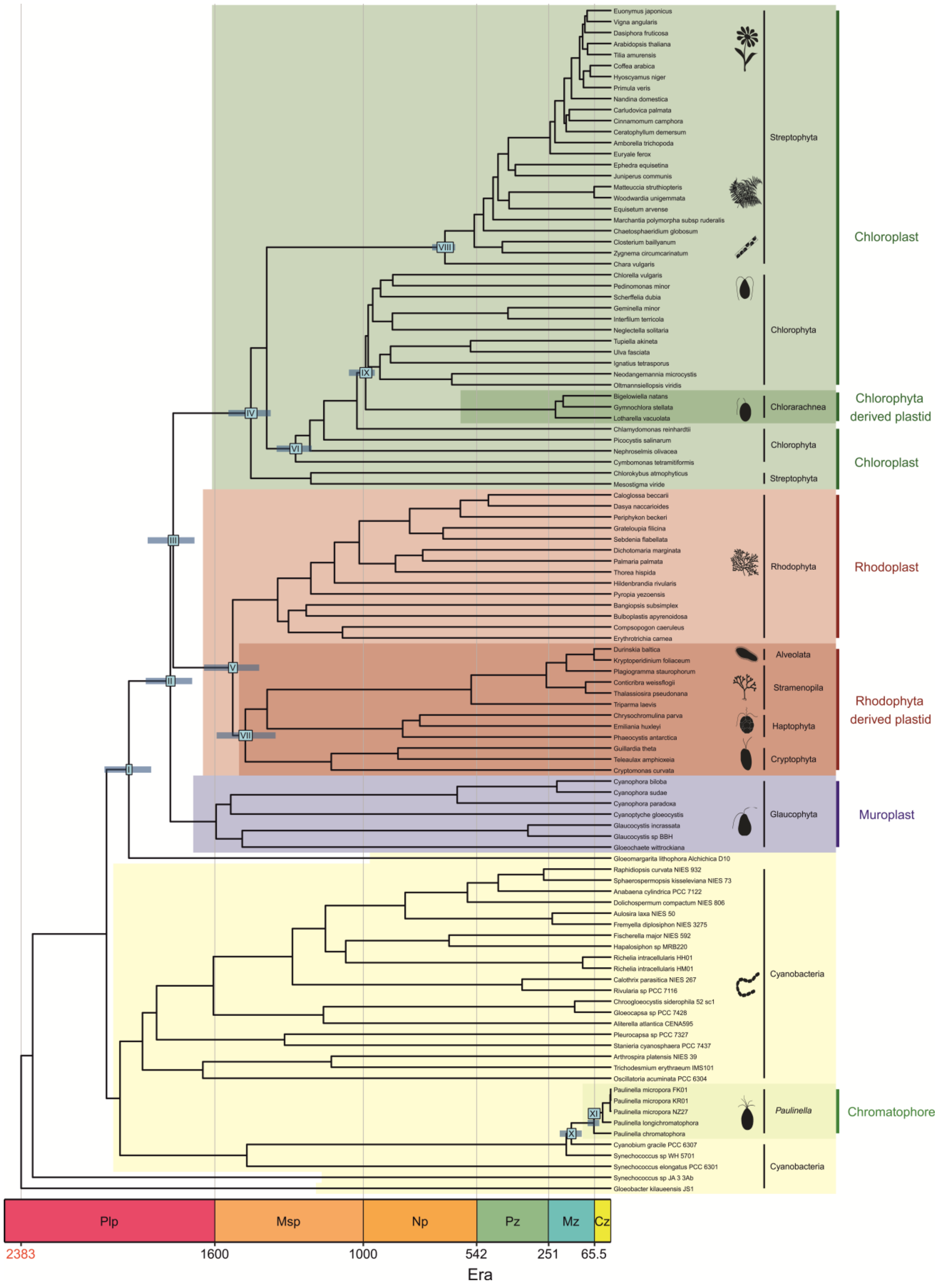
Euonymus japonicus
Vigna angularis
Dasiphora fruticosa
Arabidopsis thaliana
Tilia amurensis
Coffea arabica
Hyoscyamus niger
Primula veris
Nandina domestica
Carludovica palmata
Cinnamomum camphora
Ceratophyllum demersum
Amborella trichopoda
Euryale ferox
Ephedra equisetina
Juniperus communis
Matteuccia struthiopteris
Woodwardia unigemmata
Equisetum arvense
Marchantia polymorpha subsp ruderalis
Chaetosphaeridium globosum
Closterium baillyanum
Zygnema circumcarinatum
Chara vulgaris
Chlorella vulgaris
Pedinomonas minor
Scherffelia dubia
Geminella minor
Interfilum terricola
Neglectella solitaria
Tupiella akineta
Ulva fasciata
Ignatius tetrasporus
Neodangemannia microcystis
Oltmannsiellopsis viridis
Bigelowiella natans
Gymnochlora stellata
Lotharella vacuolata
Chlamydomonas reinhardtii
Picocystis salinarum
Nephroselmis olivacea
Cymbomonas tetramitiformis
Chlorokybus atmophyticus
Mesostigma viride
Caloglossa beccarii
Dasya naccarioides
Periphykon beckeri
Grateloupia filicina
Sebdenia flabellata
Dichotomaria marginata
Palmaria palmata
Thorea hispida
Hildenbrandia rivularis
Pyropia yezoensis
Bangiopsis subsimplex
Bulboplastis apyrenoidosa
Compsopogon caeruleus
Erythrotrichia camea
Durinskia baltica
Kryptoperidinium foliaceum
Plagiogramma staurophorum
Conticribra weissflogii
Thalassiosira pseudonana
Triparma laevis
Chrysochromulina parva
Emiliania huxleyi
Phaeocystis antarctica
Guillardia theta
Teleaulax amphioxeia
Cryptomonas curvata
Cyanophora biloba
Cyanophora sudae
Cyanophora paradoxa
Cyanoptyche gloeocystis
Glaucocystis incrassata
Glaucocystis sp BBH
Gloeochaete wittrockiana
Gloeomargarita lithophora Alchichica D10
Raphidiopsis curvata NIES 932
Sphaerospermopsis kisseleviana NIES 73
Anabaena cylindrica PCC 7122
Dolichospermum compactum NIES 806
Aulosira laxa NIES 50
Fremyella diplosiphon NIES 3275
Fischerella major NIES 592
Hapalosiphon sp MRB220
Richelia intracellularis HH01
Richelia intracellularis HM01
Calothrix parasitica NIES 267
Rivularia sp PCC 7116
Chroogloeocystis siderophila 52 sc1
Gloeocapsa sp PCC 7428
Aliterella atlantica CENA595
Pleurocapsa sp PCC 7327
Stanieria cyanosphaera PCC 7437
Arthrospira platensis NIES 39
Trichodesmium erythraeum IMS101
Oscillatoria acuminata PCC 6304
Paulinella micropora FK01
Paulinella micropora KR01
Paulinella micropora NZ27
Paulinella longichromatophora
Paulinella chromatophora
Cyanobium gracile PCC 6307
Synechococcus sp WH 5701
Synechococcus elongatus PCC 6301
Synechococcus sp JA 3 3Ab
Gloeobacter kilaueensis JS1
Streptophyta
Chlorophyta
Chlorarachnea
Chlorophyta
Streptophyta
Rhodophyta
Alveolata
Stramenopila
Haptophyta
Cryptophyta
Glaucophyta
Cyanobacteria
Paulinella
Cyanobacteria
Chloroplast
Chlorophyta derived plastid
Chloroplast
Rhodoplast
Rhodophyta derived plastid
Muroplast
Chromatophore
I
II
III
IV
V
VI
VII
VIII
IX
X
XI
Plp
Msp
Np
Pz
Mz
Cz
2383
1600
1000
542
251
65.5
Era

(See figure on the previous page) **Figure S15.** Time-calibrated phylogeny of photosynthetic organelles and cyanobacteria. The divergence times were inferred with PhyloBayes under CIR model and calibrated with C2 (Tab. 1, S7). The tree topology was reconstructed in IQ-TREE. Other description as in Figure S1.

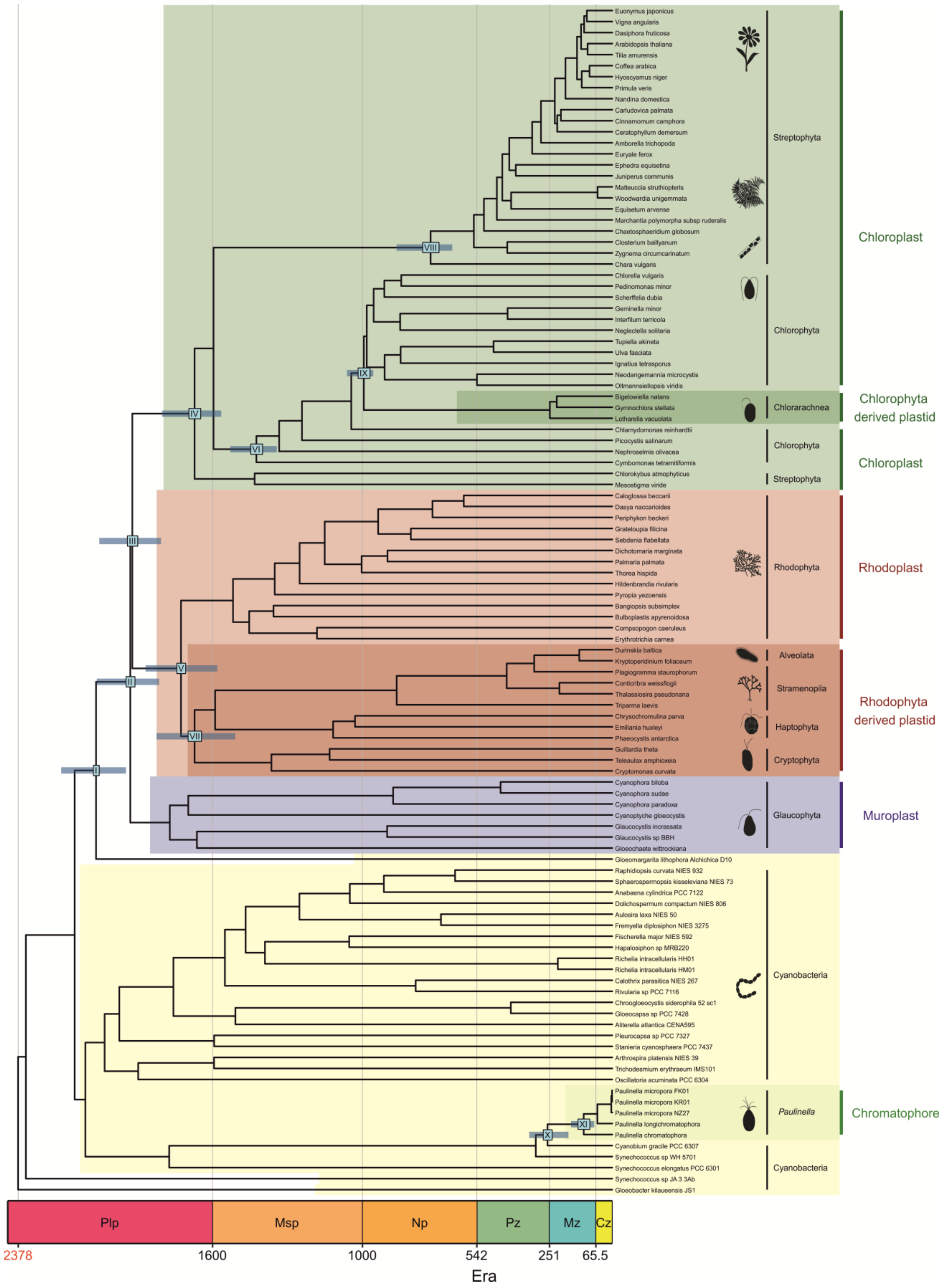
Euonymus japonicus
Vigna angularis
Dasiphora fruticosa
Arabidopsis thaliana
Tilia amurensis
Coffea arabica
Hyoscyamus niger
Primula veris
Nandina domestica
Carludovica palmata
Cinnamomum camphora
Ceratophyllum demersum
Amborella trichopoda
Euryale ferox
Ephedra equisetina
Juniperus communis
Matteuccia struthiopteris
Woodwardia unigemmata
Equisetum arvense
Marchantia polymorpha subsp ruderalis
Chaetosphaeridium globosum
Closterium baillyanum
Zygnema circumcarinatum
Chara vulgaris
Chlorella vulgaris
Pedinomonas minor
Scherffelia dubia
Geminella minor
Interfilum terricola
Neglectella solitaria
Tupiella akineta
Ulva fasciata
Ignatius tetrasporus
Neodangemannia microcystis
Oltmannsiellopsis viridis
Bigelowiella natans
Gymnochlora stellata
Lotharella vacuolata
Chlamydomonas reinhardtii
Picocystis salinarum
Nephroselmis olivacea
Cymbomonas tetramitiformis
Chlorokybus atmophyticus
Mesostigma viride
Caloglossa beccarii
Dasya naccarioides
Periphykon beckeri
Grateloupia filicina
Sebdenia flabellata
Dichotomaria marginata
Palmaria palmata
Thorea hispida
Hildenbrandia rivularis
Pyropia yezoensis
Bangiopsis subsimplex
Bulboplastis apyrenoidosa
Compsopogon caeruleus
Erythrotrichia carnea
Durinskia baltica
Kryptoperidinium foliaceum
Plagiogramma staurophorum
Conticribra weissflogii
Thalassiosira pseudonana
Triparma laevis
Chrysochromulina parva
Emiliania huxleyi
Phaeocystis antarctica
Guillardia theta
Teleaulax amphioxeia
Cryptomonas curvata
Cyanophora biloba
Cyanophora sudae
Cyanophora paradoxa
Cyanoptyche gloeocystis
Glaucocystis incrassata
Glaucocystis sp BBH
Gloeochaete wittrockiana
Gloeomargarita lithophora Alchichica D10
Raphidiopsis curvata NIES 932
Sphaerospermopsis kisseleviana NIES 73
Anabaena cylindrica PCC 7122
Dolichospermum compactum NIES 806
Aulosira laxa NIES 50
Fremyella diplosiphon NIES 3275
Fischerella major NIES 592
Hapalosiphon sp MRB220
Richelia intracellularis HH01
Richelia intracellularis HM01
Calothrix parasitica NIES 267
Rivularia sp PCC 7116
Chroogloeocystis siderophila 52 sc1
Gloeocapsa sp PCC 7428
Aliterella atlantica CENA595
Pleurocapsa sp PCC 7327
Stanieria cyanosphaera PCC 7437
Arthrospira platensis NIES 39
Trichodesmium erythraeum IMS101
Oscillatoria acuminata PCC 6304
Paulinella micropora FK01
Paulinella micropora KR01
Paulinella micropora NZ27
Paulinella longichromatophora
Paulinella chromatophora
Cyanobium gracile PCC 6307
Synechococcus sp WH 5701
Synechococcus elongatus PCC 6301
Synechococcus sp JA 3 3Ab
Gloeobacter kilaueensis JS1
Streptophyta
Chlorophyta
Chlorarachnea
Chlorophyta
Streptophyta
Rhodophyta
Alveolata
Stramenopila
Haptophyta
Cryptophyta
Glaucophyta
Cyanobacteria
Paulinella
Cyanobacteria
Chloroplast
Chlorophyta derived plastid
Chloroplast
Rhodoplast
Rhodophyta derived plastid
Muroplast
Chromatophore
I
II
III
IV
V
VI
VII
VIII
IX
X
XI
Plp
Msp
Np
Pz
Mz
Cz
2378
1600
1000
542
251
65.5
Era

(See figure on the previous page) **Figure S16.** Time-calibrated phylogeny of photosynthetic organelles and cyanobacteria. The divergence times were inferred with PhyloBayes under LN model and calibrated with C2 (Tab. 1, S7). The tree topology was reconstructed in IQ-TREE. Other description as in Figure S1.

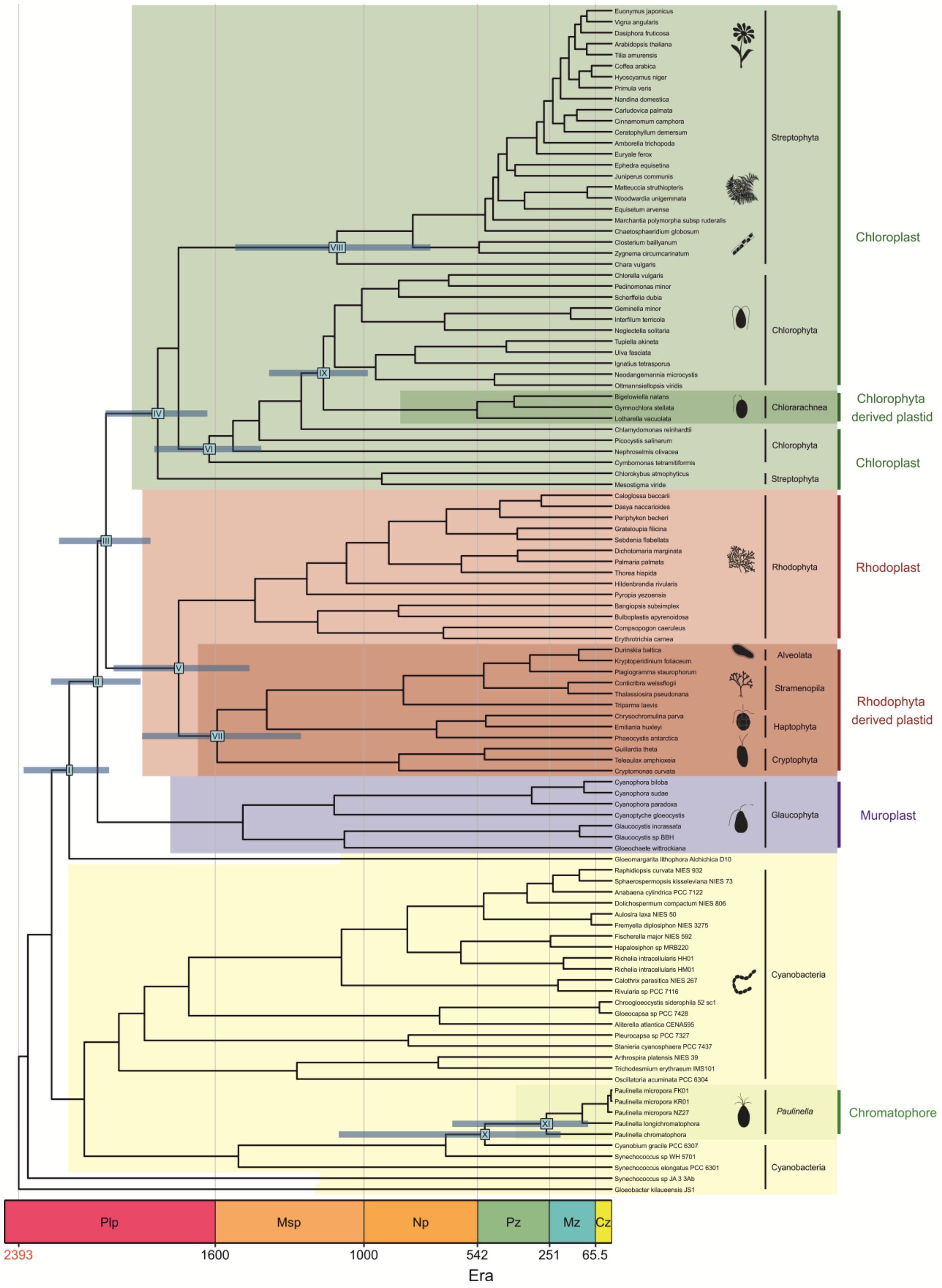
Euonymus japonicus
Vigna angularis
Dasiphora fruticosa
Arabidopsis thaliana
Tilia amurensis
Coffea arabica
Hyoscyamus niger
Primula veris
Nandina domestica
Carludovica palmata
Cinnamomum camphora
Ceratophyllum demersum
Amborella trichopoda
Euryale ferox
Ephedra equisetina
Juniperus communis
Matteuccia struthiopteris
Woodwardia unigemmata
Equisetum arvense
Marchantia polymorpha subsp ruderalis
Chaetosphaeridium globosum
Closterium baillyanum
Zygnema circumcarinatum
Chara vulgaris
Chlorella vulgaris
Pedinomonas minor
Scherffelia dubia
Geminella minor
Interfilum terricola
Neglectella solitaria
Tupiella akineta
Ulva fasciata
Ignatius tetrasporus
Neodangemannia microcystis
Oltmannsiellopsis viridis
Bigelowiella natans
Gymnochlora stellata
Lotharella vacuolata
Chlamydomonas reinhardtii
Picocystis salinarum
Nephroselmis olivacea
Cymbomonas tetramitiformis
Chlorokybus atmophyticus
Mesostigma viride
Caloglossa beccarii
Dasya naccarioides
Periphykon beckeri
Grateloupia filicina
Sebdenia flabellata
Dichotomaria marginata
Palmaria palmata
Thorea hispida
Hildenbrandia rivularis
Pyropia yezoensis
Bangiopsis subsimplex
Bulboplastis apyrenoidosa
Compsopogon caeruleus
Erythrotrichia carnea
Durinskia baltica
Kryptoperidinium foliaceum
Plagiogramma staurophorum
Conticribra weissflogii
Thalassiosira pseudonana
Triparma laevis
Chrysochromulina parva
Emiliania huxleyi
Phaeocystis antarctica
Guillardia theta
Teleaulax amphioxeia
Cryptomonas curvata
Cyanophora biloba
Cyanophora sudae
Cyanophora paradoxa
Cyanoptyche gloeocystis
Glaucocystis incrassata
Glaucocystis sp BBH
Gloeochaete wittrockiana
Gloeomargarita lithophora Alchichica D10
Raphidiopsis curvata NIES 932
Sphaerospermopsis kisseleviana NIES 73
Anabaena cylindrica PCC 7122
Dolichospermum compactum NIES 806
Aulosira laxa NIES 50
Fremyella diplosiphon NIES 3275
Fischerella major NIES 592
Hapalosiphon sp MRB220
Richelia intracellularis HH01
Richelia intracellularis HM01
Calothrix parasitica NIES 267
Rivularia sp PCC 7116
Chroogloeocystis siderophila 52 sc1
Gloeocapsa sp PCC 7428
Aliterella atlantica CENA595
Pleurocapsa sp PCC 7327
Stanieria cyanosphaera PCC 7437
Arthrospira platensis NIES 39
Trichodesmium erythraeum IMS101
Oscillatoria acuminata PCC 6304
Paulinella micropora FK01
Paulinella micropora KR01
Paulinella micropora NZ27
Paulinella longichromatophora
Paulinella chromatophora
Cyanobium gracile PCC 6307
Synechococcus sp WH 5701
Synechococcus elongatus PCC 6301
Synechococcus sp JA 3 3Ab
Gloeobacter kilaueensis JS1
Streptophyta
Chlorophyta
Chlorarachnea
Chlorophyta
Streptophyta
Rhodophyta
Alveolata
Stramenopila
Haptophyta
Cryptophyta
Glaucophyta
Cyanobacteria
Paulinella
Cyanobacteria
Chloroplast
Chlorophyta derived plastid
Chloroplast
Rhodoplast
Rhodophyta derived plastid
Muroplast
Chromatophore
I
II
III
IV
V
VI
VII
VIII
IX
X
XI
Plp
Msp
Np
Pz
Mz
Cz
2393
1600
1000
542
251
65.5
Era

(See figure on the previous page) **Figure S17.** Time-calibrated phylogeny of photosynthetic organelles and cyanobacteria. The divergence times were inferred with PhyloBayes under UGAM model and calibrated with C2 (Tab. 1, S7). The tree topology was reconstructed in IQ-TREE. Other description as in Figure S1.

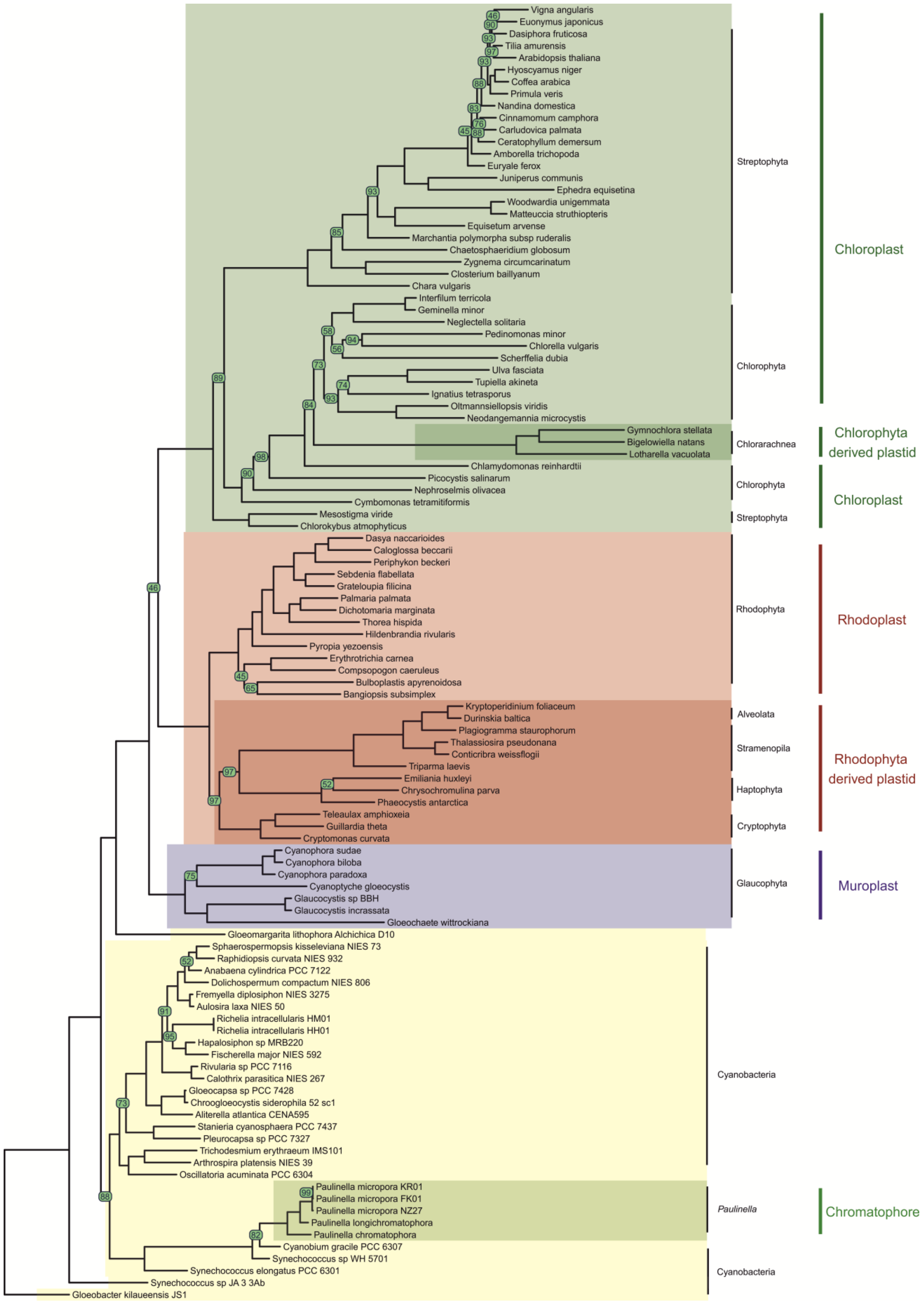

Vigna angularis
Euonymus japonicus
Dasiphora fruticosa
Tilia amurensis
Arabidopsis thaliana
Hyoscyamus niger
Coffea arabica
Primula veris
Nandina domestica
Cinnamomum camphora
Carludovica palmata
Ceratophyllum demersum
Amborella trichopoda
Euryale ferox
Juniperus communis
Ephedra equisetina
Woodwardia unigemmata
Matteuccia struthiopteris
Equisetum arvense
Marchantia polymorpha subsp ruderalis
Chaetosphaeridium globosum
Zygnema circumcarinatum
Closterium baillyanum
Chara vulgaris
Interfilum terricola
Geminella minor
Neglectella solitaria
Pedinomonas minor
Chlorella vulgaris
Scherffelia dubia
Ulva fasciata
Tupiella akineta
Ignatius tetrasporus
Oltmannsiellopsis viridis
Neodangemannia microcystis
Gymnochlora stellata
Bigelowiella natans
Lotharella vacuolata
Chlamydomonas reinhardtii
Picocystis salinarum
Nephroselmis olivacea
Cymbomonas tetramitiformis
Mesostigma viride
Chlorokybus atmophyticus
Dasya naccarioides
Caloglossa beccarii
Periphykon beckeri
Sebdenia flabellata
Grateloupia filicina
Palmaria palmata
Dichotomaria marginata
Thorea hispida
Hildenbrandia rivularis
Pyropia yezoensis
Erythrotrichia carnea
Compsopogon caeruleus
Bulboplastis apyrenoidosa
Bangiopsis subsimplex
Kryptoperidinium foliaceum
Durinskia baltica
Plagiogramma staurophorum
Thalassiosira pseudonana
Conticribra weissflogii
Triparma laevis
Emiliania huxleyi
Chrysochromulina parva
Phaeocystis antarctica
Teleaulax amphioxeia
Guillardia theta
Cryptomonas curvata
Cyanophora sudae
Cyanophora biloba
Cyanophora paradoxa
Cyanoptyche gloeocystis
Glaucocystis sp BBH
Glaucocystis incrassata
Gloeochaete wittrockiana
Gloeomargarita lithophora Alchichica D10
Sphaerospermopsis kisseleviana NIES 73
Raphidiopsis curvata NIES 932
Anabaena cylindrica PCC 7122
Dolichospermum compactum NIES 806
Fremyella diplosiphon NIES 3275
Aulosira laxa NIES 50
Richelia intracellularis HM01
Richelia intracellularis HH01
Hapalosiphon sp MRB220
Fischerella major NIES 592
Rivularia sp PCC 7116
Calothrix parasitica NIES 267
Gloeocapsa sp PCC 7428
Chroogloeocystis siderophila 52 sc1
Aliterella atlantica CENA595
Stanieria cyanosphaera PCC 7437
Pleurocapsa sp PCC 7327
Trichodesmium erythraeum IMS101
Arthrospira platensis NIES 39
Oscillatoria acuminata PCC 6304
Paulinella micropora KR01
Paulinella micropora FK01
Paulinella micropora NZ27
Paulinella longichromatophora
Paulinella chromatophora
Cyanobium gracile PCC 6307
Synechococcus sp WH 5701
Synechococcus elongatus PCC 6301
Synechococcus sp JA 3 3Ab
Gloeobacter kilaueensis JS1
Streptophyta
Chlorophyta
Chlorarachnea
Chlorophyta
Streptophyta
Rhodophyta
Alveolata
Stramenopila
Haptophyta
Cryptophyta
Glaucophyta
Cyanobacteria
Paulinella
Cyanobacteria
Chloroplast
Chlorophyta derived plastid
Chloroplast
Rhodoplast
Rhodophyta derived plastid
Muroplast
Chromatophore

(See figure on the previous page) **Figure S18.** The phylogeny of photosynthetic organelles and cyanobacteria. The tree topology was reconstructed in IQ-TREE. The nodes supported with bootstrap values lower than 100 are indicated in green circles.

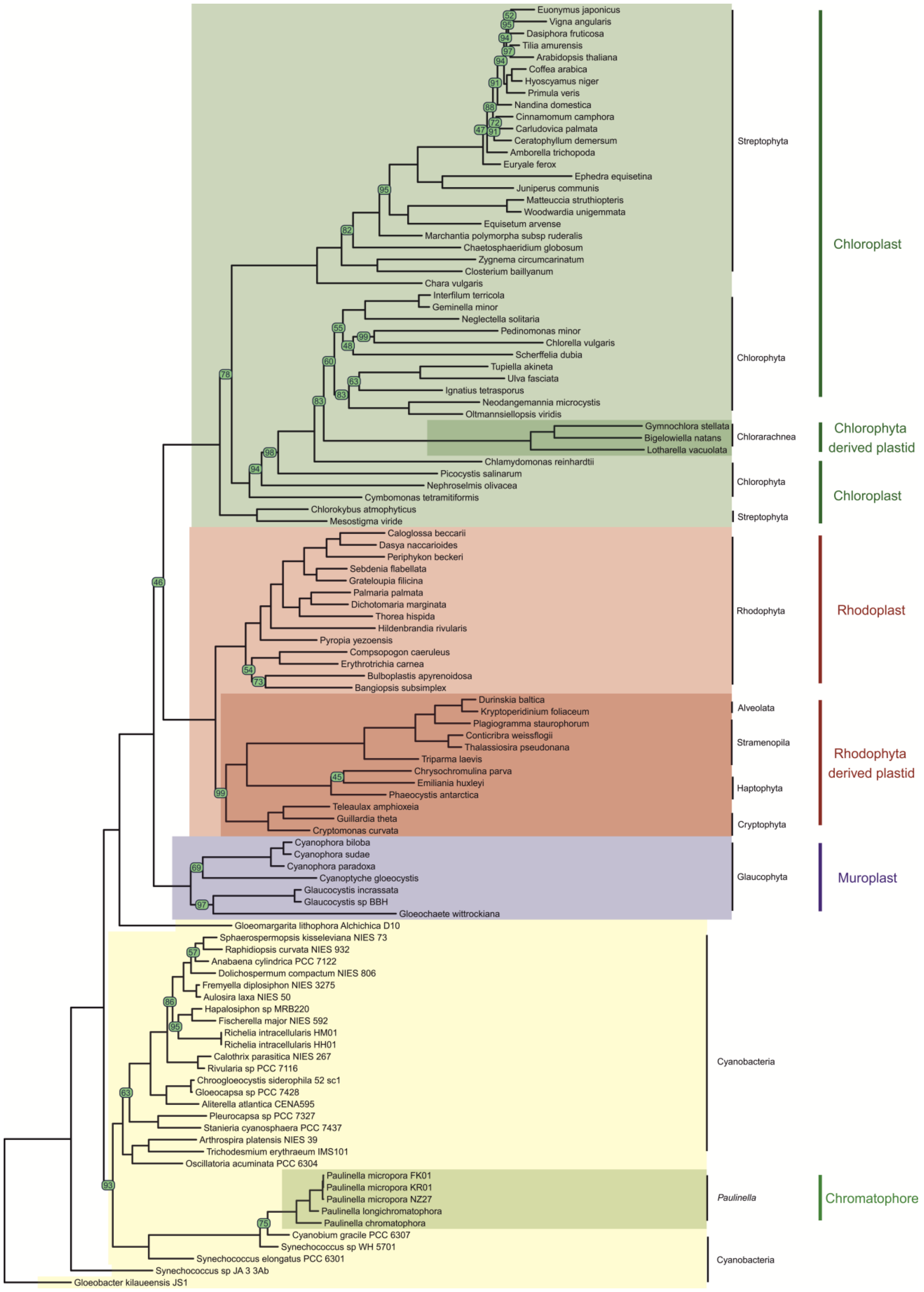
Euonymus japonicus
Vigna angularis
Dasiphora fruticosa
Tilia amurensis
Arabidopsis thaliana
Coffea arabica
Hyoscyamus niger
Primula veris
Nandina domestica
Cinnamomum camphora
Carludovica palmata
Ceratophyllum demersum
Amborella trichopoda
Euryale ferox
Ephedra equisetina
Juniperus communis
Matteuccia struthiopteris
Woodwardia unigemmata
Equisetum arvense
Marchantia polymorpha subsp ruderalis
Chaetosphaeridium globosum
Zygnema circumcarinatum
Closterium baillyanum
Chara vulgaris
Interfilum terricola
Geminella minor
Neglectella solitaria
Pedinomonas minor
Chlorella vulgaris
Scherffelia dubia
Tupiella akineta
Ulva fasciata
Ignatius tetrasporus
Neodangemannia microcystis
Oltmannsiellopsis viridis
Gymnochlora stellata
Bigelowiella natans
Lotharella vacuolata
Chlamydomonas reinhardtii
Picocystis salinarum
Nephroselmis olivacea
Cymbomonas tetramitiformis
Chlorokybus atmophyticus
Mesostigma viride
Caloglossa beccarii
Dasya naccarioides
Periphykon beckeri
Sebdenia flabellata
Grateloupia filicina
Palmaria palmata
Dichotomaria marginata
Thorea hispida
Hildenbrandia rivularis
Pyropia yezoensis
Compsopogon caeruleus
Erythrotrichia carnea
Bulboplastis apyrenoidosa
Bangiopsis subsimplex
Durinskia baltica
Kryptoperidinium foliaceum
Plagiogramma staurophorum
Conticribra weissflogii
Thalassiosira pseudonana
Triparma laevis
Chrysochromulina parva
Emiliania huxleyi
Phaeocystis antarctica
Teleaulax amphioxeia
Guillardia theta
Cryptomonas curvata
Cyanophora biloba
Cyanophora sudae
Cyanophora paradoxa
Cyanoptyche gloeocystis
Glaucocystis incrassata
Glaucocystis sp BBH
Gloeochaete wittrockiana
Gloeomargarita lithophora Alchichica D10
Sphaerospermopsis kisseleviana NIES 73
Raphidiopsis curvata NIES 932
Anabaena cylindrica PCC 7122
Dolichospermum compactum NIES 806
Fremyella diplosiphon NIES 3275
Aulosira laxa NIES 50
Hapalosiphon sp MRB220
Fischerella major NIES 592
Richelia intracellularis HM01
Richelia intracellularis HH01
Calothrix parasitica NIES 267
Rivularia sp PCC 7116
Chroogloeocystis siderophila 52 sc1
Gloeocapsa sp PCC 7428
Aliterella atlantica CENA595
Pleurocapsa sp PCC 7327
Stanieria cyanosphaera PCC 7437
Arthrospira platensis NIES 39
Trichodesmium erythraeum IMS101
Oscillatoria acuminata PCC 6304
Paulinella micropora FK01
Paulinella micropora KR01
Paulinella micropora NZ27
Paulinella longichromatophora
Paulinella chromatophora
Cyanobium gracile PCC 6307
Synechococcus sp WH 5701
Synechococcus elongatus PCC 6301
Synechococcus sp JA 3 3Ab
Gloeobacter kilaueensis JS1
Streptophyta
Chlorophyta
Chlorarachnea
Chlorophyta
Streptophyta
Rhodophyta
Alveolata
Stramenopila
Haptophyta
Cryptophyta
Glaucophyta
Cyanobacteria
Paulinella
Cyanobacteria
Chloroplast
Chlorophyta derived plastid
Chloroplast
Rhodoplast
Rhodophyta derived plastid
Muroplast
Chromatophore

(See figure on the previous page) **Figure S19.** The phylogeny of photosynthetic organelles and cyanobacteria. The tree topology was reconstructed in RAxML. The nodes supported with bootstrap values lower than 100 are indicated in green circles.

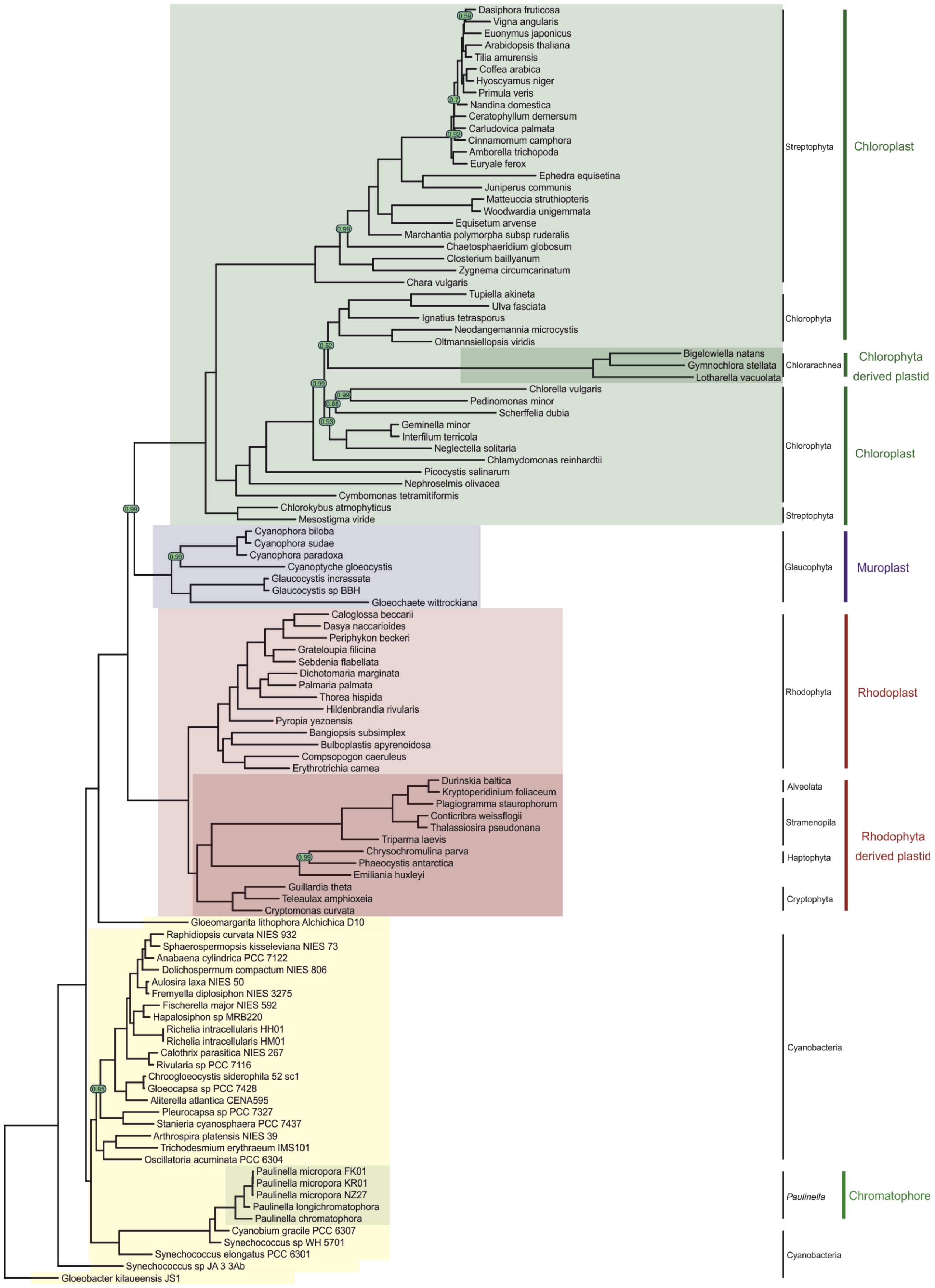

Dasiphora fruticosa
0.59
Vigna angularis
Euonymus japonicus
Arabidopsis thaliana
Tilia amurensis
Coffea arabica
Hyoscyamus niger
Primula veris
0.7
Nandina domestica
Ceratophyllum demersum
Carludovica palmata
0.92
Cinnamomum camphora
Amborella trichopoda
Euryale ferox
Ephedra equisetina
Juniperus communis
Matteuccia struthiopteris
Woodwardia unigemmata
Equisetum arvense
0.99
Marchantia polymorpha subsp ruderalis
Chaetosphaeridium globosum
Closterium baillyanum
Zygnema circumcarinatum
Chara vulgaris
Tupiella akineta
Ulva fasciata
Ignatius tetrasporus
Neodangemannia microcystis
Oltmannsiellopsis viridis
0.52
Bigelowiella natans
Gymnochlora stellata
Lotharella vacuolata
0.99
Chlorella vulgaris
0.99
0.88
Pedinomonas minor
Scherffelia dubia
0.93
Geminella minor
Interfilum terricola
Neglectella solitaria
Chlamydomonas reinhardtii
Picocystis salinarum
Nephroselmis olivacea
Cymbomonas tetramitiformis
0.99
Chlorokybus atmophyticus
Mesostigma viride
Cyanophora biloba
Cyanophora sudae
0.99
Cyanophora paradoxa
Cyanoptyche gloeocystis
Glaucocystis incrassata
Glaucocystis sp BBH
Gloeochaete wittrockiana
Caloglossa beccarii
Dasya naccarioides
Periphykon beckeri
Grateloupia filicina
Sebdenia flabellata
Dichotomaria marginata
Palmaria palmata
Thorea hispida
Hildenbrandia rivularis
Pyropia yezoensis
Bangiopsis subsimplex
Bulboplastis apyrenoidosa
Compsopogon caeruleus
Erythrotrichia carnea
Durinskia baltica
Kryptoperidinium foliaceum
Plagiogramma staurophorum
Conticribra weissflogii
Thalassiosira pseudonana
Triparma laevis
0.99
Chrysochromulina parva
Phaeocystis antarctica
Emiliania huxleyi
Guillardia theta
Teleaulax amphioxeia
Cryptomonas curvata
Gloeomargarita lithophora Alchichica D10
Raphidiopsis curvata NIES 932
Sphaerospermopsis kisseleviana NIES 73
Anabaena cylindrica PCC 7122
Dolichospermum compactum NIES 806
Aulosira laxa NIES 50
Fremyella diplosiphon NIES 3275
Fischerella major NIES 592
Hapalosiphon sp MRB220
Richelia intracellularis HH01
Richelia intracellularis HM01
Calothrix parasitica NIES 267
Rivularia sp PCC 7116
Chroogloeocystis siderophila 52 sc1
0.95
Gloeocapsa sp PCC 7428
Aliterella atlantica CENA595
Pleurocapsa sp PCC 7327
Stanieria cyanosphaera PCC 7437
Arthrospira platensis NIES 39
Trichodesmium erythraeum IMS101
Oscillatoria acuminata PCC 6304
Paulinella micropora FK01
Paulinella micropora KR01
Paulinella micropora NZ27
Paulinella longichromatophora
Paulinella chromatophora
Cyanobium gracile PCC 6307
Synechococcus sp WH 5701
Synechococcus elongatus PCC 6301
Synechococcus sp JA 3 3Ab
Gloeobacter kilaueensis JS1
Streptophyta
Chlorophyta
Chlorarachnea
Chlorophyta
Streptophyta
Glaucophyta
Rhodophyta
Alveolata
Stramenopila
Haptophyta
Cryptophyta
Cyanobacteria
Paulinella
Cyanobacteria
Chloroplast
Chlorophyta derived plastid
Chloroplast
Muroplast
Rhodoplast
Rhodophyta derived plastid
Chromatophore

(See figure on the previous page) **Figure S20.** The phylogeny of photosynthetic organelles and cyanobacteria. The tree topology was reconstructed in PhyloBayes under CAT-GTR substitution model. The nodes supported with posterior values lower than 1 are indicated in green circles.

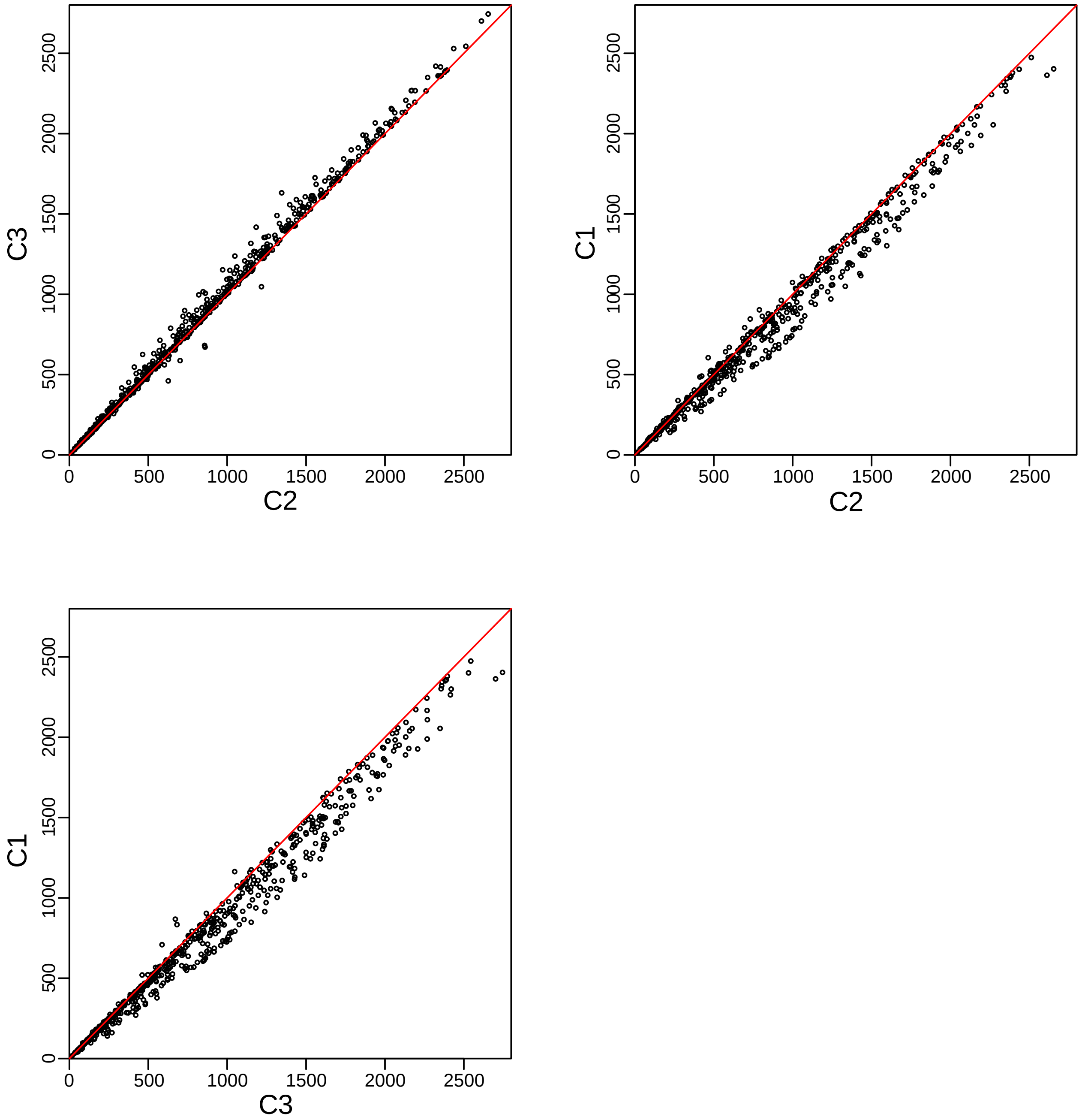

Figure S21. Pairwise comparisons of the calibration sets C1, C2 and C3 (Tab.1). The x and y-axis represent ages in million years. The circles are individual age estimations for a node with a given molecular clock and calibration set.

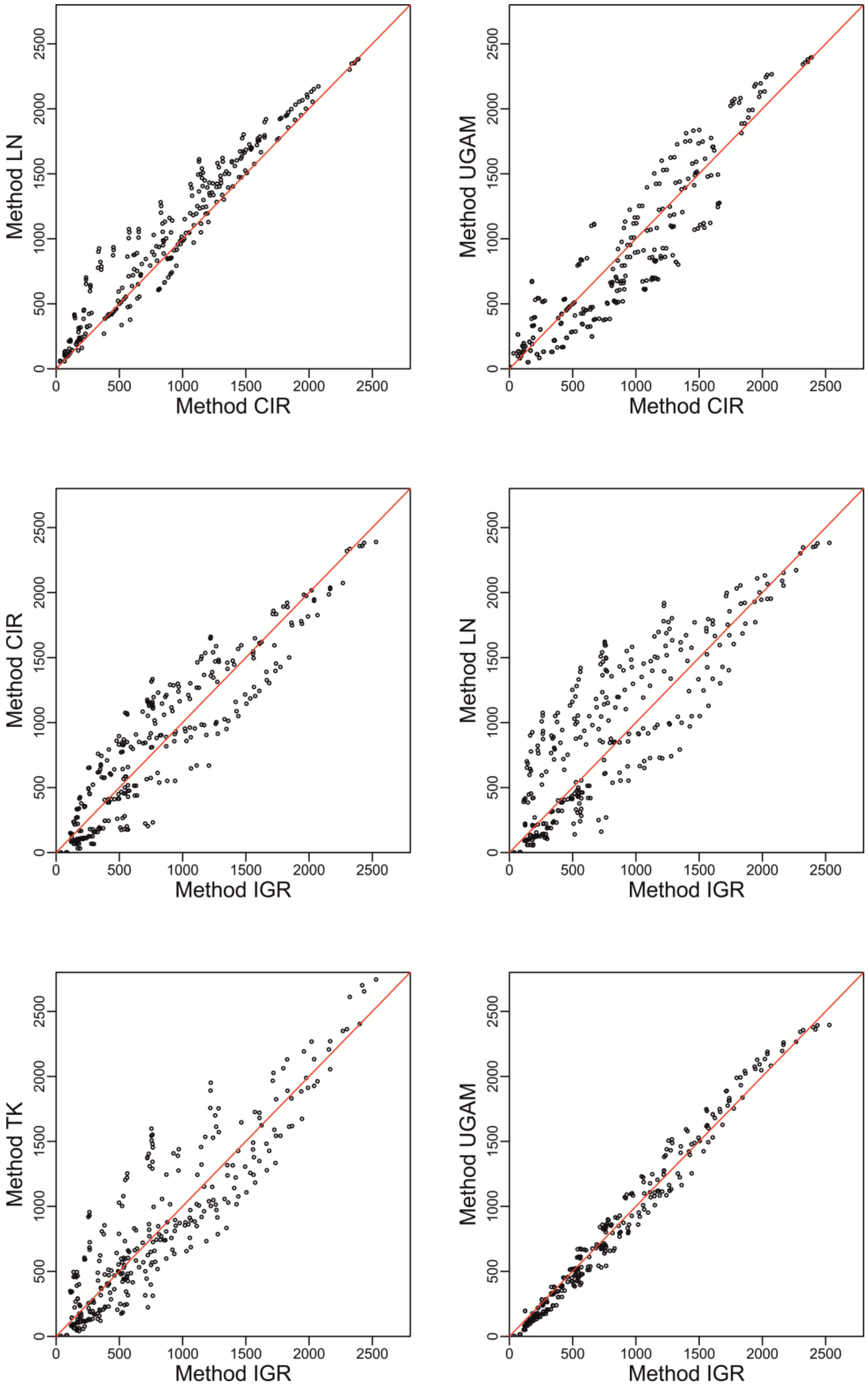

Figure S22. Description below.

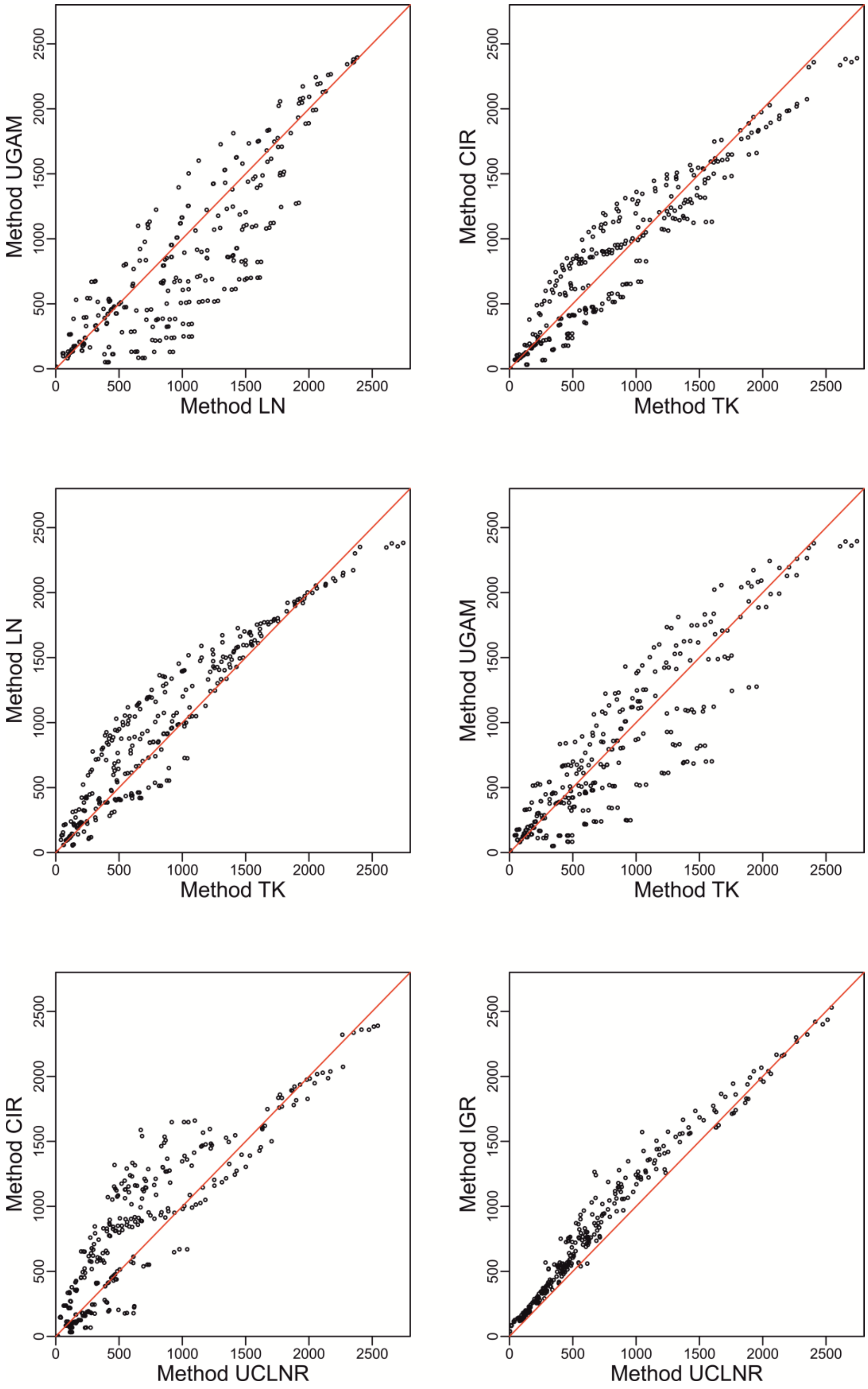

Figure S22. Description below.

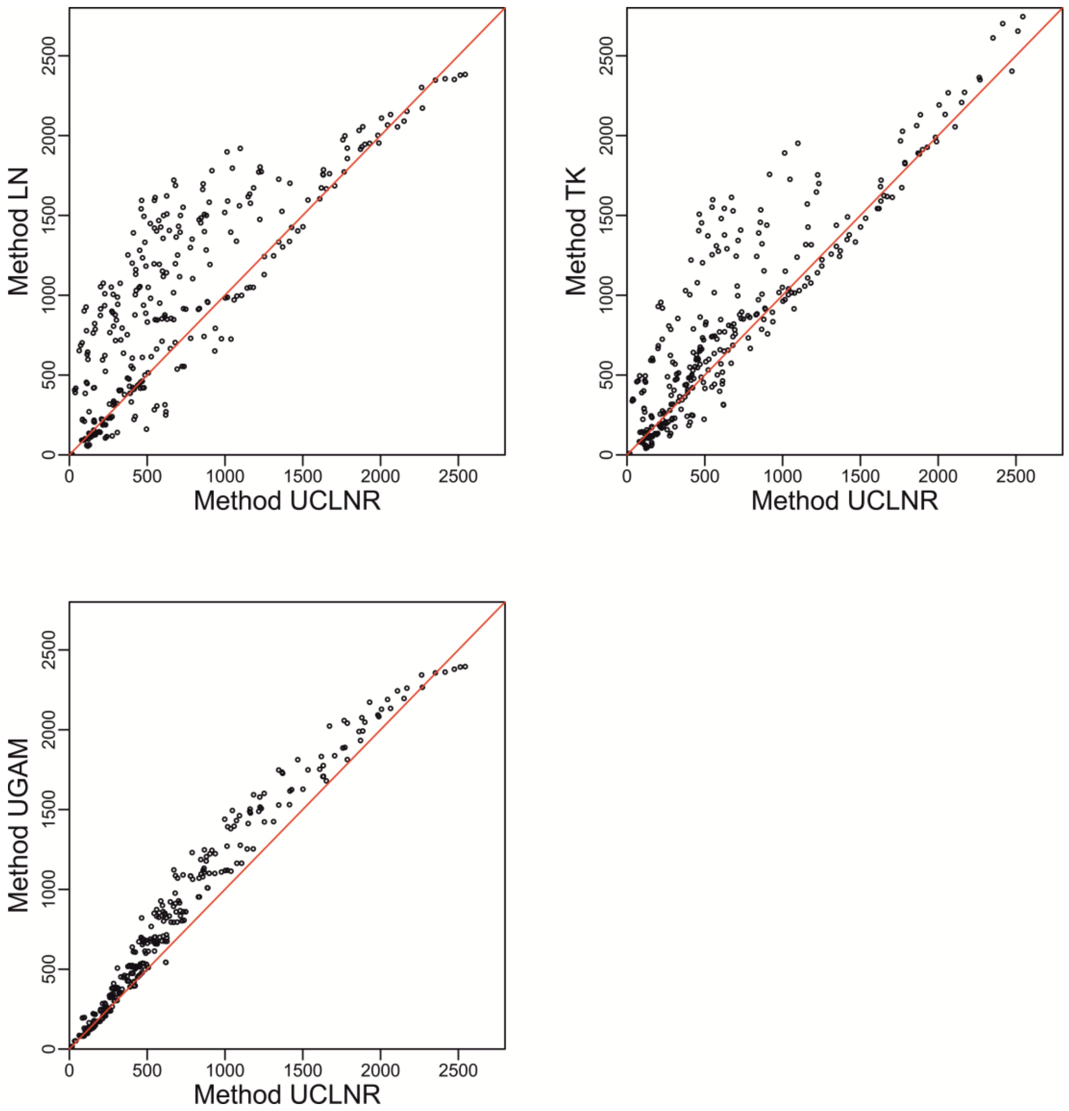

Figure S22. Pairwise comparison between the clocks. The x and y-axis represent ages in million years. The circles are individual age estimations for a node with a given molecular clock and calibration set.

SI References

Agić H., Moczydłowska M., Yin L. 2017. Diversity of organic-walled microfossils from the early Mesoproterozoic Ruyang Group, North China Craton – A window into the early eukaryote evolution. Precambrian Research. 297:101–130.

Bekker A., Holland H.D., Wang P.-L., Rumble D., Stein H.J., Hannah J.L., Coetzee L.L., Beukes N.J. 2004a. Dating the rise of atmospheric oxygen. Nature. 427:117–120.

Bekker A., Holland H.D., Wang P.-L., Rumble D., Stein H.J., Hannah J.L., Coetzee L.L., Beukes N.J. 2004b. Dating the rise of atmospheric oxygen. Nature. 427:117–120.

Bengtson S., Sallstedt T., Belivanova V., Whitehouse M. 2017. Three-dimensional preservation of cellular and subcellular structures suggests 1.6 billion-year-old crown-group red algae. PLoS Biology. 15:e2000735.

Berney C., Pawlowski J. 2006. A molecular time-scale for eukaryote evolution recalibrated with the continuous microfossil record. Proceedings of the Royal Society B: Biological Sciences. 273:1867–1872.

Blank C.E. 2013. Origin and early evolution of photosynthetic eukaryotes in freshwater environments: reinterpreting proterozoic paleobiology and biogeochemical processes in light of trait evolution. Journal of Phycology. 49:1040–1055.

Bouckaert R., Heled J., Kühnert D., Vaughan T., Wu C.-H., Xie D., Suchard M.A., Rambaut A., Drummond A.J. 2014. BEAST 2: A Software Platform for Bayesian Evolutionary Analysis. PLOS Computational Biology. 10:e1003537.

Cather S., Dunbar N., Mcdowell F., McIntosh W., Scholle P. 2009. Climate forcing by iron fertilization from repeated ignimbrite eruptions: the icehouse-silicic large igneous province (SLIP) hypothesis. .

Catling D.C., Zahnle K.J. 2020. The Archean atmosphere. Sci Adv. 6:eaax1420.

Crowe S.A., Døssing L.N., Beukes N.J., Bau M., Kruger S.J., Frei R., Canfield D.E. 2013. Atmospheric oxygenation three billion years ago. Nature. 501:535–538.

Demoulin C.F., Lara Y.J., Cornet L., François C., Baurain D., Wilmotte A., Javaux E.J. 2019. Cyanobacteria evolution: Insight from the fossil record. Free Radical Biology and Medicine. 140:206–223.

Diamond C.W., Lyons T.W. 2018. Mid-Proterozoic redox evolution and the possibility of transient oxygenation events. Emerg Top Life Sci. 2:235–245.

Douzery E.J.P., Snell E.A., Bapteste E., Delsuc F., Philippe H. 2004. The timing of eukaryotic evolution: Does a relaxed molecular clock reconcile proteins and fossils? Proceedings of the National Academy of Sciences. 101:15386–15391.

Ernst R.E., Bond D.P.G., Zhang S.-H., Buchan K.L., Grasby S.E., Youbi N., El Bilali H., Bekker A., Doucet L.S. 2021. Large Igneous Province Record Through Time and Implications for Secular Environmental Changes and Geological Time-Scale Boundaries. Large Igneous Provinces. American Geophysical Union (AGU). p. 1–26.

Foster G.L., Royer D.L., Lunt D.J. 2017. Future climate forcing potentially without precedent in the last 420 million years. Nat Commun. 8:14845.

Franks P.J., Royer D.L., Beerling D.J., Van de Water P.K., Cantrill D.J., Barbour M.M., Berry J.A. 2014. New constraints on atmospheric CO2 concentration for the Phanerozoic. Geophysical Research Letters. 41:4685–4694.

Friis E.M., Pedersen K.R., Crane P.R. 2006. Cretaceous angiosperm flowers: innovation and evolution in plant reproduction. Palaeogeography, palaeoclimatology, palaeoecology. 232:251–293.

Gargaud M., Amils R., Cleaves H.J. 2011. Encyclopedia of astrobiology. Springer Science & Business Media.

Gibson T.M., Shih P.M., Cumming V.M., Fischer W.W., Crockford P.W., Hodgskiss M.S., Wörndle S., Creaser R.A., Rainbird R.H., Skulski T.M. 2018. Precise age of Bangiomorpha pubescens dates the origin of eukaryotic photosynthesis. Geology. 46:135–138.

Han T.M., Runnegar B. 1992. Megascopic eukaryotic algae from the 2.1-billion-year-old negaunee iron-formation, Michigan. Science. 257:232–5.

Hedges S.B., Blair J.E., Venturi M.L., Shoe J.L. 2004. A molecular timescale of eukaryote evolution and the rise of complex multicellular life. BMC evolutionary biology. 4:1–9.

Hermann T.N., Podkovyrov V.N. 2010. A discovery of riphean heterotrophs in the Lakhanda group of Siberia. Paleontological Journal. 44:374–383.

Hoffman P.F., Abbot D.S., Ashkenazy Y., Benn D.I., Brocks J.J., Cohen P.A., Cox G.M., Creveling J.R., Donnadieu Y., Erwin D.H., Fairchild I.J., Ferreira D., Goodman J.C., Halverson G.P., Jansen M.F., Hir G.L., Love G.D., Macdonald F.A., Maloof A.C., Partin C.A., Ramstein

G., Rose B.E.J., Rose C.V., Sadler P.M., Tziperman E., Voigt A., Warren S.G. 2017. Snowball Earth climate dynamics and Cryogenian geology-geobiology. Science Advances. 3:e1600983.

Hofmann H.J. 1976a. Precambrian Microflora, Belcher Islands, Canada: Significance and Systematics. Journal of Paleontology. 50:1040–1073.

Hofmann H.J. 1976b. Precambrian microflora, Belcher Islands, Canada: significance and systematics. Journal of Paleontology.:1040–1073.

Hughes N.F., McDougall A.B., Chapman J.L. 1991. Exceptional new record of Cretaceous Hauterivian angiospermid pollen from southern England. Journal of Micropalaeontology. 10:75–82.

Kalyaanamoorthy S., Minh B.Q., Wong T.K.F., von Haeseler A., Jermiin L.S. 2017. ModelFinder: fast model selection for accurate phylogenetic estimates. Nat Methods. 14:587–589.

Klein C., Beukes N.J., Schopf J.W. 1987. Filamentous microfossils in the early proterozoic transvaal supergroup: their morphology, significance, and paleoenvironmental setting. Precambrian Research. 36:81–94.

Knoll A.H., Strother P.K., Rossi S. 1988. Distribution and diagenesis of microfossils from the lower proterozoic duck creek dolomite, Western Australia. Precambrian Research. 38:257–279.

Kooistra W.H., Medlin L.K. 1996. Evolution of the diatoms (Bacillariophyta): IV. A reconstruction of their age from small subunit rRNA coding regions and the fossil record. Molecular phylogenetics and evolution. 6:391–407.

Krissansen-Totton J., Arney G.N., Catling D.C. 2018. Constraining the climate and ocean pH of the early Earth with a geological carbon cycle model. Proc Natl Acad Sci U S A. 115:4105–4110.

Lanfear R., Frandsen P.B., Wright A.M., Senfeld T., Calcott B. 2017. PartitionFinder 2: New Methods for Selecting Partitioned Models of Evolution for Molecular and Morphological Phylogenetic Analyses. Molecular Biology and Evolution. 34:772–773.

Large R.R., Mukherjee I., Gregory D., Steadman J., Corkrey R., Danyushevsky L.V. 2019. Atmosphere oxygen cycling through the Proterozoic and Phanerozoic. Mineralium Deposita. 54:485–506.

Lenton T.M., Daines S.J., Mills B.J.W. 2018. COPSE reloaded: an improved model of biogeochemical cycling over Phanerozoic time. Earth-Science Reviews. 178:1–28.

Loron C., Moczydłowska M. 2018. Tonian (Neoproterozoic) eukaryotic and prokaryotic organic-walled microfossils from the upper Visingsö Group, Sweden. Palynology. 42:220–254.

Mills B.J.W., Krause A.J., Scotese C.R., Hill D.J., Shields G.A., Lenton T.M. 2019. Modelling the long-term carbon cycle, atmospheric CO2, and Earth surface temperature from late Neoproterozoic to present day. Gondwana Research. 67:172–186.

Minh B.Q., Schmidt H.A., Chernomor O., Schrempf D., Woodhams M.D., von Haeseler A., Lanfear R. 2020. IQ-TREE 2: New Models and Efficient Methods for Phylogenetic Inference in the Genomic Era. Molecular Biology and Evolution. 37:1530–1534.

Moczydłowska M., Landing E.D., Zang W., Palacios T. 2011. Proterozoic phytoplankton and timing of Chlorophyte algae origins. Palaeontology. 54:721–733.

Morris J.L., Puttick M.N., Clark J.W., Edwards D., Kenrick P., Pressel S., Wellman C.H., Yang Z., Schneider H., Donoghue P.C. 2018. The timescale of early land plant evolution. Proceedings of the National Academy of Sciences. 115:E2274–E2283.

Mullins G.L., Servais T. 2008. The diversity of the Carboniferous phytoplankton. Review of Palaeobotany and Palynology. 149:29–49.

Nie Y., Foster C.S.P., Zhu T., Yao R., Duchêne D.A., Ho S.Y.W., Zhong B. 2020. Accounting for Uncertainty in the Evolutionary Timescale of Green Plants Through Clock-Partitioning and Fossil Calibration Strategies. Systematic Biology. 69:1–16.

Page R.W., Jackson M.J., Krassay A.A. 2000. Constraining sequence stratigraphy in north Australian basins: SHRIMP U–Pb zircon geochronology between Mt Isa and McArthur River. Australian Journal of Earth Sciences. 47:431–459.

Parfrey L.W., Lahr D.J.G., Knoll A.H., Katz L.A. 2011. Estimating the timing of early eukaryotic diversification with multigene molecular clocks. Proceedings of the National Academy of Sciences. 108:13624–13629.

Pietrzak-Renaud N., Davis D. 2014. U–Pb geochronology of baddeleyite from the Belleview metadiabase: Age and geotectonic implications for the Negaunee Iron Formation, Michigan. Precambrian Research. 250:1–5.

Robert F., Chaussidon M. 2006. A palaeotemperature curve for the Precambrian oceans based on silicon isotopes in cherts. Nature. 443:969–972.

Ronquist F., Teslenko M., van der Mark P., Ayres D.L., Darling A., Höhna S., Larget B., Liu L., Suchard M.A., Huelsenbeck J.P. 2012. MrBayes 3.2: Efficient Bayesian Phylogenetic Inference and Model Choice Across a Large Model Space. Systematic Biology. 61:539–542.

Royer D.L. 2014. 6.11 - Atmospheric CO2 and O2 During the Phanerozoic: Tools, Patterns, and Impacts. In: Holland H.D., Turekian K.K., editors. Treatise on Geochemistry (Second Edition). Oxford: Elsevier. p. 251–267.

Rubinstein C.V., Gerrienne P., de la Puente G.S., Astini R.A., Steemans P. 2010. Early Middle Ordovician evidence for land plants in Argentina (eastern Gondwana). New Phytologist. 188:365–369.

Rubinstein C.V., Vajda V. 2019. Baltica cradle of early land plants? Oldest record of trilete spores and diverse cryptospore assemblages; evidence from Ordovician successions of Sweden. Gff. 141:181–190.

Sánchez-Baracaldo P., Raven J.A., Pisani D., Knoll A.H. 2017. Early photosynthetic eukaryotes inhabited low-salinity habitats. Proc Natl Acad Sci USA. 114:E7737.

Scotese C.R., Song H., Mills B.J.W., van der Meer D.G. 2021. Phanerozoic Paleotemperatures: The Earth’s Changing Climate during the Last 540 million years. Earth-Science Reviews. 215.

Sensarma S., Storey B., Malviya V.P. 2017. Gondwana Large Igneous Provinces (LIPs): distribution, diversity and significance. Special Publications.

Sims P.A., Mann D.G., Medlin L.K. 2006. Evolution of the diatoms: insights from fossil, biological and molecular data. Phycologia. 45:361–402.

Soltis P.S., Soltis D.E. 2004. The origin and diversification of angiosperms. American Journal of Botany. 91:1614–1626.

Song H., Wignall P.B., Song H., Dai X., Chu D. 2019. Seawater Temperature and Dissolved Oxygen over the Past 500 Million Years. J. Earth Sci. 30:236–243.

Stamatakis A. 2014. RAxML version 8: a tool for phylogenetic analysis and post-analysis of large phylogenies. Bioinformatics. 30:1312–1313.

Steemans P., Le Hérissé A., Melvin J., Miller M.A., Paris F., Verniers J., Wellman C.H. 2009. Origin and radiation of the earliest vascular land plants. Science. 324:353–353.

Strassert J.F.H., Irisarri I., Williams T.A., Burki F. 2021. A molecular timescale for eukaryote evolution with implications for the origin of red algal-derived plastids. Nat Commun. 12:1879.

Strother P.K. 2016a. Systematics and evolutionary significance of some new cryptospores from the Cambrian of eastern Tennessee, USA. Review of Palaeobotany and Palynology. 227:28–41.

Strother P.K. 2016b. Systematics and evolutionary significance of some new cryptospores from the Cambrian of eastern Tennessee, USA. Review of Palaeobotany and Palynology. 227:28–41.

Strother P.K., Foster C. 2021. A fossil record of land plant origins from charophyte algae. Science. 373:792–796.

Tang Q., Pang K., Yuan X., Xiao S. 2020. A one-billion-year-old multicellular chlorophyte. Nature Ecology & Evolution. 4:543–549.

Teyssedre B. 2007. Precambrian palaeontology in the light of molecular phylogeny–an example: the radiation of the green algae. Biogeosciences Discussions. 4:3123–3142.

Tomitani A., Knoll A.H., Cavanaugh C.M., Ohno T. 2006. The evolutionary diversification of cyanobacteria: molecular–phylogenetic and paleontological perspectives. Proceedings of the National Academy of Sciences. 103:5442–5447.

Turnau E., Zavialova N., Prejbisz A. 2009. Wall ultrastructure in some dispersed megaspores and seed-megaspores from the Middle Devonian of northern Poland. Review of Palaeobotany and Palynology. 156:14–33.

Vérard C., Veizer J. 2019. On plate tectonics and ocean temperatures. Geology.

Verbruggen H., Ashworth M., LoDuca S.T., Vlaeminck C., Cocquyt E., Sauvage T., Zechman F.W., Littler D.S., Littler M.M., Leliaert F. 2009. A multi-locus time-calibrated phylogeny of the siphonous green algae. Molecular phylogenetics and evolution. 50:642–653.

Wacey D., Saunders M., Kong C., Brasier A., Brasier M. 2016. 3.46Ga Apex chert ‘microfossils’ reinterpreted as mineral artefacts produced during phyllosilicate exfoliation. Gondwana Research. 36:296–313.

Wang Y., Wang Y., Du W. 2016. The long-ranging macroalga Grypania spiralis from the Ediacaran Doushantuo Formation, Guizhou, South China. Alcheringa: An Australasian Journal of Palaeontology. 40:303–312.

Xiao S., Knoll A.H., Yuan X., Pueschel C.M. 2004. Phosphatized multicellular algae in the Neoproterozoic Doushantuo Formation, China, and the early evolution of florideophyte red algae. American Journal of Botany. 91:214–227.

Yang E.C., Boo S.M., Bhattacharya D., Saunders G.W., Knoll A.H., Fredericq S., Graf L., Yoon H.S. 2016. Divergence time estimates and the evolution of major lineages in the florideophyte red algae. Sci Rep. 6:21361.

Yoon H.S., Hackett J.D., Ciniglia C., Pinto G., Bhattacharya D. 2004. A molecular timeline for the origin of photosynthetic eukaryotes. Mol Biol Evol. 21:809–818.

Zhang Y. 1988. Proterozoic stromatolitic micro-organisms from Hebei, North China: Cell preservation and cell division. Precambrian Research. 38:165–175.
